# Supplementary material for: Consumption of alcohol and binge drinking among pregnant women in Addis Ababa, Ethiopia: Prevalence and determinant factors
Source: PLoS One. 2020 Dec 22;15(12):e0243784. doi: 10.1371/journal.pone.0243784 (PMC7755214; doi:10.1371/journal.pone.0243784)
Supplement: Attachment — Submitted filename: PONE-D-20-19931_R1_reviewer PPW 20201013.pdf [file pone.0243784.s003.pdf]

PLOS ONE

Consumption of Alcohol and Binge Drinking among Pregnant Women in Health Institutions, Addis Ababa, Ethiopia: Prevalence and Determinant factors

--Manuscript Draft--

**Manuscript Number:**	PONE-D-20-19931R1
**Article Type:**	Research Article
**Full Title:**	Consumption of Alcohol and Binge Drinking among Pregnant Women in Health Institutions, Addis Ababa, Ethiopia: Prevalence and Determinant factors
**Short Title:**	Consumption of Alcohol and Binge Drinking among Pregnant Women in Health Institutions, Addis Ababa, Ethiopia
**Corresponding Author:**	Mezinew Sintayehu Bitew<br>Debre Markos University<br>Debre Markos, ETHIOPIA
**Keywords:**	Alcohol, Consumption, Binge, Drinking, Predictors, Pregnancy, Ethiopia
**Abstract:**	Introduction: People in Ethiopia, including pregnant women, highly consume both home-made and manufactured alcohol beverages due to of lack of awareness about the harmful effect of risky alcohol use, and cultural acceptance of alcohol consumption. Alcohol consumption and other hazardous patterns of use like binge drinking have tremendous adverse effects on both the fetus and mother. Therefore, this study aimed to assess the magnitude of alcohol consumption, binge drinking and its determinant factors among pregnant women residing in Kolfe sub-city, Addis Ababa, Ethiopia.<br>Methods: Institutional based cross-sectional study was conducted on a total of 367 pregnant women once they had been selected by using systematic random sampling. Data was collected using a structured questionnaire. A binary logistic regression model, using SPSS version 20 software, was used to identify associated factors. The results were described using adjusted odds ratio with a 95% confidence interval, and statistical significance was considered at p-value < 0.05.<br>Results: This study revealed that the prevalence of alcohol consumption, binge drinking, and weekly alcohol consumption of four or more units among pregnant women was 39.78%, 3.54% and 4.9% respectively. Factors such as not having formal education [AOR 95% CI=8.47(2.42, 29.62), having primary education [AOR 95% CI=4.26(1.23, 14.74), being a housewife[AOR 95% CI= 4.18(2.13,8.22), having an unplanned pregnancy [AOR 95% CI=2.47(1.33, 4.60), having a history of abortion[AOR 95% CI=3.33(1.33, 6.05)], not having awareness about the harmful effect of alcohol consumption [AOR 95% CI=4.66(2.53,8.61)], and not having family social support [AOR 95% CI= 2(1.14,3.53) were found to be significantly associated with alcohol consumption among pregnant women.<br>Conclusions: This study found that there is a high level of alcohol consumption. Prevention and intervention that focuses on creating awareness about the harmful effects of alcohol, strengthening social support during pregnancy, and family planning policy and services that can reduce unplanned pregnancy and abortion should have developed and implemented.<br>Keywords: Alcohol, Consumption, Binge, Drinking, Predictors, Pregnancy, Ethiopia
**Order of Authors:**	Mezinew Sintayehu Bitew
	Maereg Fekade
	Muluken Wubetu
	Addisu Alehegn Alemu
**Response to Reviewers:**	Thank you so much all of you, editors and the two reviewers, for reviewing the manuscript critically for the sake of publishing quality scientific research.<br>Response to academic editor's comment and questions<br>1.Please ensure that your manuscript meets PLOS ONE's style requirements, including those for file naming. The PLOS ONE style templates can be found<br>Answer: I made the manuscript in line with the requirements of PIOS ONE<br>2.For a question -During our internal evaluation we noted that participants provided verbal consent, did the ethics committees/IRBs approve this consent procedure?

Answer: in the actual procedure of the work, a copy of the master thesis, mother document of this manuscript, it states that both verbal and written informed consent was obtained. Data collectors explained to participants about the objective of the study, procedures, and their right to refuse or discontinue the interview at any time and other important information. After participants heard and understood all the necessary information and consent statement, they have been asked about their willingness to be interviewed, which is verbal consent. Those volunteers were again asked to write their name and put their signature with the date of interview at a prepared blank space found below the information sheet and statement of consent, which is written consent. That means they provide both verbal and written informed consent. So, in the manuscript that states "only verbal consent" was an error and misunderstanding when it was written in research ethics subheading. Now, I accepted your comments and took a correction in this revised manuscript.
3.Contradictory of the message regarding pregnant women under age 18 in the section of exclusion criteria and ethical consideration.
Answer: Yes, pregnant women start from 15 years old were enrolled in the study. Therefore, I removed the message that excludes women under 18 years old from the section of exclusion criteria.
4.In the abstract it is stated that the study "was conducted from April to June 2017", but in the methods section of the manuscript it is stated that the study "was conducted from May to June 2017".
Answer: the right period of data collection was from May to June 2017. I amended it.
5.Requested to update ORCID ID.
Answer: I did it.

Response for Reviewers
Reviewer 1
1.Are there national survey data (prevalence rates) for alcohol/drug use in the general population that can be included in the Background section, to provide some context for why this is a public health concern in Ethiopia?
Answer: There are only local single articles in a different part of the country, and all reports that it the public health problems like the other countries across the world. But, there is no national survey study.
2.Strong comments in method section regarding how data collected, how women approached, how the research described, and about the destination of pregnant women who used alcohol in a risky way
Answer: I accept all the comments and incorporate the raised concerns. Data collectors explained the objective and procedures of the study, and their right to refuse or discontinue the interview at any time to participants. Participants were aware of as the study has not any risk or direct benefit like incentives for them; it was only conducted for the achievement of objectives, and ultimately for improving health services of pregnant women and the community as a whole. After that, data collectors interviewed the selected participant in the face to face manner by using a structured questionnaire at the waiting room of the maternal care clinic that took around 15 minutes. Those who found risky of alcohol consumption has been linked to substance abuse clinic, and responsible leaders in selected health centers and maternal care clinics were recommended and noticed to initiate and strengthen prevention of alcohol consumption among pregnant women.
3.A question regarding missing data?
Answer: From the beginning, we have been adding 10% of possible non-response participants on the calculated samples. The non-responses in a manuscript were refusals to participate but not missing data's/values. Fortunately, the actual non-response rate was below the prior plan or expected non-response rate that couldn't affect the analysis and conclusion.
4.A comment of adding stigma or any other thing that could hinder the disclosure of alcohol use.
Answer: I understand your concern, and I try to add some possible perceived stigma. But, as I mentioned in the background of the study, there is a cultural acceptance of alcohol use in all demographic population. Since alcohol is historical and frequently used in our culture, which may be associated with Orthodox Christianity religion, stigma and punishment are not found.

Reviewer 2
Answer: I saw all questions, comment and suggestion on each page of the document

Powered by Editorial Manager® and ProduXion Manager® from Aries Systems Corporation

	or manuscript that you gave me. I thank you for all you did on a manuscript. And I found it is essential for me and scientific research as well. I accepted all the comments and suggested solutions that you wrote and marked, and I corrected it in a document accordingly. I also received a note that you told me to incorporate the independent sub-heading variable (social support) with the next subheading in table four (associated factors- subheadings). But, I didn't do for the variable of awareness about the harmful effect of alcohol consumption because this has a figure that describes and presents a descriptive message different from table four (associated factors). And I minimized the method sub-headings and merged it.
**Additional Information:**	
**Question**	**Response**
**Financial Disclosure**<br><br>Enter a financial disclosure statement that describes the sources of funding for the work included in this submission. Review the submission guidelines for detailed requirements. View published research articles from *PLOS ONE* for specific examples.<br><br>This statement is required for submission and **will appear in the published article** if the submission is accepted. Please make sure it is accurate.<br><br>**Unfunded studies**<br>Enter: *The author(s) received no specific funding for this work.*<br><br>**Funded studies**<br>Enter a statement with the following details:<br>• Initials of the authors who received each award<br>• Grant numbers awarded to each author<br>• The full name of each funder<br>• URL of each funder website<br>• Did the sponsors or funders play any role in the study design, data collection and analysis, decision to publish, or preparation of the manuscript?<br>• **NO** - Include this sentence at the end of your statement: *The funders had no role in study design, data collection and analysis, decision to publish, or preparation of the manuscript.*<br>• **YES** - Specify the role(s) played.<br><br>* typeset	The authors received no specific funding for this work.
**Competing Interests**<br><br>Use the instructions below to enter a	The authors have declared that no competing interests exist.

competing interest statement for this submission. On behalf of all authors, disclose any competing interests that could be perceived to bias this work—acknowledging all financial support and any other relevant financial or non-financial competing interests.<br><br>This statement **will appear in the published article** if the submission is accepted. Please make sure it is accurate. View published research articles from *PLOS ONE* for specific examples.<br><br>**NO authors have competing interests**<br><br>Enter: *The authors have declared that no competing interests exist.*<br><br>**Authors with competing interests**<br><br>Enter competing interest details beginning with this statement:<br><br>*I have read the journal's policy and the authors of this manuscript have the following competing interests: [insert competing interests here]*<br><br>* typeset	
**Ethics Statement**<br><br>Enter an ethics statement for this submission. This statement is required if the study involved:<br><br>• Human participants<br>• Human specimens or tissue<br>• Vertebrate animals or cephalopods<br>• Vertebrate embryos or tissues<br>• Field research<br><br>Write "N/A" if the submission does not require an ethics statement.<br><br>General guidance is provided below. Consult the submission guidelines for detailed instructions. **Make sure that all information entered here is included in the**	Ethical clearance was obtained from Addis Ababa University, public health school ethical review committee. Both Verbal and written informed consent were also obtained from participants. They were informed that participation is based on a voluntary basis, and had the full right to withdraw at any time during the interview process. For participants under 18 years old, informed consent was also obtained from their parents.

**Methods section of the manuscript.**<br><br>**Format for specific study types**<br><br>**Human Subject Research (involving human participants and/or tissue)**<br>• Give the name of the institutional review board or ethics committee that approved the study<br>• Include the approval number and/or a statement indicating approval of this research<br>• Indicate the form of consent obtained (written/oral) or the reason that consent was not obtained (e.g. the data were analyzed anonymously)<br><br>**Animal Research (involving vertebrate animals, embryos or tissues)**<br>• Provide the name of the Institutional Animal Care and Use Committee (IACUC) or other relevant ethics board that reviewed the study protocol, and indicate whether they approved this research or granted a formal waiver of ethical approval<br>• Include an approval number if one was obtained<br>• If the study involved *non-human primates*, add *additional details* about animal welfare and steps taken to ameliorate suffering<br>• If anesthesia, euthanasia, or any kind of animal sacrifice is part of the study, include briefly which substances and/or methods were applied<br><br>**Field Research**<br><br>Include the following details if this study involves the collection of plant, animal, or other materials from a natural setting:<br>• Field permit number<br>• Name of the institution or relevant body that granted permission	
**Data Availability**<br><br>Authors are required to make all data underlying the findings described fully available, without restriction, and from the time of publication. PLOS allows rare exceptions to address legal and ethical concerns. See the PLOS Data Policy and FAQ for detailed information.	Yes - all data are fully available without restriction

A Data Availability Statement describing where the data can be found is required at submission. Your answers to this question constitute the Data Availability Statement and **will be published in the article**, if accepted.<br><br>**Important:** Stating 'data available on request from the author' is not sufficient. If your data are only available upon request, select 'No' for the first question and explain your exceptional situation in the text box.<br><br>Do the authors confirm that all data underlying the findings described in their manuscript are fully available without restriction?	
**Describe where the data may be found in full sentences. If you are copying our sample text, replace any instances of XXX with the appropriate details.**<br><br>• If the data are **held or will be held in a public repository**, include URLs, accession numbers or DOIs. If this information will only be available after acceptance, indicate this by ticking the box below. For example: *All XXX files are available from the XXX database (accession number(s) XXX, XXX.*).<br>• If the data are all contained **within the manuscript and/or Supporting Information files**, enter the following: *All relevant data are within the manuscript and its Supporting Information files.*<br>• If neither of these applies but you are able to provide **details of access elsewhere**, with or without limitations, please do so. For example:<br><br>*Data cannot be shared publicly because of [XXX]. Data are available from the XXX Institutional Data Access / Ethics Committee (contact via XXX) for researchers who meet the criteria for access to confidential data.*<br><br>*The data underlying the results*	All relevant data are within the manuscript and its Supporting Information files.

Powered by Editorial Manager® and ProduXion Manager® from Aries Systems Corporation

presented in the study are available from (include the name of the third party and contact information or URL).<br>• This text is appropriate if the data are owned by a third party and authors do not have permission to share the data.<br><br>* typeset	
Additional data availability information:	

Manuscript

Click here to access/download;Manuscript;Consumption of alcohol and binge drinking manuscript.docx

Consumption of alcohol and binge drinking among pregnant women in health institutions, Addis Ababa, Ethiopia: prevalence and determinant factors

Mezinew Sintayehu Bitew[1], Maereg Fekade Zewude[2], Muluken Wubetu[3], Addisu Alehegn Alemu[4]

[1]Department of mental health, Debre Markos University, Debre Markos, Amhara, Ethiopia

[2] Department of Maternal and Child Health, Addis Ababa City Administration Health Bureau, Addis Ababa, Ethiopia

[3]Department of Pharmacy, Debre Markos University, Debre Markos, Amhara, Ethiopia.

[4]Department of midwifery, Debre Markos University, Debre Markos, Amhara, Ethiopia.

Corresponding Author

E-mail: sintayehume@gmail.com (MS)

Abstract

Introduction: People in Ethiopia, including pregnant women, highly consume both home-made and manufactured alcohol beverages due to of lack of awareness about the harmful effect of risky alcohol use, and cultural acceptance of alcohol consumption. Alcohol consumption and other hazardous patterns of use like binge drinking have tremendous adverse effects on both the fetus and mother. Therefore, this study aimed to assess the magnitude of alcohol consumption, binge drinking and its determinant factors among pregnant women residing in Kolfe sub-city, Addis Ababa, Ethiopia.

Methods: Institutional based cross-sectional study was conducted on a total of 367 pregnant women once they had been selected by using systematic random sampling. Data was collected using a structured questionnaire. A binary logistic regression model, using SPSS version 20 software, was used to identify associated factors. The results were described using adjusted odds ratio with a 95% confidence interval, and statistical significance was considered at p-value < 0.05.

Results: This study revealed that the prevalence of alcohol consumption, binge drinking, and weekly alcohol consumption of four or more units among pregnant women was 39.78%, 3.54% and 4.9% respectively. Factors such as not having formal education [AOR 95% CI=8.47(2.42, 29.62), having primary education [AOR 95% CI=4.26(1.23, 14.74), being a housewife[AOR 95% CI= 4.18(2.13,8.22), having an unplanned pregnancy [AOR 95% CI=2.47(1.33, 4.60), having a history of abortion[AOR 95% CI=3.33(1.33, 6.05)], not having awareness about the harmful effect of alcohol consumption [AOR 95% CI=4.66(2.53,8.61)], and not having family social support [AOR 95% CI= 2(1.14,3.53) were found to be significantly associated with alcohol consumption among pregnant women.

Conclusions: This study found that there is a high level of alcohol consumption. Prevention and intervention that focuses on creating awareness about the harmful effects of alcohol, strengthening social support during pregnancy, and family planning policy and services that can reduce unplanned pregnancy and abortion should have developed and implemented.

Keywords: Alcohol, Consumption, Binge, Drinking, Predictors, Pregnancy, Ethiopia

Introduction

Globally, use of substances such as alcohol, tobacco and other illicit substances, in a way that could harm the users' health, have escalated through time in all segments of the population and has become one of the growing public health and socioeconomic concerns. From these substances, alcohol shares the biggest burden that accounts for 3.8% of all global deaths and 4.6% of the global burden of diseases and neuropsychiatric disorders [1–3]. Risky use of alcohol causes significant morbidity and mortality (particularly from injuries) and societal harm such as social disruption from crime, unemployment and marital disharmony [2, 3].

In pregnant women, the negative health consequences of alcohol consumption are more profound for themselves as well as for the fetus. Alcohol consumption during pregnancy is also showed to be one of the leading causes of preventable birth defects and developmental problems including language and motor delays and poor academic achievement [4–6].

New studies indicate that even low levels of prenatal alcohol exposure could adversely affect the developing fetus. Cognitive and socio-emotional deficits, among children exposed to even small amounts of alcohol, are found [7]. Consumption of <_ 2 or more standard drink of alcohol/day in early pregnancy is associated with significant problems on the fetus that can cause miscarriage , preterm birth, low birth weight and low birth centile compared to children born from non-drinkers.

In addition, other studies reported that women who consumed more than three drinks per week during the first trimester had a significant negative health effect on pregnancy, and the effect would be five folds more if they took five or more units of alcohol per week [8–12]. Furthermore, binge-drinking (commonly defined as consuming 4 or more units of alcohol at one occasion for women) during pregnancy is more harmful because of the serious toxic effects on neurodevelopment after it crosses the placenta [13, 14].

Adverse effects of prenatal alcohol exposure can range from subtle developmental problems, or fetal alcohol effects, to full-blown fetal alcohol spectrum syndrome (FASS). However, the effects of alcohol exposure are not limited to infancy and childhood; prenatal exposure to alcohol, particularly in early pregnancy, has also been found to increase the likelihood of developing an alcohol disorder in adulthood [15, 16].

Given these consequences and the belief that there is no safe time or amount to consume alcohol in pregnancy, the CDC recommends complete abstinence [17, 18].

Despite this, studies from various high income and low-income countries have reported a high rate of alcohol consumption by pregnant women from 19.5% in South Africa to 36.3% in Geneva [19-23]. The prevalence showed that 26.5%, 28.5%, 36.3%, 19.6%, 25%, and 32.5% in Russia, United Kingdom, South Africa Uganda and Congo respectively [19-23]. The Higher prevalence is also reported from countries like Ghana, Australia, and Nigeria (48%, 56% and 59% respectively) [24–26].

Studies on the prevalence of binge drinking among pregnant women also reported that ranges from 3% in Canada [27] to 25% far high in Congo [3, 27-31]. The amount of drinking per week, which is the other clinically significant patterns of alcohol use, revealed in literature like in New Zealand

study which states that 10% of pregnant women drank seven and more units of alcohol per week as well [30].

Studies from different countries showed that socio-demographic factors (age, marital status, educational status, and occupation), obstetric history (gestational age, unplanned pregnancy, gravidity, and history of abortion), social support and behavioural issues like use of Khat and cigarette smoking were predictors for the consumption of alcohol during pregnancy [32–37]. In addition, studies reported that awareness about the harmful effect of alcohol on fetus, attitude and culture of the population were the determinant factors for alcohol consumption [36, 38–40].

Because of the rapid increase of alcohol consumption and its terrific associated problems among pregnant women across the world, studies of high-income countries on alcohol and its effect are increased, and the interventions for alcohol problems are also developing [41].

In low-income countries including Ethiopia, the rapid increase of the consumption of alcohol has also been indicated around two decades ago, and it is still increasing alarmingly and, especially women of fertile age have affected by alcohol [1, 42]. Both traditional and commercially produced alcohol beverages are widely spread more than ever with the face of lack of awareness about the harmful effect of alcohol, and other predictors of alcohol consumption [1, 38].

People in Ethiopia that include pregnant women highly consume alcohol beverages, especially the culturally accepted traditionally prepared alcohol beverages like Areki, Teje, Tella that have a higher percentage of alcohol amount [43–45]. Thus, locally made alcohols are produced excessively without control and are usually used in different events like celebrities, ceremonies, holidays, parties, and almost in daily family meals and day to day life [43–45].

Despite the above-mentioned problems, assessment of alcohol consumption with its determinant factors upon pregnant women is limited. In particular, there is a scarcity of study on binge drinking

of alcohol by measuring or calculating the amount of alcohol consumed. Addis Ababa, the capital city of Ethiopia where the study is conducted, is heavily affected by hazardous and harmful consumption of alcohol due to both modernization and the deeply rooted cultural acceptance of alcohol consumption [1].-Therefore, given the above background, this study was intended to assess the magnitude of alcohol consumption and binge drinking among pregnant women, and its determinant factors in Kolfe sub city, Addis Ababa, Ethiopia. The finding of this study will have significant contributions for health program managers, policymakers, clinicians and researchers.

Materials and Methods

Study design, sample size and sampling technique

An institutional-based quantitative cross-sectional study based on the guidelines of Strengthening the Report of Observational Studies in Epidemiology (STROBE) for observational research [46] was conducted at Kolfe Keranio sub-city, Addis Ababa, Ethiopia. Kolfe Keranio sub-city, with an area of 61.25 sq.km, has a total population of 546, 219 (235, 360 were female) in 2017. Among the female population, around 2.33 % (9,531) were pregnant. The sub-city has 15 districts and 11 Health Centers (HCs) [47].

A total of 397 pregnant mothers were included in a study after calculating by using single population formula ($n = Z\ a_{/2}^{2} * p * (1-p)/d2$) by taking proportion (p) of alcohol use of pregnant women (34%) [48], 5% margin of error (d) with 95% confidence interval, and by adding 15%, the assumption of non-response rate. Systematic random sampling with ‘k’ value 20 was carried out after samples were proportionally allocated to each of the following 11 health centers: Woreda 1 HC (600*), Woreda 3 HC (1002*), Alem Bank HC (960*), Kolfe HC (846*), Woreda 5 HC (716*),

Woreda 6 HC (426*), Keranio HC (798*), Woreda 11 HC (832*), Lomi Meda HC(540*), Woreda 9 HC (470*), and Mikililand HC (880*). Pregnant women who were in all trimesters, and attended the above-mentioned health centers during the study period were included in the study. Pregnant women who were seriously ill and unable to respond were excluded. The study was conducted from May to June 2017.

Data collection questionnaire/instruments and procedure

Questions regarding socio-demographic, obstetric variables as well as those on alcohol consumption, awareness about the harmful effect of alcohol and the use of other substances like Khat chewing and cigarette smoking were gathered, adapted and developed from various literature.

Family social support was assessed by the Multidimensional Scale of Perceived Social Support (MSPSSf). MSPSS has three subscales (family, friends and significant others), with a total of 12 items each scaled from 1 (very strongly disagree) to 7 (very strongly agree) [49, 50]. Respondents scored higher than 14 from a multidimensional scale of perceived family social support (MSPSSF) 7 Likert scale were recognized as having family social support, those who scored less than or equal to 14 were not having social support.

Questionnaire regarding the amount and frequency of alcohol consumption per day, week, and month, and generally in the time of pregnancy was adapted from AUDIT and other articles [13, 21, and 51]. In Ethiopian, both traditional (e.g. Tella, Araki, Teje) and manufacture (Beer, Wine) alcohol beverages are widely produced and consumed. People used local equipment called Tella Birecheko, Melekia, and Birelie to drink traditional alcohols that can measure around 340 ml, 40 ml and 270 ml volume, respectively. Pregnant women were also asked to estimate the average number of drinks, and the data was standardized into a standard unit of alcohol, by using the

percentage of alcohol and volume in ml. In this study, alcohol consumption was defined as a pregnant woman who drank any amount of alcohol during the period of pregnancy. Whereas, drinking four more units of alcohol on one occasion was considered binge drinking.

Six questions were developed to assess the awareness of the harmful effect of alcohol on fetus, and those who answered the six questions correctly identified as having awareness about the consequences of alcohol.

Furthermore, any amount of khat use and cigarette smoking even at once during the current pregnancy is known as pregnant women who chew khat and smoke cigarette, respectively. The questionnaire was prepared initially in English and translated to Amharic then back to English to check the consistency of the questions. The training was given to the data collectors and supervisors for three days about the data collection tool, way of interview, the ethical principles of confidentiality and data management before their involvement with data collection.

Eleven BSc nurses with BSc degree qualification as data collectors to collect data and two public health professionals as supervisors to assist and monitor the data collection process were employed. Ethical clearance was obtained from Addis Ababa University, public health school ethical review committee. The right participants were systematically selected by data collectors when pregnant women came to maternal care. Data collectors explained the objective and procedures of the study, and their right to refuse or discontinue the interview at any time to participants. And, they knew that the questionnaire was anonymous and their privacy kept confidential. Also, Participants were aware of as the study has not any risk or direct benefit like incentives for them; it was only conducted for the achievement of objectives, and ultimately for improving health services of pregnant women and the community as a whole. After participants heard and understood all the necessary information and consent statement, they have been asked

about their willingness to be interviewed, which was verbal consent. Those volunteers were again asked to write their name and put their signature with the date of interview in a prepared blank space found below the information sheet and statement of consent, which was written consent. For participants under 18 years old, informed consent was also obtained from their parents.

After that, data collectors interviewed the selected participant in the face to face manner by using a structured questionnaire at the waiting room of the maternal care clinic that took around 15 minutes. Those who found risky of alcohol consumption has been linked to substance abuse clinic, and responsible leaders in selected health centers and maternal care clinics were recommended and noticed to initiate and strengthen prevention of alcohol consumption among pregnant women. To control the quality of data, on top of providing training for data collectors and supervisors, supervision was held regularly during the data collection period, and the collected data were checked daily for completeness and consistency. And, Manual and computerized data cleaning were done before data analysis.

Data processing and analysis

Data were checked, cleaned and entered into Epi.DATA 3.1 versions and then exported into Statistical Package for the Social Sciences (SPSS) window version 20 for analysis. Descriptive summary using frequencies, percentage, graphs, the mean and standard deviation were used to present study results. Binary logistic regression was employed to calculate the adjusted odds ratio (AOR) to identify independent determinant factors of the outcome variable. Variables which met p-value < 0.2 during bivariate analysis were exported to the multivariate analysis model. The findings were described with Odds ratio (crude and adjusted) with corresponding 95% Confidence interval. P-value of < 0.05 was considered as statistically significant during the final multivariable binary logistic regression model.

Results and discussion

Results

Description of socio-demographic characteristics

The response rate was 92.44%. Out of 367 pregnant women, 74.7% (274) were married. Two hundred sixty (70.8%) were non-educated, and women who completed secondary education and above were 26 (7.1%). The mean age of respondents was 27.43± SD4.775 years (ranging from 15-49).

In terms of ethnicity, Amhara and Oromo comprise the majority, 114 (31.1%) and 107(29.2%) respectively. Near to half of the participants, 117(48.2%) were followers of orthodox Christian followed by Muslims (35.7%). Majority of pregnant women (42.5%) were housewives while about one-third of them had a monthly average income between 1001-2000ETB **(Table 1)**.

Table 1: Socio-demographic characteristics of pregnant women in Kolfe Keranio sub city (n=367), Addis Ababa, Ethiopia

Variables	Frequency	Percent (%)
**Age**		
15-19	8	2.2
20-24	100	27.2
25 -29	117	31.9
29-34	106	28.9
>35	36	9.8
Mean (SD)	27.43±4.775	
**Marital status**		

Married	247	67.3
single	81	22.1
Divorce	31	8.4
Widowed	8	2.2
**Ethnicity**		
Amhara	114	31.1
Oromo	107	29.2
Gurage	91	24.8
Tigrie	32	8.7
Others	23	6.3
**Religion**		
Orthodox	177	48.2
Islam	131	35.7
Protestant	40	10.9
Catholic	10	2.7
Others	9	2.5
**Educational status**		
Not educated	127	34.6
Primary educated	123	33.5
Secondary educated	91	24.8
>Secondary educated	26	7.1
**Occupation**		
Employed	105	28.6

Housewife	156	42.5
Own business	106	28.9
**Average monthly income of the house hold**		
≤ 1000 Birr	44	12
1001-2000	109	29.7
2001-3000	76	20.7
3001-4000	61	16.6
>4000	77	21

Assessment of obstetric history of respondents

As shown in **Table 2** below, the majority of the pregnant women 148(40.3%) were in the second trimester. Almost three quarter (73%) of the pregnant women have had planned pregnancy. Two hundred fifty-one (68.4%) of pregnant women had no history of abortion**.**

Table 2: Obstetric history of the respondents among pregnant women in kolfe Keranio sub city (n=367) Addis Ababa, Ethiopia

Variables	Frequency	Percent (%)
**Trimester of pregnancy**		
First timester	38	10.4
Second trimester Third trimester	148	40.3
	131	35.7
**Number of pregnancy(gravidity)**		
First time	88	24
Second time	148	40.3

>_ three times	131	35.7
**Number of alive children**		
No child	112	30.5
One	134	36.5
Two	65	17.7
>two	56	15.3
**Planned pregnancy**		
Yes	268	73
No	99	27
**History of abortion**		
Yes	116	31.6
No	251	68.4

Though most of the participants were in the second and third trimester, the proportion of alcohol consumption value was high among first trimester 21 (55.26%) **(Fig 1)**.

Fig. 1: Alcohol consumption among pregnant women respected to trimesters of pregnancy in Kolfe Keranio sub city (n=367), Addis Ababa, Ethiopia

Alcohol consumption, binge drinking and other substances during pregnancy

As illustrated in **(Table 3)** below, 146(39.8%) of pregnant women drank alcohol. The majority (44.4%) of participants predominately drank Tella. Fifty-four (14.71%) consumed alcohol 2-4 times per month while 15 (4.09%) of pregnant women drank four and above times per week. On the other hand, 13 (3.54%) of them had the experience of binge drinking during pregnancy

Table 3: Alcohol consumption and binge drinking among pregnant women in kolfe sub city (n=367), Addis Ababa, Ethiopia

**Variables**	**Frequency**	**Percent (%)**
**Alcohol consumption before pregnancy**		
Yes	179	48.8
No	188	51.2
**Alcohol consumption during pregnancy**		
Yes	146	39.8
No	221	60.2
**Binge drinking during pregnancy**		
Yes	13	3.54
No	354	96. 46
**Frequency of alcohol consumption during pregnancy**		
<_ once a month	36	9.81
2-4 times per month	54	14.71
2-3times per week	41	11.17
4 and above per week	15	4.09
**Type of alcohol use**		
Tella	65	44.4
Teje	9	6.3
Areki	14	9.7
Beer	41	28.5
Wine	9	6.3
Others	7	4.9

**Cigarette smoking during pregnancy**		
Yes	10	2.7
No	357	97.3
**Khat use during pregnancy**		
Yes	39	10.6
No	328	89.4

Awareness about the harmful effect of alcohol use

As demonstrated in **Fig 2**, Two hundred forty-four (66.5%) pregnant women have awareness, answered all six questions correctly regarding the harmful effect of alcohol consumption. Among those who have awareness, only 69 (28.28%) consumed alcohol. Whereas, of those who answered the awareness questions incorrectly, 76 (62.6%) drank alcohol.

Fig 2: Magnitude of alcohol consumption among pregnant women respected to awareness about the harmful effect of alcohol on fetus in Kolfe Keranio sub city (n=367), Addis Ababa, Ethiopia

Factors associated with alcohol consumption among pregnant women

As shown in **Table 4**, educational status, occupation, plan of pregnancy, history of abortion, awareness about the harmful effect of alcohol, and family social support of pregnant women were significant predictors of alcohol consumption among pregnant women with p-value<0.05. Statistically, significant higher alcohol consumption rate was observed among non-educated pregnant women and those who found in primary education compared to pregnant women who completed secondary education and above with (AOR 95%CI= 8.47(2.42, 29.62) and AOR 95%CI= 4.26(1.23, 14.74) respectively.

Pregnant women who were in some form of occupation appeared to have a decreased risk of alcohol consumption compared to those who were housewife that had a fourfold risk of alcohol consumption with (AOR 95% CI=4.18(2.13, 8.22). Respondents who have had unplanned current pregnancy had a twofold prone to alcohol consumption than who had planned pregnancy (AOR 95% CI=2.47(1.33, 4.60).

Those pregnant women that had a history of abortion experienced three-fold risk for alcohol consumption compared to pregnant women who hadn't a history of abortion with [AOR 95% CI=3.33(1.33, 6.05)]. Women who answered correctly about the effect of alcohol on fetus and them also found to be a protective factor for alcohol consumption compared to those who answered incorrectly which become a risk factor [AOR 95% CI 4.66 (2.53, 8.61)]. Moreover, in assessment family social support, more than a half (66.5%) scored greater than or equal to 14 from the total score of MSPSSf. And the study found a relationship between family social support and alcohol consumption; pregnant women who hadn't family social support were two-fold risks for alcohol consumption [AOR 95% CI= 2(1.14,3.53) compared to those who had family social support.

Table 4: Factors associated alcohol use of pregnant women in Kolfe Keranio sub city (n=367), Addis Ababa, Ethiopia

	**Alcohol consumption during pregnancy**			
**Variables**	**Yes**	**No**	**Crude OR (95% CI)**	**Adjusted OR (95% CI)**
**Religion**				
Orthodox	76	101	-----------------	------------------------
Islam	43	88	0.65 (0.41,1.04)	1.25(042,2.13)
Protestant	24	16	1.99(0.99,4.01)	3.01(0.96,5.02)

Catholic	2	8	0.33(0.69,1.61)	0.23(0.11, 1.71)
Others	1	8	0.166 (0.02,1.360	7.09 (0.68, 18.4)
**Educational status**				
Not educated	65	62	4.40(1.56,12.40)	8.47(2.42,29.62)*
Primary education	61	62	4.13(1.47,11.66)	4.26(1.23,14.74)*
Secondary education	15	76	0.83(0.27,2.54)	1.41(0.38,5.23)
>secondary education	5	21	-----------------	-------------------------
**Occupation**				
Employed	34	71	-------------------	-----------------
housewife	87	69	2.63(1.57,4.41)	4.18(2.13,8.22)*
own business	25	81	0.65(0.35,1.18)	0.76(0.35,1.66)
**Plan of pregnancy**				
Yes	85	183	------------------	------------------
No	61	38	3.46(2.14,5.58)	2.47(1.33,4.60)*
**Trimesters of pregnancy**				
First	21	17	1.41(0.69,2.85)	1.01(0.49,5.11)
Second	46	114	0.46(0.29,0.73)	0.81(0.12,3.00)
Third	79	90	----------------	----------------
**Number of pregnancies**				
First time	32	56	---------------	------------------
Second time	56	92	1.06(0.61,1.84)	1.77 (0.51, 7.42)

>_ three times	58	73	1.39(0.79,2.42)	3.53 (0.85, 14.43)
**History of abortion**				
Yes	66	50	2.82(1.79,4.44)	3.33(1.33, 6.05)*
No	80	171	-----------	--------------------
**Awareness about effect of alcohol**				
Having awareness	69	175	-----------	-----------------------
Not having awareness	77	46	4.25(2.68,6.72)	4.66(2.53,8.61)*
**Family social support**				
Have no family social Support	65	58	2.25(1.45,3.51)	2(1.14,3.53)*
Have family social support	81	163	---------------------	----------------

Note. * refers association OR=Odds ratio CI=Confidence Interval

Discussion

The overall prevalence of alcohol consumption among pregnant women was 39.78% with 95% CI (34.74, 44.99), ~~which met the criteria of consuming any amount of alcohol during the current pregnancy period~~. The finding of this study is lower than Ethiopia Demographic and Health Survey (EDHS, 2011), 45% [52]. However, the prevalence of alcohol consumption reported in this study was higher than research conducted in Jimma hospital and Bahir dar dwellers that are 31.3% and

34% respectively [48,53]. The possible reason could be the time of the study; population variation, and study design in particular for Bahirdar study that was community-based study.

This study's prevalence of alcohol consumption was also higher than the magnitude of some of the African countries such as 16% in Uganda [54], 19.6% in South Africa [21], 25% in Uganda [22], 32.5% in Congo [23], 20.4% in Ghana [55] and 22.6% in South Nigeria [38]. Similarly, the magnitude of this study was higher compared to some studies in western countries like the United Kingdom (28.5%), Russia (26.5%) and Switzerland (20.9%) [19]. However, it is in line with a study conducted in Geneva that showed around 36.3% of the women drank at least one glass of alcohol during pregnancy [20]. In contrast to the above, the prevalence of alcohol consumption of this study is far lower compared to the results in Nigeria (59.3%) [26], Ghana (48%) [24], and Australia (56%) [25]. The discrepancy observed might be because of type of alcohol consumed, year of the study, awareness and perception about the effect of alcohol on the fetus, and socio-cultural difference.

The magnitude of binge drinking (drinking four units and more at one occasion), known by the hazardous effect on the fetus, was still significant in this study which is 3.54% with 95% CI (1. 89, 5.98). However, it did not differ from the studies in America (3.1%) [28], Canada (3%) [27], and South Africa (4.6%) [29].Whereas, binge drinking of this study (3.54%) is much lower than studies shown in New Zealand (9%) [30], Uganda (10%), Congo study (20.16%) in 2013 [31], and Congo (25.42%) [23].

Another hazardous pattern of drinking, which is found as a risk factor for abortion and miscarriage according to previous studies, was also determined in this study. Of those pregnant women who consumed alcohol, 4.9% with 95% CI (2.03, 6.65) of them drank four or more unit of alcohol per week. And actually, 11.7% of pregnant women in this study reported as they drank 2 to 3 unit of

alcohol per week which has still an immense negative health effect on fetus evidenced from the previous study. Majority of participants 14% were drinking 2 to 3 units per month. But, a New Zealand study revealed that 10% of pregnant women drank 7 units of alcohol and more within one week which is higher than our study [30]. The discrepancy of the magnitude of binge drinking and amount of alcohol taken per week observed might be due to difference in the culture of society, study design and method of data collection, year of study, and awareness and perception about the effect of alcohol on the fetus, and other socio-demographic characteristics amongst studies.

This study showed that there was a statistically significant association between not having formal education and alcohol consumption which was in line with findings with previous studies [38, 53, 56]. Non-educated women might have no experience in reading and be unaware of the harmful effect of alcohol consumption. But, the opposite finding was observed in another study; it stated that highly educated pregnant women are risky for alcohol consumption [32].

The occupation was also significantly associated with alcohol consumption. Being a housewife was shown as risky for alcohol consumption compared to being employed and/or working their own businesses. This finding is supported by other studies [48, 57]. It could be explained by housewives dependent behaviour on their spouse that could create distress in routine home activity, and their usual engagement in preparing traditional alcohol beverages and ceremonies. Whereas, studies in European countries revealed that employed pregnant women are more likely prone to alcohol consumption [32].

The finding in this study showed that pregnant women lacking awareness about the harmful effect of alcohol on fetus experienced higher levels of alcohol consumption than pregnant women having awareness, this is supported by previous studies [36, 38, 55]. The other variable significantly

associated with alcohol consumption is family social support. Those who have not family social support is predictive of alcohol consumption which is consistent with other studies [33, 34]. But, a study in Boston, Massachusetts revealed that social support is not a factor for alcohol consumption [58].

A woman having unplanned pregnancy and history of abortion was also reported to practice more alcohol consumption which is also in line with the finding of other studies [35–37, 48]. The possible reason could be related to grief about the previous abortion and psychological and physiological effect of abortion which eventually may cause distress and alcohol consumption.

This study didn't show the association between women age, gestational age of participant, gravidity, and ethnicity, number of alive children, religion, average monthly income, previous alcohol use of pregnant women, chat use, and cigarette smoking with alcohol consumption of pregnant women, unlike some previous studies where those variable differences were observed [35,38,59,60].

Even though alcohol consumption is culturally accepted for all socio-demographic, the amount and frequency of alcohol drinking may be under-reported by pregnant women because of the possible perceived stigma and forgetfulness during interviewing. Also, to estimate the average amount of alcohol drank for participants and interviewer during the process of changing the different type of alcohol into the standard unit was difficult to be accurate which also a real challenge and limitation of alcohol and other substance studies across the world.

Conclusions

This finding observed a higher prevalence of alcohol consumption, the significant magnitude of binge drinking, and a serious amount of drinking per week was seen among pregnant women in

Kolfe Keranio sub-city that really requires an effort of prevention and intervention. Factors such as not being educated, being housewife, having unplanned current pregnancy, having a history of abortion, not having awareness about the effect of alcohol on the fetus and not having family social support were associated with alcohol consumption of pregnant women. Guidelines and screening tools have to be developed and integrated into the health care system. Prevention intervention that focuses on creating awareness about the harmful effect of alcohol on pregnancy; strengthening social support, and family planning policy and services that could reduce unplanned pregnancy and abortion ought to have developed and implemented. Moreover, the Longitudinal study that needs daily or weekly follows up with a supporting device that able to measure the amount of alcohol in the blood is necessary for the future researcher.

List of abbreviations

ANC---------------------Ante Natal Care

AOD---------------Alcohol and Other Drugs

CDC--------------Center for Disease Control

FASS-----Fetal Alcohol Spectrum Syndrome

EDHS---Ethiopian Demographic and Health Survey

HC--Health Center

MSPSSf--Multidimensional Scale of Perceived family Social Support

WHO--World Health Organization

Acknowledgements

We would like to acknowledge Addis Ababa University, School of public health for their courage to begin this program and keep it to be continued.

We thank also the staffs, and plan and program officers of Kolfe Keranio sub-city health centers for providing information to determine the sample size of the study, and their permission and supporting to conduct this research. My appreciation also extends to my family and friends for their remarkable support and help in many aspects including finance to complete the study.

References

1. Odejide AO. Status of drug use/abuse in Africa: A review. Int J Ment Health Addict. 2006;4(2):87–102.
2. Organization WH. Atlas on substance use (2010): resources for the prevention and treatment of substance use disorders. Geneva: World Health Organization; 2010.
3. Poznyak V. The impact of alcohol consumption on global health. Tackling Harmful Alcohol Use. 2015;81.
4. McGee CL, Bjorkquist OA, Riley EP, Mattson SN. Impaired language performance in young children with heavy prenatal alcohol exposure. Neurotoxicol Teratol. 2009;31(2):71–5.
5. Kalberg WO, Provost B, Tollison SJ, Tabachnick BG, Robinson LK, Eugene Hoyme H, et al. Comparison of motor delays in young children with fetal alcohol syndrome to those with prenatal alcohol exposure and with no prenatal alcohol exposure. Alcohol Clin Exp Res. 2006;30(12):2037–45.

6. Goldschmidt L, Richardson GA, Cornelius MD, Day NL. Prenatal marijuana and alcohol exposure and academic achievement at age 10. Neurotoxicol Teratol. 2004;26(4):521–32.

7. Linell A, Richardson MX, Wamala S. The Swedish national public health policy report 2010. Scand J Public Health. 2013;41(10_suppl):3–56.

8. Kesmodel U, Wisborg K, Olsen SF, Brink Henriksen T, Jørgen Secher N. Moderate alcohol intake in pregnancy and the risk of spontaneous abortion. Alcohol Alcohol. 2002;37(1):87–92.

9. Harlap S, Shiono PH. Alcohol, smoking, and incidence of spontaneous abortions in the first and second trimester. Lancet. 1980;316(8187):173–6.

10. Rasch V. Cigarette, alcohol, and caffeine consumption: risk factors for spontaneous abortion. Acta Obstet Gynecol Scand. 2003;82(2):182–8.

11. Kesmodel U, Wisborg K, Olsen SF, Henriksen TB, Secher NJ. Moderate alcohol intake during pregnancy and the risk of stillbirth and death in the first year of life. Am J Epidemiol. 2002;155(4):305–12.

12. Nykjaer C, Alwan NA, Greenwood DC, Simpson NAB, Hay AWM, White KLM, et al. Maternal alcohol intake prior to and during pregnancy and risk of adverse birth outcomes: evidence from a British cohort. J Epidemiol Community Heal. 2014;68(6):542–9.

13. Kesmodel U. Binge drinking in pregnancy—frequency and methodology. Am J Epidemiol. 2001;154(8):777–82.

14. Bailey BN, Delaney-Black V, Covington CY, Ager J, Janisse J, Hannigan JH, et al. Prenatal exposure to binge drinking and cognitive and behavioral outcomes at age 7 years. Am J Obstet Gynecol. 2004;191(3):1037–43.

15. Alati R, Al Mamun A, Williams GM, O'Callaghan M, Najman JM, Bor W. In utero alcohol

exposure and prediction of alcohol disorders in early adulthood: a birth cohort study. Arch Gen Psychiatry. 2006;63(9):1009–16.

16. Manning MA, Hoyme HE. Fetal alcohol spectrum disorders: a practical clinical approach to diagnosis. Neurosci Biobehav Rev. 2007;31(2):230–8.

17. Seiler NK. Alcohol and pregnancy: CDC's health advice and the legal rights of pregnant women. Public Health Rep. 2016;131(4):623–7.

18. Butt P, Beirness D, Gliksman L, Paradis C, Stockwell T. Alcohol and health in Canada: a summary of evidence and guidelines for low-risk drinking. Ottawa, Can Cent Subst Abus. 2011;

19. Mårdby A-C, Lupattelli A, Hensing G, Nordeng H. Consumption of alcohol during pregnancy—A multinational European study. Women and Birth. 2017;30(4):e207–13.

20. Dupraz J, Graff V, Barasche J, Etter J-F, Boulvain M. Tobacco and alcohol during pregnancy: prevalence and determinants in Geneva in 2008. Swiss Med Wkly. 2013;143(2122).

21. Petersen Williams P, Jordaan E, Mathews C, Lombard C, Parry CDH. Alcohol and other drug use during pregnancy among women attending midwife obstetric units in the Cape Metropole, South Africa. Adv Prev Med. 2014;2014.

22. Namagembe I, Jackson LW, Zullo MD, Frank SH, Byamugisha JK, Sethi AK. Consumption of alcoholic beverages among pregnant urban Ugandan women. Matern Child Health J. 2010;14(4):492–500.

23. Barthélémy T, Andy M, Roger M. Effect of maternal alcohol consumption on gestational diabetes detection and mother-infant's outcomes in Kinshasa, DR Congo. Open J Obs Gynecol. 2011;1(4):208–12.

24. Lekettey JDP, Dako-Gyeke P, Agyemang SA, Aikins M. Alcohol consumption among pregnant women in James Town Community, Accra, Ghana. Reprod Health. 2017;14(1):120.

25. Alcohol | Australian Government Department of Health [Internet]. [cited 2020 Jun 17]. Available from: https://www.health.gov.au/resources/pregnancy-care-guidelines/part-c-lifestyle-considerations/alcohol

26. Ordinioha B, Brisibe S. Alcohol consumption among pregnant women attending the ante. natal clinic of a tertiary hospital in South. South Nigeria. Niger J Clin Pract. 2015;18(1):13–7.

27. Popova S, Lange S, Probst C, Parunashvili N, Rehm J. Prevalence of alcohol consumption during pregnancy and Fetal Alcohol Spectrum Disorders among the general and Aboriginal populations in Canada and the United States. Eur J Med Genet. 2017;60(1):32–48.

28. Tan CH, Denny CH, Cheal NE, Sniezek JE, Kanny D. Alcohol use and binge drinking among women of childbearing age—United States, 2011–2013. Morb Mortal Wkly Rep. 2015;64(37):1042–6.

29. Popova S, Lange S, Probst C, Shield K, Kraicer- Melamed H, Ferreira- Borges C, et al. Actual and predicted prevalence of alcohol consumption during pregnancy in the WHO African Region. Trop Med Int Heal. 2016;21(10):1209–39.

30. Ho R, Jacquemard R. Maternal alcohol use before and during pregnancy among women in Taranaki, New Zealand. New Zeal Med J. 2009;122(1306).

31. Williams AD, Nkombo Y, Nkodia G, Leonardson G, Burd L. Prenatal alcohol exposure in the Republic of the Congo: prevalence and screening strategies. Birth Defects Res Part A Clin Mol Teratol. 2013;97(7):489–96.

32. Alcohol and Pregnancy: Attitudes Around the Globe [Internet]. [cited 2020 Jun 17]. Available from: https://womensmentalhealth.org/posts/alcohol-pregnancy-attitudes-around-globe/

33. Haynes G, Dunnagan T, Christopher S. Determinants of alcohol use in pregnant women at risk for alcohol consumption. Neurotoxicol Teratol. 2003;25(6):659–66.

34. Barnet B, Duggan AK, Wilson MD, Joffe A. Association between postpartum substance use and depressive symptoms, stress, and social support in adolescent mothers. Pediatrics. 1995;96(4):659–66.

35. Isaksen AB, Østbye T, Mmbaga BT, Daltveit AK. Alcohol consumption among pregnant women in Northern Tanzania 2000–2010: a registry-based study. BMC Pregnancy Childbirth. 2015;15(1):205.

36. Sedgh G, Singh S, Hussain R. Intended and unintended pregnancies worldwide in 2012 and recent trends. Stud Fam Plann. 2014;45(3):301–14.

37. Coleman PK, Reardon DC, Cougle JR. Substance use among pregnant women in the context of previous reproductive loss and desire for current pregnancy. Br J Health Psychol. 2005;10(2):255–68.

38. Onwuka CI, Ugwu EO, Dim CC, Menuba IE, Iloghalu EI, Onwuka CI. Prevalence and predictors of alcohol consumption during pregnancy in South-Eastern Nigeria. J Clin diagnostic Res JCDR. 2016;10(9):QC10.

39. Demaio AR, Dugee O, De Courten M, Bygbjerg IC, Enkhtuya P, Meyrowitsch DW. Exploring knowledge, attitudes, and practices related to alcohol in Mongolia: a national population-based survey. BMC Public Health. 2013;13(1):1–7.

40. Sudhinaraset M, Wigglesworth C, Takeuchi DT. Social and cultural contexts of alcohol use:

Influences in a social–ecological framework. Alcohol Res Curr Rev. 2016;

41. Coles CD, Black MM. Introduction to the special issue: impact of prenatal substance exposure on children's health, development, school performance, and risk behavior. J Pediatr Psychol. 2006;31(1):1–4.
42. Seyoum G, Gebre A. Rapid assessment of the situation of drug and substance abuse in selected urban areas in Ethiopia. Addis Ababa, AAU. 1995;9–55.
43. Wedajo Lemi B. Microbiology of Ethiopian traditionally fermented beverages and condiments. Int J Microbiol. 2020;2020.
44. Narcotic Manual final – EFDA [Internet]. [cited 2020 Jun 18]. Available from: http://www.fmhaca.gov.et/publication/narcotic-manual-final/
45. Abebe H. Isolation and Characterization of the Dominant Yeast in the Traditional beverages of Ethiopia; Tella and Tej. Addis Ababa Universty; 2011.
46. Von Elm E, Altman DG, Egger M, Pocock SJ, Gøtzsche PC, Vandenbroucke JP. The Strengthening the Reporting of Observational Studies in Epidemiology (STROBE) statement: guidelines for reporting observational studies. Ann Intern Med. 2007;147(8):573–7.
47. Kolfe Keranio Sub City - aaca [Internet]. [cited 2020 Jun 15]. Available from: http://www.addisababa.gov.et/de/web/guest/kolfe-keranio-sub-city
48. Anteab K, Demtsu B, Megra M. Assessment of Prevalence and Associated Factors of Alcohol Use during Pregnancy among the dwellers of Bahir-Dar City, Northwest Ethiopia, 2014. 2014;
49. Zimet GD, Dahlem NW, Zimet SG, Farley GK. The multidimensional scale of perceived social support. J Pers Assess. 1988;52(1):30–41.

50. Nakigudde J, Musisi S, Ehnvall A, Airaksinen E, Agren H. Adaptation of the multidimensional scale of perceived social support in a Ugandan setting. Afr Health Sci. 2009;9(2).

51. Saunders JB, Aasland OG, Babor TF, De la Fuente JR, Grant M. Development of the alcohol use disorders identification test (AUDIT): WHO collaborative project on early detection of persons with harmful alcohol consumption- II. Addiction. 1993;88(6):791–804.

52. Statistical Agency C, International I. Ethiopia 2011 Demographic and Health Survey.

53. Mohammed MA, Ahmed JH, Bushra AW, Aljadhey HS. Medications use among pregnant women in Ethiopia: a cross sectional study. J Appl Pharm Sci. 2013;3(4):116.

54. English L, Mugyenyi GR, Ngonzi J, Kiwanuka G, Nightingale I, Koren G, et al. Prevalence of Ethanol Use Among Pregnant Women in Southwestern Uganda. J Obstet Gynaecol Canada JOGC= J d'obstetrique Gynecol du Canada JOGC. 2015;37(10):901–2.

55. Adusi-Poku Y, Edusei AK, Bonney AA, Tagbor H, Nakua E, Otupiri E. Pregnant women and alcohol use in the Bosomtwe district of the Ashanti region-Ghana. Afr J Reprod Health. 2012;16(1).

56. Demographic N. Health Survey 2011. Addis Ababa, Ethiop. 2012;

57. Onah MN, Field S, van Heyningen T, Honikman S. Predictors of alcohol and other drug use among pregnant women in a peri-urban South African setting. Int J Ment Health Syst. 2016;10(1):38.

58. McNAMARA TAYK, Orav EJ, Wilkins-Haug L, Chang G. Social support and prenatal alcohol use. J Women's Heal. 2006;15(1):70–6.

59. Muckle G, Laflamme D, Gagnon J, Boucher O, Jacobson JL, Jacobson SW. Alcohol,

smoking, and drug use among Inuit women of childbearing age during pregnancy and the risk to children. Alcohol Clin Exp Res. 2011;35(6):1081–91.

60. Skeen S, Lund C, Kleintjes S, Flisher A, Consortium MhRP. Meeting the millennium development goals in Sub-saharan Africa: what about mental health? Int Rev Psychiatry. 2010;22(6):624–31.

Supporting information

 Click here to access/download;Figure;Fig 2..tif

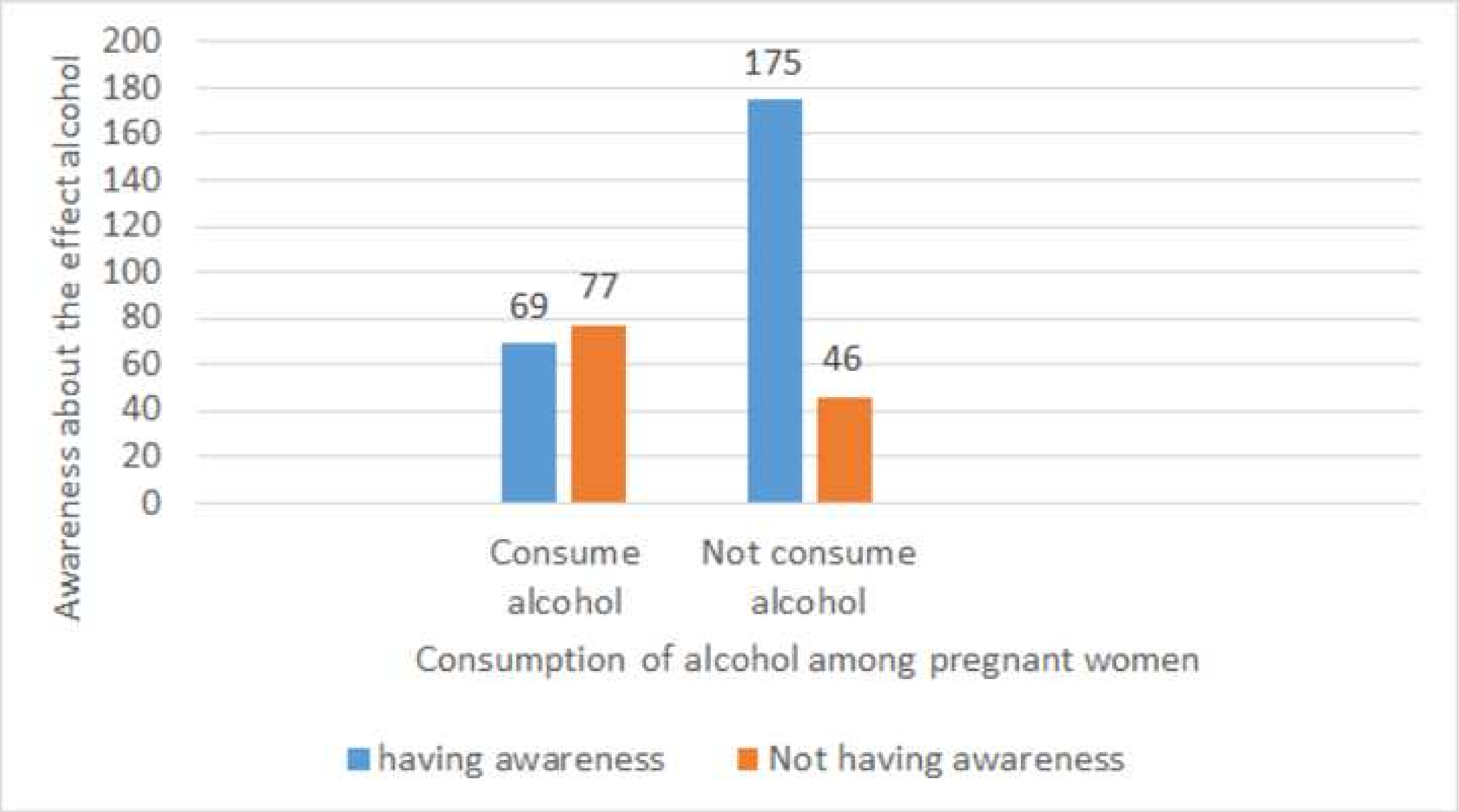

Click here to access/download;Figure;Fig 1..tif

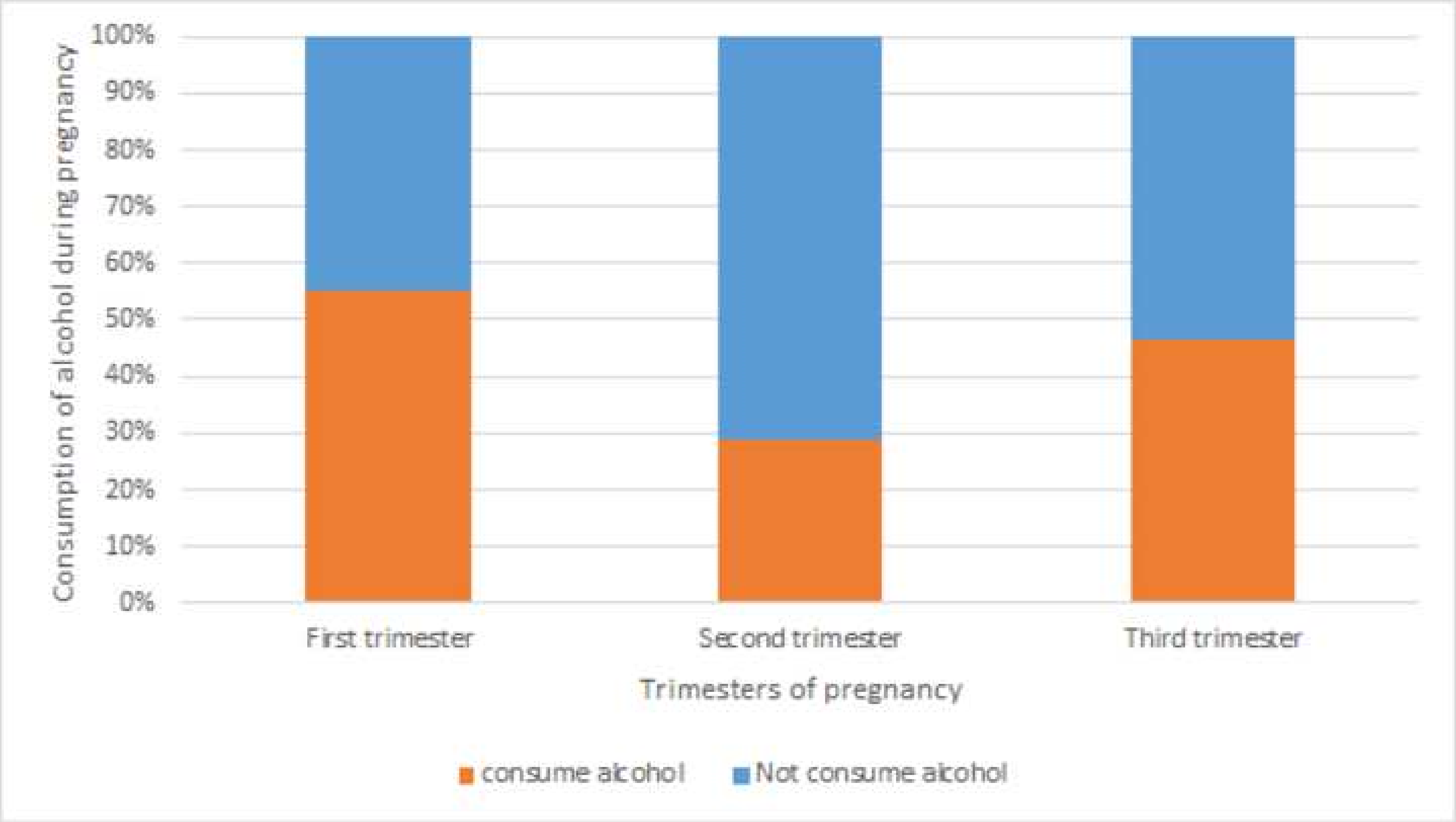

Click here to access/download
Supporting Information
Consumption and binge drinking of alcohol.docx

C~~c~~onsumption of alcohol and binge drinking among pregnant women in health institutions, A~~a~~ddis A~~a~~baba, E~~e~~thiopia: prevalence and determinant factors

Mezinew Sintayehu Bitew[1], Maereg Fekade Zewude[2], Muluken Wubetu[3]

Formatted: Superscript

[1]Department of Mental health, Debre Markos University, Amhara, Ethiopia

[2]~~[1]~~~~Master of Public Health,~~ Department of Maternal and Child Health, Addis Ababa City Administration Health Bureau, Addis Ababa, Ethiopia~~.~~

[3]MSc in Pharmacology, Department of Pharmacy, Debre Markos University, Amhara, Ethiopia.

Corresponding Author

E-mail: sintayehume@gmail.com (MS)

Abstract

~~Background~~Intriduction : People in Ethiopia that includes~~,~~ ~~including~~ pregnant women~~,~~ highly consume both home-made and manufactured alcohol beverages due to of lack of awareness about the harmful effect of risky alcohol use, and cultural acceptance of alcohol consumption. Alcohol consumption and other hazardous patterns of use like binge drinking have tremendous adverse effects on both the fetus and mother. Therefore, this study aimed to assess the magnitude of alcohol

consumption, binge drinking and its determinant factors among pregnant women residing in Kolfe sub-city, Addis Ababa, Ethiopia.

Methods: Institutional based cross-sectional study was conducted on a total of 367 pregnant women once they had been selected by using systematic random sampling. Data was collected using a structured questionnaire. A binary logistic regression model, using SPSS version 20 software, was used to identify associated factors. The results were described using adjusted odds ratio with a 95% confidence interval, and statistical significance was considered at p-value < 0.05.

Results: This study revealed that the prevalence of alcohol consumption, binge drinking, and weekly alcohol consumption of four or more units among pregnant women was 39.78%, 3.54% and 4.9% respectively. Factors ~~like~~ such as not having formal education [AOR 95% CI=8.47(2.42, 29.62), having primary education [AOR 95% CI=4.26(1.23, 14.74), being a housewife[AOR 95% CI= 4.18(2.13,8.22), having an unplanned pregnancy [AOR 95% CI=2.47(1.33, 4.60), having a history of abortion[AOR 95% CI=3.33(1.33, 6.05)], not having awareness about the harmful effect of alcohol consumption [AOR 95% CI=4.66(2.53,8.61)], and not having family social support [AOR 95% CI= 2(1.14,3.53) were found to be significantly associated with alcohol consumption among pregnant women.

Conclusions: This study found that there is a high level of alcohol consumption. Prevention and intervention that focuses on creating awareness about the harmful effects of alcohol, strengthening social support during pregnancy, and family planning policy and services that can reduce unplanned pregnancy and abortion should have developed and implemented.

Keywords: Alcohol, Consumption, Binge, Drinking, Predictors, Pregnancy, Ethiopia

~~Background~~ Introduction

Formatted: Default Paragraph Font, Font: 14 pt

Globally, use of substances such as alcohol, tobacco and other illicit substances, in a way that could harm the users' health, have escalated through time in all segments of the population and has become one of the growing public health and socioeconomic concerns. From these substances, alcohol shares the biggest burden that accounts for 3.8% of all global deaths and 4.6% of the global burden of diseases and neuropsychiatric disorders [1–3]. Risky use of alcohol causes significant morbidity and mortality (particularly from injuries) and societal harm such as social disruption from crime, unemployment and marital disharmony [2, 3].

In pregnant women, the negative health consequences of alcohol consumption are more profound for themselves as well as for the fetus. Alcohol consumption during pregnancy is also showed to be one of the leading causes of preventable birth defects and developmental problems including language and motor delays and poor academic achievement [4–6].

New studies indicate~~d~~ that even low levels of prenatal alcohol exposure could adversely affect the developing fetus. Cognitive and socio-emotional deficits, among children exposed to even small amounts of alcohol, are found [7]. Consumption of <_ 2 or more standard drink of alcohol/day in early pregnancy is associated with significant problems on the fetus that can cause miscarriage ~~abortion~~, preterm birth, low birth weight and low birth centile compared to children born from non-drinkers. In addition, other studies reported that women who consumed more than three drinks per week during the first trimester had a significant negative health effect on pregnancy, and ~~–~~the effect would be five folds more if they took five or more units of alcohol per week [8–12]. Furthermore, binge- drinking (commonly defined as consuming 4 or more units of alcohol at one occasion for women) during pregnancy is more harmful because of the serious toxic effects on neurodevelopment after it crosses the placenta [13, 14].

Adverse effects of prenatal alcohol exposure can range from subtle developmental problems, or fetal alcohol effects, to full-blown fetal alcohol spectrum syndrome (FASS). However, the effects of alcohol exposure are not limited to infancy and childhood; prenatal exposure to alcohol, particularly in early pregnancy, has also been found to increase the likelihood of developing an alcohol disorder in adulthood [15, 16].

Given these consequences and the belief that there is no safe time or amount to consume alcohol in pregnancy, the CDC recommends complete abstinence [17, 18]. ~~As a result of the above-mentioned consequences, the Centers for Disease Control and Prevention (CDC) decided that there is no safe time or safe amount of alcohol during pregnancy, and recommend total abstinence from alcohol consumption during the time of pregnancy (17, 18).~~

~~But, contrary to the recommendation of alcohol abstinence during pregnancy, consumption of alcohol, binge drinking and hazardous use of alcohol during pregnancy is very common and has increased through time across the world.~~ Despite this, ~~S~~studies from various high income and low-income countries have reported a high rate of alcohol consumption by pregnant women from 19.5% in South Africa to 36.3% in Geneva [19-23]. ~~proved this reality~~ The prevalence showed that 26.5%, 28.5%, 36.3%, 19.6%, 25%, and 32.5% in Russia, United Kingdom, South Africa Uganda and Congo respectively [19-23]. ~~(Russia: 26.5%, United Kingdom: 28.5% (19), Geneva: 36.3% (20), South Africa at Cape Town: 19.6% (21), Uganda: 25 % (22), and Congo: 32.5% (23)).~~ The Higher prevalence is also reported from countries like Ghana, Australia, and Nigeria (48%, 56% and 59% respectively) [24–26].

Studies on the prevalence of ~~B~~binge drinking ~~(drinking 4 or more units of alcohol at one occasion/sitting)~~ among pregnant women also reported that ranges from 3% in Canada [27] to 25% far high in Congo [3, 27-31]~~, from a significant magnitude in Canada (3%) (27) to far higher~~

~~prevalence in Congo (25%) (23), were also reported (28–31)~~. The amount of drinking per week, which is the other clinically significant patterns of alcohol use, ~~The other important one, the amount of drinking per week~~ revealed in literature like ~~as an example~~ in New Zealand study which states that~~,~~ 10% of pregnant women drank seven~~7~~ and more units of alcohol per week as well [~~(~~30]~~)~~. Studies from different countries showed that socio-demographic factors (age, marital status, educational status, and occupation), obstetric history (gestational age, unplanned pregnancy, gravidity, and history of abortion), social support and behavioural issues like use of Khat and c~~C~~igarette smoking were predictors for the consumption of alcohol during pregnancy [~~(~~32–37]~~)~~. In addition, studies reported that awareness about the harmful effect of alcohol on fetus, attitude and culture of the population were the determinant factors for alcohol consumption [~~(~~36, 38–40]~~)~~. Because of the rapid increase of alcohol consumption and its terrific associated problems among pregnant women across the world, studies of high-income countries on alcohol and its effect are increased, and the interventions for alcohol problems are also developing [41]. ~~In high-income countries, studies on alcohol and its effects with a solution of the alcohol problems among pregnant women are continued to grow up because of the spread of alcohol epidemics and its terrific effect (41).~~ In low-income countries including Ethiopia, the rapid increase of the consumption of alcohol has also been indicated around two decades ago, and it is still increasing alarmingly and, especially women of fertile age have affected by alcohol [1, 42].~~.~~ ~~As a result, women of fertile age has especially affected by alcohol and chat (1, 42).~~ Both traditional and commercially produced alcohol beverages are widely spread more than ever with the face of lack of awareness about the harmful effect of alcohol, and other predictors of alcohol consumption [1, 38]~~)~~.

People in Ethiopia that include ~~, including~~ pregnant ~~women, highly~~ women highly consume alcohol beverages, especially the culturally accepted traditionally prepared alcohol beverages like Areki, Teje, Tella ~~Areki, Teje, Tella~~ that have a ~~the~~ higher percentage of alcohol amount [~~(~~43–45~~)~~]. Thus, locally made alcohols are produced excessively without control and are usually used in different events like celebrities, ~~different~~ ceremonies, holidays, parties, ~~even in daily family meals,~~ and almost in daily family meals and day to day life ~~in daily bases~~ [~~(~~43–45]~~)~~.

Formatted: Font: Not Italic

Despite the above-mentioned problems, assessment of alcohol consumption with its determinant factors upon pregnant women is limited. In particular, there is a scarcity of study on binge drinking of alcohol by measuring or calculating the amount of alcohol consumed. Addis Ababa, the capital city of Ethiopia where the study is conducted, is heavily affected by hazardous and harmful consumption of alcohol due to both modernization and the deeply rooted cultural acceptance of alcohol consumption [~~(~~1]~~)~~.

~~-~~Therefore, given the above background, this study was intended~~aimed~~ to~~:~~ assess the magnitude of alcohol consumption and binge drinking among pregnant women, and its determinant factors in Kolfe Sub City, Addis Ababa, Ethiopia.

- ~~determine the prevalence of alcohol consumption among pregnant women~~
- ~~assess the magnitude of binge drinking among pregnant women, and~~
- ~~Identify the determinant factors of alcohol consumption among pregnant women in Kolfe sub-city, Addis Ababa, Ethiopia.~~

The finding of this study will have significant contributions for health program ~~managers, policymakers~~managers, policymakers, clinicians and researchers.

Methods

Study design, sample size and sampling technique

An institutional-based quantitative cross-sectional study based on the guidelines of Strengthening the Report of Observational Studies in Epidemiology (STROBE) for observational research [(46]) was conducted at Kkolfe Kkeranio sub-city, Addis Ababa, Ethiopia. Kolfe Kkeranio sub-city, with an area of 61.25 sq.km, has a total population of 546, 219 (235, 360 were female) in 2017. Among the female population, around 2.33 % (9,531) were pregnant. The sub-city has 15 districts and 11 Health Centers (HCs) [(47]).

A total of 397 pregnant mothers were included in a study after calculating by using single population formula (n = $Z_{a/2}^{2}$ z2 * p * (1-p)/d2) by taking proportion (p) of alcohol use of pregnant women (34%) [(48)], 5% margin of error (d) with 95% confidence interval, and by adding 15%, the assumption of non-response rate. Systematic random sampling with ‘k’ value 20 was carried out after samples were proportionally allocated to each of the following 11 health centers: Woreda 1 HC (600*), Woreda 3 HC (1002*), Alem Bank HC (960*), Kolfe HC (846*), Woreda 5 HC (716*), Woreda 6 HC (426*), Keranio HC (798*), Woreda 11 HC (832*), Lomi Meda HC(540*), Woreda 9 HC (470*), and Mikililand HC (880*). Pregnant women aged greater than and equal to 18, those who were in all trimesters, and attended the above-mentioned health centers during the study period were included in the study. Pregnant women who were seriously ill and unable to respond were excluded. The study was conducted from May to June 2017.

Data collection questionnaire/instruments and procedure

Data was collected through a face to face interview by using a structured questionnaire.

Questions regarding socio-demographic, obstetric variables as well as those on alcohol consumption, and awareness about the harmful effect of alcohol and the use of other substances

Formatted: Subscript

Formatted: Superscript

like Khat chewing and cigarette smoking were gathered, adapted and developed from ~~different~~ various literature~~s~~.

Family social support was also assessed by the Multidimensional Scale of Perceived Social Support (MSPSSf). MSPSS has three major subscales (f~~F~~amily, friends and significant others), with a total of 12 items each scaled from 1 (very strongly disagree) to 7 (very strongly agree) [49, 50]. Respondents scored higher than 14 from a multidimensional scale of perceived family social support (MSPSSF) 7 Likert scale were recognized as having family social support, those who scored less than or equal to 14 were not having social support. ~~Then, family subscale was picked and applied to assess family social support (49, 50).~~

Questionnaire regarding the amount and frequency of alcohol consumption per day, week, and month, and generally in the time of pregnancy was adapted from AUDIT and other literature~~s~~ [~~(~~13, 21, and 51]~~)~~. In Ethiopian, both traditional (e.g. Tella, Areki, Teje ~~Tella, Areka and Teje~~) and manufactured (Beer, Wine) alcohol beverages are widely distributed and~~,~~ consumed. ~~and drunk~~ People used ~~using~~ local equipment ~~locally~~ called ~~'~~Tella Birecheko, Melekia, and Birelie to drink traditional alcohols ~~which~~ that can ~~are~~measure about 340 ml, 40 ml and 270 ml volume, respectively. ~~in terms of measurement.~~ Pregnant women were asked to estimate the average number of drinks, and ~~then~~ the data was standardized into a standard unit of alcohol, by using the percentage of alcohol and volume in ml. In this study, alcohol consumption was defined as a pregnant women drank any amount of alcohol during the period of pregnancy. Whereas, drinking four more unit of alcohol on one occasion is considered as binge drinking.

~~-~~Six questions were developed to assess the awareness of the harmful effect of alcohol on fetus, and those who answered the six questions correctly identified as having awareness about the consequences of alcohol.

Formatted: Font: Not Italic

Formatted: Font: Not Italic

Formatted: Font: Not Bold

Furthermore, any amount of khat use and cigarette smoking even at once during the current pregnancy is known as pregnant women who chew khat and smoke cigarette, respectively.

The questionnaire was prepared initially in English and translated to Amharic then back to English to check the consistency of the questions. The training was given to the data collectors and supervisors for three days about the data collection tool, way of interview, the ethical principles of confidentiality and data management before their involvement with data collection.

Eleven BSc nurses with BSc degree qualification as data collectors to collect data and two public health professionals as supervisors to assist and monitor the data collection process were employed. Ethical clearance was obtained from Addis Ababa University, public health school ethical review committee. The right participants were systematically selected by data collectors when pregnant women came to maternal care. Data collectors explained the objective of the study, procedures, and their right to refuse or discontinue the interview at any time to participants. And, they knew that the questionnaire was anonymous and their privacy kept confidential. Also, Participants were aware of as the study has not any risk or direct benefit like incentive for being participant; it was only conducted for the achievement of objectives, and ultimately for improving health services of pregnant women and the community as a whole. After participants heard and understood all the necessary information and consent statement, they have been asked about their willingness to be interviewed, which was verbal consent. Those volunteers were again asked to write their name and put their signature with the date of interview in a prepared blank space found below the information sheet and statement of consent, which was written consent. For participants under 18 years old, informed consent was also obtained from their parents.

After that, data collectors interviewed the selected participant in the face to face manner by using a structured questionnaire at the waiting room of the maternal care clinic that took around 15

Formatted: Left

Formatted: Font: Font color: Custom Color(RGB(14,16,26))

Formatted: Justified, Line spacing: Double

minutes. Those who found risky of alcohol consumption has been linked to substance abuse clinic and, responsible leaders in selected health centers and maternal care clinics were recommended and noticed to initiate and strengthen prevention of alcohol consumption among pregnant women. To control the quality of data, on top of providing training for data collectors and supervisors, supervision was held regularly during the data collection period, and the collected data were checked daily for completeness and consistency. And, Manual and computerized data cleaning were done before data analysis.

Formatted: Font: Font color: Custom Color(RGB(14,16,26))

Operational definitions

Alcohol: a drink containing ethanol. Both home-brewed traditional alcohol (in Ethiopia like Tella, Areka, Teje) and manufactured alcoholic beverages (like Beer, Wine).

Alcohol consumption- defined as, in this study, when pregnant women drank any amount of alcohol during the period of pregnancy.

Binge drinking- pregnant women drank four or more unit of alcohol on one occasion.

Chat use- any amount of chat use during the pregnancy period

Cigarette smoking- history of smoking cigarette during pregnancy period even for once

Having awareness about the effect of alcohol consumption - if respondents answered correctly for all six questions that describe the effect of alcohol consumption on fetus.

Not having awareness about the effect of alcohol consumption - if respondents incorrectly answered for six questions that describe the harmful effects of alcohol use on the fetus.

Not having family social support- if respondents scored less than or equal to 14 from a multidimensional scale of perceived family social support (MSPSSF) 7 Likert scale

Having family social support- if respondents scored greater than 14 from a multidimensional scale of perceived family social support (MSPSSF) 7 liker scale

Data quality control issues

Backward and forward translation of the questionnaire by translators was employed to assure the same meaning is conveyed. The training was given to the data collectors and supervisors about the data collection tool, way of interview, the ethical principles of confidentiality and data management before their involvement with data collection for three days. Supervision was held regularly during the data collection period, and the collected data were checked daily for completeness and consistency. Manual and computerized data cleaning was done before data analysis.

Data processing and analysis

Data were checked, cleaned and entered into Epi.DATA 3.1 versions and then exported into Statistical Package for the Social Sciences (SPSS) window version 20 for analysis. Descriptive summary using frequencies, percentage, graphs, the mean and standard deviation were used to present study results. Binary logistic regression was employed to calculate the adjusted odds ratio (AOR) to identify independent determinant factors of the outcome variable. Variables which met p-value < 0.2 during bivariate analysis were exported to the multivariate analysis model. The findings were described with Odds ratio (crude and adjusted) with corresponding 95% Confidence interval. P-value of < 0.05 was considered as statistically significant during the final multivariable binary logistic regression model.

Ethical approval and consent to participate

Ethical clearance was obtained from Addis Ababa University, public health school ethical review committee. Verbal informed consent was also obtained from participants. They were informed that participation is based on a voluntary basis, and had the full right to withdraw at any time during the interview process. For participants under 18 years old, informed consent was also obtained from their parents.

Results

Description of socio-demographic characteristics

The response rate was 92.44%. Out of 367 pregnant women, 74.7% (274) were married. Two hundred sixty (70.8%) were non-educated, and women who completed secondary education and above were 26 (7.1%). The mean age of respondents was 27.43± SD4.775 years (ranging from 15-49).

In terms of ethnicity, Amhara and Oromo comprise the majority, 114 (31.1%) and 107(29.2%) respectively. Near to half of the participants, 117(48.2%) were followers of orthodox Christian followed by Muslims (35.7%). Majority of pregnant women (42.5%) were housewives while about one-third of them had a monthly average income between 1001-2000ETB (Ttable 1).

Table 1: socio-demographic characteristics of pregnant women in Kolfe Keranio sub city (n=367), Addis Ababa, Ethiopia

Variables	Frequency	Percent (%)
**Age**		
15-19	8	2.2
20-24	100	27.2
25 -29	117	31.9
29-34	106	28.9
>35	36	9.8
Mean (SD)	27.43±4.775	
**Marital status**		
Married	247	67.3
single	81	22.1

Divorce	31	8.4
Widowed	8	2.2
**Ethnicity**		
Amhara	114	31.1
Oromo	107	29.2
Gurage	91	24.8
Tigrie	32	8.7
Others	23	6.3
**Religion**		
Orthodox	177	48.2
Islam	131	35.7
Protestant	40	10.9
Catholic	10	2.7
Others	9	2.5
**Educational status**		
Not educated	127	34.6
Primary educated	123	33.5
Secondary educated	91	24.8
>Secondary educated	26	7.1
**Occupation**		
Employed	105	28.6
Housewife	156	42.5
Own business	106	28.9

**Average monthly income of the house hold**		
≤ 1000 Birr	44	12
1001-2000	109	29.7
2001-3000	76	20.7
3001-4000	61	16.6
>4000	77	21

Assessment of obstetric history of respondents

As shown in **Table 2** below, the majority of the pregnant women 148(40.3%) were ~~are~~ in the second trimester. Almost three quarter (73%) of the pregnant women ~~had a~~have had planned ~~for the current~~ pregnancy. Two hundred fifty-one (68.4%) of pregnant women had no history of abortion ~~**(Table 2)**~~**.**

Table 2: O~~o~~bstetric history of the respondents among pregnant women in kolfe Keranio sub city (n=367) Addis Ababa, Ethiopia

Variables	Frequency	Percent (%)
**Trimester of pregnancy**		
~~< 3 months~~First timester	38	10.4
Second trimester ~~3-6 months~~	148	40.3
Third trimester~~>6 months~~	131	35.7
**Number of pregnancy(gravidity)**		
First time	88	24
Second time	148	40.3
>_ three times	131	35.7

**Number of alive children**		
No child	112	30.5
One	134	36.5
Two	65	17.7
>two	56	15.3
**Planned of current pregnancy**		
Yes	268	73
No	99	27
**History of abortion**		
Yes	116	31.6
No	251	68.4

Though most of the participants were in the second and third trimester, the proportion of alcohol consumption was high among first trimester (55.26%) (Figure 1).

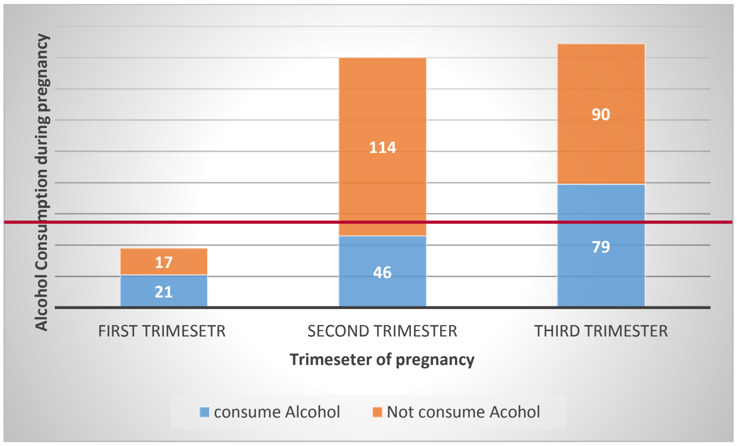

Fig. 1: Alcohol consumption among pregnant women respected to trimesters of pregnancy in Kolfe Keranio sub city (n=367), Addis Ababa, Ethiopia

Alcohol consumption, binge drinking and other substances during pregnancy

As ~~shown~~ illustared in Table 3 below, 146(39.8%) of pregnant women drank alcohol. The majority (44.4%) of participants predominately drank Tella. Fifty-four (14.71%) consumed alcohol 2-4 times per month while 15 (4.09%) of pregnant women drank four and above times per week. On the other hand, 13 (3.54%) of them had the experience of binge drinking during pregnancy ~~(Table 3).~~

Table 3: Alcohol consumption and binge drinking among pregnant women in kolfe sub city (n=367), Addis Ababa, Ethiopia

Variables	Frequency	Percent (%)
**Alcohol consumption before pregnancy**		
Yes	179	48.8
No	188	51.2
**Alcohol consumption during pregnancy**		
Yes	146	39.8
No	221	60.2
**Binge drinking during pregnancy**		
Yes	13	3.54
No	354	96. 46
**Frequency of alcohol consumption during pregnancy**		
<_ once a month	36	9.81
2-4 times per month	54	14.71
2-3times per week	41	11.17

4 and above per week	15	4.09
**Type of alcohol use**		
Tel<u>l</u>a	65	44.4
Teje	9	6.3
Arek<u>i</u>~~a~~	14	9.7
Beer	41	28.5
Wine	9	6.3
Others	7	4.9
**Cigarette smoking during pregnancy**		
Yes	10	2.7
No	357	97.3
**<u>K</u>~~C~~hat use during pregnancy**		
Yes	39	10.6
No	328	89.4

Awareness about the harmful effect of alcohol use

As ~~illustrated~~ <u>demonstrated</u> in <u>F</u>~~f~~ig~~ure~~ 2, Two hundred forty-four (66.5%) pregnant women have awareness, answered all six questions correctly regarding the harmful effect of alcohol consumption. Among those who have awareness, only 69 (28.28%) consumed alcohol. Whereas, of those who answered the awareness questions incorrectly, 76 (62.6%) drank alcohol<u>.</u> ~~(Figure 2).~~

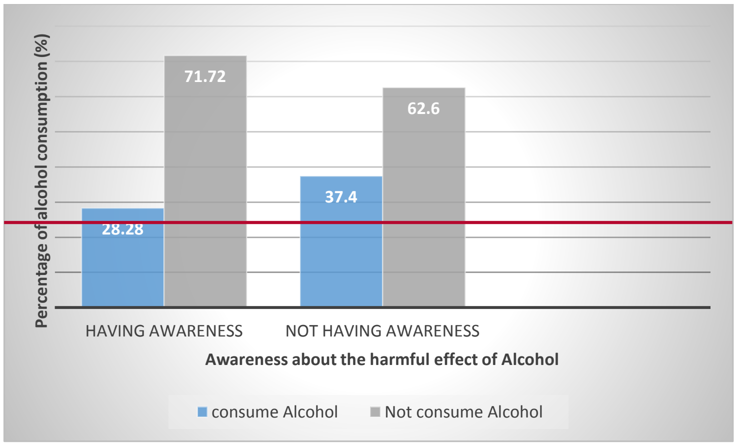

Fig 2: Magnitude of alcohol consumption among pregnant women respected to awareness about the harmful effect of alcohol in Kolfe Keranio sub city (n=367), Addis Ababa, Ethiopia

~~Assessment of family social support of respondents~~

~~In the assessment of family social support, more than a half (66.5%) scored greater than or equal to 14 from the total score of MSPSSf.~~

Factors associated with alcohol consumption among pregnant women attending Kolfe Sub-city health centers

As shown in **T~~t~~able 4**, educational status, occupation, plan of pregnancy, history of abortion, awareness about the harmful effect of alcohol, and family social support of pregnant women were significant predictors of alcohol consumption among pregnant women with p-value<0.05. Statistically, significant higher alcohol consumption rate was observed among non-educated pregnant women and those who found in primary education compared to pregnant women who completed secondary education and above with (AOR 95%CI= 8.47(2.42, 29.62) and AOR 95%CI= 4.26(1.23, 14.74) respectively.

Pregnant women who were in some form of occupation appeared to have a decreased risk of alcohol consumption compared to those who were housewife that had a fourfold risk of alcohol consumption with (AOR 95% CI=4.18(2.13, 8.22). Respondents who have had unplanned current pregnancy had a twofold prone to alcohol consumption than who had planned pregnancy (AOR 95% CI=2.47(1.33, 4.60).

Those pregnant women that had a history of abortion experienced three-fold risk for alcohol consumption compared to pregnant women who hadn't a history of abortion with [AOR 95% CI=3.33(1.33, 6.05)]. Women who answered correctly about the effect of alcohol on fetus and them also found to be a protective factor for alcohol consumption compared to those who answered incorrectly which become a risk factor [AOR 95% CI 4.66 (2.53, 8.61)]. Moreover, in assessment family social support, more than a half (66.5%) scored greater than or equal to 14 from the total score of MSPSSf. And the study found a relationship between family social support and alcohol consumption; pregnant women who hadn't family social support were two-fold risks for alcohol consumption [AOR 95% CI= 2(1.14,3.53) compared to those who had family social support. ~~(Table 4).~~

Table 4: Factors associated alcohol use of pregnant women in Kolfe Keranio sub city (n=367), Addis Ababa, Ethiopia

	Alcohol consumption during pregnancy			
**Variables**	**Yes**	**No**	**Crude OR (95% CI)**	**Adjusted OR (95% CI)**
**Religion**				
Orthodox	76	101	-----------------	------------------------
Islam	43	88	0.65 (0.41,1.04)	1.25(042,2.13)

Protestant	24	16	1.99(0.99,4.01)	3.01(0.96,5.02)
Catholic	2	8	0.33(0.69,1.61)	0.23(0.11, 1.71)
Others	1	8	0.166 (0.02,1.360	7.09 (0.68, 18.4)
**Educational status**				
Not educated	65	62	4.40(1.56,12.40)	8.47(2.42,29.62)*
Primary education	61	62	4.13(1.47,11.66)	4.26(1.23,14.74)*
Secondary education	15	76	0.83(0.27,2.54)	1.41(0.38,5.23)
>secondary education	5	21	-----------------	-------------------------
**Occupation**				
Employed	34	71	-------------------	-----------------
housewife	87	69	2.63(1.57,4.41)	4.18(2.13,8.22)*
own business	25	81	0.65(0.35,1.18)	0.76(0.35,1.66)
**Plan of pregnancy**				
Yes	85	183	------------------	------------------
No	61	38	3.46(2.14,5.58)	2.47(1.33,4.60)*
**Trimesters of pregnancy**				
First	21	17	1.41(0.69,2.85)	1.01(0.49,5.11)
Second	46	114	0.46(0.29,0.73)	0.81(0.12,3.00)
Third	79	90	----------------	----------------
**Number of pregnancies**				
First time	32	56	---------------	------------------

Second time	56	92	1.06(0.61,1.84)	1.77 (0.51, 7.42)
>_ three times	58	73	1.39(0.79,2.42)	3.53 (0.85, 14.43)
**History of abortion**				
Yes	66	50	2.82(1.79,4.44)	3.33(1.33, 6.05)*
No	80	171	-----------	--------------------
**Awareness about effect of alcohol**				
Having awareness	69	175	-----------	-----------------------
Not having awareness	77	46	4.25(2.68,6.72)	4.66(2.53,8.61)*
**Family social support**				
Have no family social Support	65	58	2.25(1.45,3.51)	2(1.14,3.53)*
Have family social support	81	163	---------------------	----------------

Note. * refers association   OR=Odds ratio   CI=Confidence Interval

Discussion

The overall prevalence of alcohol consumption among pregnant women was 39.78% with 95% CI (34.74, 44.99), which met the criteria of consuming any amount of alcohol during the current pregnancy period. The finding of this study is lower than Ethiopia Demographic and Health Survey (EDHS, 2011), 45% [(52)]. However, the prevalence of alcohol consumption reported in this study was higher than research conducted in Jimma hospital and Bahir dar dwellers that are 31.3% and

34% respectively [~~(~~48,53]~~)~~. The possible reason could be the time of the study; population variation, and study design in particular for Bahirdar study that was community-based study.

This study's prevalence of alcohol consumption was also higher than the magnitude of some of the African countries such as 16% in Uganda [54], 19.6% in South Africa [21], 25% in Uganda [22], 32.5% in Congo [23], 20.4% in Ghana [55] and 22.6% in South Nigeria [38]. ~~the following some African countries: in Southwestern Uganda (16%) in 2015 (54), South Africa at Cape Town (19.6%) (21), Uganda study (25%) in 2010 (22), Congo (32.5%) (23), Ghanaian pregnant women (20.4%) (55), and south-east Nigeria (22.6%) in 2016 (38).~~ Similarly, the magnitude of this study was higher compared to some studies in western countries like the United Kingdom (28.5%), Russia (26.5%) and Switzerland (20.9%) [~~(~~19]~~)~~. However, it is in line with a study conducted in Geneva that showed around 36.3% of the women drank at least one glass of alcohol during pregnancy [~~(~~20]~~)~~. In contrast to the above, the prevalence of alcohol consumption of this study is far lower compared to the studies in Nigeria (59.3%) [~~(~~26]~~)~~, Ghana (48%) [~~(~~24~~)~~], and Australia (56%) [~~(~~25]~~)~~. The discrepancy observed might be because of type of alcohol consumed, year of the study, awareness and perception about the effect of alcohol on the fetus, and socio-cultural difference.

The magnitude of binge drinking (drinking four units and more at one occasion), known by the hazardous effect on the fetus, was still significant in this study which is 3.54% with 95% CI (1. 89, 5.98). However, it did not differ from the studies in America (3.1%) [~~(~~28~~)~~], Canada (3%) [~~(~~27]~~)~~, and South Africa (4.6%) [~~(~~29~~)~~]. Whereas, binge drinking of this study (3.54%) is much lower than studies shown in New Zealand (9%) [~~(~~30~~)~~], Uganda (10%), Congo study (20.16%) in 2013 [~~(~~31]~~)~~, and Congo (25.42%) [~~(~~23]~~)~~.

Another hazardous pattern of drinking, which is found as a risk factor for abortion and miscarriage according to previous studies, was also determined in this study. Of those pregnant women who consumed alcohol, 4.9% with 95% CI (2.03, 6.65) of them drank four or more unit of alcohol per week. And actually, 11.7% of pregnant women in this study reported as they drank 2 to 3 unit of alcohol per week which has still an immense negative health effect on fetus evidenced from the previous study. Majority of participants 14% were drinking 2 to 3 units per month. But, a New Zealand study revealed that 10% of pregnant women drank 7 units of alcohol and more within one week which is higher than our study [(30]). The discrepancy of the magnitude of binge drinking and amount of alcohol taken per week observed might be due to difference in the culture of society, study design and method of data collection, year of study, and awareness and perception about the effect of alcohol on the fetus, and other socio-demographic characteristics amongst studies.

This study showed that there was a statistically significant association between not having formal education and alcohol consumption which was in line with findings with previous studies [(38, 53, 56]). Non-educated women might have no experience in reading and be unaware of the harmful effect of alcohol consumption. But, the opposite finding was observed in another study; it stated that highly educated pregnant women are risky for alcohol consumption [(32]).

The occupation was also significantly associated with alcohol consumption. Being a housewife was shown as risky for alcohol consumption compared to being employed and/or working their own businesses. This finding is supported by other studies [(48, 57)]. It could be explained by housewives dependent behaviour on their spouse that could create distress in routine home activity, and their usual engagement in preparing traditional alcohol beverages and ceremonies. Whereas, studies in European countries revealed that employed pregnant women are more likely prone to alcohol consumption [(32]).

The finding in this study showed that pregnant women lacking awareness about the harmful effect of alcohol on fetus experienced higher levels of alcohol consumption than pregnant women having awareness, this is supported by previous studies [~~(~~36, 38, 55]~~)~~. The other variable significantly associated with alcohol consumption is family social support. Those who have not family social support is predictive of alcohol consumption which is consistent with other studies [~~(~~33, 34]~~)~~. But, a study in Boston, Massachusetts revealed that social support is not a factor for alcohol consumption [~~(~~58]~~)~~.

A woman having unplanned pregnancy and history of abortion was also reported to practice more alcohol consumption which is also in line with the finding of other studies [~~(~~35–37, 48]~~)~~. The possible reason could be related to grief about the previous abortion and psychological and physiological effect of abortion which eventually may cause distress and alcohol consumption.

This study didn't show the association between women age, gestational age of participant, gravidity, and ethnicity, number of alive children, religion, average monthly income, previous alcohol use of pregnant women, chat use, and cigarette smoking with alcohol consumption of pregnant women, unlike some previous studies where those variable differences were observed [~~(~~35,38,59,60~~)~~].

~~Limitation of the study~~

Even though alcohol consumption is culturally accepted for all socio-demographic, the amount and frequency of alcohol drinking may be under-reported ~~might not truly be told~~ by pregnant women because of the possible perceived stigma and forgetfulness during ~~and not correctly remembered as well during~~ interviewing. In addition, to estimate the average amount of alcohol drank for participants and interviewer during the process of changing the different type of alcohol

into the standard unit was difficult to be accurate which also a real challenge and limitation of alcohol and other substance studies across the world.

Conclusions ~~and recommendations~~

Formatted: Font: 16 pt

This finding observed a higher prevalence of alcohol consumption, the significant magnitude of binge drinking, and a serious amount of drinking per week was seen among pregnant women in Kolfe Keranio sub-city that really requires an effort of prevention and intervention. Factors such as not being educated, being housewife, having unplanned current pregnancy, having a history of abortion, not having awareness about the effect of alcohol on the fetus and not having family social support were associated with alcohol consumption of pregnant women. Guidelines and screening tools have to be developed and integrated into the health care system. Prevention intervention that focuses on creating awareness about the harmful effect of alcohol on pregnancy; strengthening social support, and family planning policy and services that could reduce unplanned pregnancy and abortion ought to have developed and implemented. Moreover, the Longitudinal study that needs daily or weekly follows up with a supporting device that able to measure the amount of alcohol in the blood is necessary for the future researcher.

List of abbreviations

Formatted: Font: 18 pt

ANC---------------------Ante Natal Care

AOD---------------Alcohol and Other Drugs

CDC--------------Center for Disease Control

FASS-----Fetal Alcohol Spectrum Syndrome

EDHS---Ethiopian Demographic and Health Survey

HC--Health Center

MSPSSf--Multidimensional Scale of Perceived family Social Support

WHO--World Health Organization

~~**Declarations**~~

~~**Ethical approval and consent to participate**~~

~~Ethical clearance was obtained from Addis Ababa university public health school ethical review committee. Both written and verbal informed consent was obtained from participants. They were informed that participation is based on voluntary bases and have full right to withdraw at any time during the interview process. And actually, written informed consent was also got from their parents for participants under 18 years old. Moreover, the researcher was striving to protect and to respect the privacy, secrecy and wellbeing of persons with these conditions. The data's which is collected for the purpose of this study not contained identifying information, thus ensuring the secrecy of the participants. All the data collected used for the purpose of this study only.~~

~~**Consent for publication**~~

~~Not applicable~~

~~**Availability of data and materials**~~

~~The datasets used and analyzed in this study are available from the corresponding author upon reasonable request.~~

~~**Competing interest**~~

~~The authors declare that they have no competing interests~~

~~**Funding**~~

~~There was no specific fund received for this research study.~~

~~**Authors' contribution**~~

~~1. FM. Conceived of the study, and performed its design, statistical analysis, interpretation, helped to draft the manuscript~~

~~2. SM. Participated in conceived of the study, in development of design, statistical analysis, and interpretation of the study and helped to draft the manuscript~~

~~3. WM. Participated in development of questionnaire, statistical analysis, interpretation of the study and helped to draft the manuscript~~

Acknowledgements

We would like to acknowledge Addis Ababa University, School of public health for their courage to begin this program and keep it to be continued.

We thank also the staffs, and plan and program officers of Kolfe Keranio sub-city health centers for providing information to determine the sample size of the study, and their permission and supporting to conduct this research. My appreciation also extends to my family and friends for their remarkable support and help in many aspects including finance to complete the study.

Formatted: Font: 18 pt

Formatted: Heading 1, Left, Line spacing: single

~~**Authors' information**~~

~~**Fekade M. Zewde** (BSC in public health, MPH in general public health, work as a senior public health officer, clinician, Addis Ababa City Administration Health Bureau, Ethiopia)~~

~~**Sintayehu M. Bitew** (BSc in nursing, MSc in integrated clinical and community mental health, lecturer in college of medicine and health science, debremarkos university, Ethiopia)~~

~~**Muluken Wubetu** MSc in Pharmacology, Department of Pharmacy, Ass. Prof. in college of medicine and health science, Debre Markos University, Ethiopia)~~

References

1. Odejide AO. Status of drug use/abuse in Africa: A review. Int J Ment Health Addict. 2006;4(2):87–102.
2. Organization WH. Atlas on substance use (2010): resources for the prevention and treatment of substance use disorders. Geneva: World Health Organization; 2010.
3. Poznyak V. The impact of alcohol consumption on global health. Tackling Harmful Alcohol Use. 2015;81.
4. McGee CL, Bjorkquist OA, Riley EP, Mattson SN. Impaired language performance in young children with heavy prenatal alcohol exposure. Neurotoxicol Teratol. 2009;31(2):71–5.
5. Kalberg WO, Provost B, Tollison SJ, Tabachnick BG, Robinson LK, Eugene Hoyme H, et al. Comparison of motor delays in young children with fetal alcohol syndrome to those with prenatal alcohol exposure and with no prenatal alcohol exposure. Alcohol Clin Exp Res. 2006;30(12):2037–45.
6. Goldschmidt L, Richardson GA, Cornelius MD, Day NL. Prenatal marijuana and alcohol exposure and academic achievement at age 10. Neurotoxicol Teratol. 2004;26(4):521–32.
7. Linell A, Richardson MX, Wamala S. The Swedish national public health policy report 2010. Scand J Public Health. 2013;41(10_suppl):3–56.
8. Kesmodel U, Wisborg K, Olsen SF, Brink Henriksen T, Jørgen Secher N. Moderate alcohol intake in pregnancy and the risk of spontaneous abortion. Alcohol Alcohol. 2002;37(1):87–92.
9. Harlap S, Shiono PH. Alcohol, smoking, and incidence of spontaneous abortions in the first and second trimester. Lancet. 1980;316(8187):173–6.
10. Rasch V. Cigarette, alcohol, and caffeine consumption: risk factors for spontaneous

abortion. Acta Obstet Gynecol Scand. 2003;82(2):182–8.

11. Kesmodel U, Wisborg K, Olsen SF, Henriksen TB, Secher NJ. Moderate alcohol intake during pregnancy and the risk of stillbirth and death in the first year of life. Am J Epidemiol. 2002;155(4):305–12.

12. Nykjaer C, Alwan NA, Greenwood DC, Simpson NAB, Hay AWM, White KLM, et al. Maternal alcohol intake prior to and during pregnancy and risk of adverse birth outcomes: evidence from a British cohort. J Epidemiol Community Heal. 2014;68(6):542–9.

13. Kesmodel U. Binge drinking in pregnancy—frequency and methodology. Am J Epidemiol. 2001;154(8):777–82.

14. Bailey BN, Delaney-Black V, Covington CY, Ager J, Janisse J, Hannigan JH, et al. Prenatal exposure to binge drinking and cognitive and behavioral outcomes at age 7 years. Am J Obstet Gynecol. 2004;191(3):1037–43.

15. Alati R, Al Mamun A, Williams GM, O'Callaghan M, Najman JM, Bor W. In utero alcohol exposure and prediction of alcohol disorders in early adulthood: a birth cohort study. Arch Gen Psychiatry. 2006;63(9):1009–16.

16. Manning MA, Hoyme HE. Fetal alcohol spectrum disorders: a practical clinical approach to diagnosis. Neurosci Biobehav Rev. 2007;31(2):230–8.

17. Seiler NK. Alcohol and pregnancy: CDC's health advice and the legal rights of pregnant women. Public Health Rep. 2016;131(4):623–7.

18. Butt P, Beirness D, Gliksman L, Paradis C, Stockwell T. Alcohol and health in Canada: a summary of evidence and guidelines for low-risk drinking. Ottawa, Can Cent Subst Abus. 2011;

19. Mårdby A-C, Lupattelli A, Hensing G, Nordeng H. Consumption of alcohol during

pregnancy—A multinational European study. Women and Birth. 2017;30(4):e207–13.

20. Dupraz J, Graff V, Barasche J, Etter J-F, Boulvain M. Tobacco and alcohol during pregnancy: prevalence and determinants in Geneva in 2008. Swiss Med Wkly. 2013;143(2122).

21. Petersen Williams P, Jordaan E, Mathews C, Lombard C, Parry CDH. Alcohol and other drug use during pregnancy among women attending midwife obstetric units in the Cape Metropole, South Africa. Adv Prev Med. 2014;2014.

22. Namagembe I, Jackson LW, Zullo MD, Frank SH, Byamugisha JK, Sethi AK. Consumption of alcoholic beverages among pregnant urban Ugandan women. Matern Child Health J. 2010;14(4):492–500.

23. Barthélémy T, Andy M, Roger M. Effect of maternal alcohol consumption on gestational diabetes detection and mother-infant's outcomes in Kinshasa, DR Congo. Open J Obs Gynecol. 2011;1(4):208–12.

24. Lekettey JDP, Dako-Gyeke P, Agyemang SA, Aikins M. Alcohol consumption among pregnant women in James Town Community, Accra, Ghana. Reprod Health. 2017;14(1):120.

25. Alcohol | Australian Government Department of Health [Internet]. [cited 2020 Jun 17]. Available from: https://www.health.gov.au/resources/pregnancy-care-guidelines/part-c-lifestyle-considerations/alcohol

26. Ordinioha B, Brisibe S. Alcohol consumption among pregnant women attending the ante. natal clinic of a tertiary hospital in South. South Nigeria. Niger J Clin Pract. 2015;18(1):13–7.

27. Popova S, Lange S, Probst C, Parunashvili N, Rehm J. Prevalence of alcohol consumption

during pregnancy and Fetal Alcohol Spectrum Disorders among the general and Aboriginal populations in Canada and the United States. Eur J Med Genet. 2017;60(1):32–48.

28. Tan CH, Denny CH, Cheal NE, Sniezek JE, Kanny D. Alcohol use and binge drinking among women of childbearing age—United States, 2011–2013. Morb Mortal Wkly Rep. 2015;64(37):1042–6.

29. Popova S, Lange S, Probst C, Shield K, Kraicer- Melamed H, Ferreira- Borges C, et al. Actual and predicted prevalence of alcohol consumption during pregnancy in the WHO African Region. Trop Med Int Heal. 2016;21(10):1209–39.

30. Ho R, Jacquemard R. Maternal alcohol use before and during pregnancy among women in Taranaki, New Zealand. New Zeal Med J. 2009;122(1306).

31. Williams AD, Nkombo Y, Nkodia G, Leonardson G, Burd L. Prenatal alcohol exposure in the Republic of the Congo: prevalence and screening strategies. Birth Defects Res Part A Clin Mol Teratol. 2013;97(7):489–96.

32. Alcohol and Pregnancy: Attitudes Around the Globe [Internet]. [cited 2020 Jun 17]. Available from: https://womensmentalhealth.org/posts/alcohol-pregnancy-attitudes-around-globe/

33. Haynes G, Dunnagan T, Christopher S. Determinants of alcohol use in pregnant women at risk for alcohol consumption. Neurotoxicol Teratol. 2003;25(6):659–66.

34. Barnet B, Duggan AK, Wilson MD, Joffe A. Association between postpartum substance use and depressive symptoms, stress, and social support in adolescent mothers. Pediatrics. 1995;96(4):659–66.

35. Isaksen AB, Østbye T, Mmbaga BT, Daltveit AK. Alcohol consumption among pregnant women in Northern Tanzania 2000–2010: a registry-based study. BMC Pregnancy

Childbirth. 2015;15(1):205.
36. Sedgh G, Singh S, Hussain R. Intended and unintended pregnancies worldwide in 2012 and recent trends. Stud Fam Plann. 2014;45(3):301–14.
37. Coleman PK, Reardon DC, Cougle JR. Substance use among pregnant women in the context of previous reproductive loss and desire for current pregnancy. Br J Health Psychol. 2005;10(2):255–68.
38. Onwuka CI, Ugwu EO, Dim CC, Menuba IE, Iloghalu EI, Onwuka CI. Prevalence and predictors of alcohol consumption during pregnancy in South-Eastern Nigeria. J Clin diagnostic Res JCDR. 2016;10(9):QC10.
39. Demaio AR, Dugee O, De Courten M, Bygbjerg IC, Enkhtuya P, Meyrowitsch DW. Exploring knowledge, attitudes, and practices related to alcohol in Mongolia: a national population-based survey. BMC Public Health. 2013;13(1):1–7.
40. Sudhinaraset M, Wigglesworth C, Takeuchi DT. Social and cultural contexts of alcohol use: Influences in a social–ecological framework. Alcohol Res Curr Rev. 2016;
41. Coles CD, Black MM. Introduction to the special issue: impact of prenatal substance exposure on children’s health, development, school performance, and risk behavior. J Pediatr Psychol. 2006;31(1):1–4.
42. Seyoum G, Gebre A. Rapid assessment of the situation of drug and substance abuse in selected urban areas in Ethiopia. Addis Ababa, AAU. 1995;9–55.
43. Wedajo Lemi B. Microbiology of Ethiopian traditionally fermented beverages and condiments. Int J Microbiol. 2020;2020.
44. Narcotic Manual final – EFDA [Internet]. [cited 2020 Jun 18]. Available from: http://www.fmhaca.gov.et/publication/narcotic-manual-final/

45. Abebe H. Isolation and Characterization of the Dominant Yeast in the Traditional beverages of Ethiopia; Tella and Tej. Addis Ababa Universty; 2011.

46. Von Elm E, Altman DG, Egger M, Pocock SJ, Gøtzsche PC, Vandenbroucke JP. The Strengthening the Reporting of Observational Studies in Epidemiology (STROBE) statement: guidelines for reporting observational studies. Ann Intern Med. 2007;147(8):573–7.

47. Kolfe Keranio Sub City - aaca [Internet]. [cited 2020 Jun 15]. Available from: http://www.addisababa.gov.et/de/web/guest/kolfe-keranio-sub-city

48. Anteab K, Demtsu B, Megra M. Assessment of Prevalence and Associated Factors of Alcohol Use during Pregnancy among the dwellers of Bahir-Dar City, Northwest Ethiopia, 2014. 2014;

49. Zimet GD, Dahlem NW, Zimet SG, Farley GK. The multidimensional scale of perceived social support. J Pers Assess. 1988;52(1):30–41.

50. Nakigudde J, Musisi S, Ehnvall A, Airaksinen E, Agren H. Adaptation of the multidimensional scale of perceived social support in a Ugandan setting. Afr Health Sci. 2009;9(2).

51. Saunders JB, Aasland OG, Babor TF, De la Fuente JR, Grant M. Development of the alcohol use disorders identification test (AUDIT): WHO collaborative project on early detection of persons with harmful alcohol consumption- II. Addiction. 1993;88(6):791–804.

52. Statistical Agency C, International I. Ethiopia 2011 Demographic and Health Survey.

53. Mohammed MA, Ahmed JH, Bushra AW, Aljadhey HS. Medications use among pregnant women in Ethiopia: a cross sectional study. J Appl Pharm Sci. 2013;3(4):116.

54. English L, Mugyenyi GR, Ngonzi J, Kiwanuka G, Nightingale I, Koren G, et al. Prevalence of Ethanol Use Among Pregnant Women in Southwestern Uganda. J Obstet Gynaecol Canada JOGC= J d'obstetrique Gynecol du Canada JOGC. 2015;37(10):901–2.

55. Adusi-Poku Y, Edusei AK, Bonney AA, Tagbor H, Nakua E, Otupiri E. Pregnant women and alcohol use in the Bosomtwe district of the Ashanti region-Ghana. Afr J Reprod Health. 2012;16(1).

56. Demographic N. Health Survey 2011. Addis Ababa, Ethiop. 2012;

57. Onah MN, Field S, van Heyningen T, Honikman S. Predictors of alcohol and other drug use among pregnant women in a peri-urban South African setting. Int J Ment Health Syst. 2016;10(1):38.

58. McNAMARA TAYK, Orav EJ, Wilkins-Haug L, Chang G. Social support and prenatal alcohol use. J Women's Heal. 2006;15(1):70–6.

59. Muckle G, Laflamme D, Gagnon J, Boucher O, Jacobson JL, Jacobson SW. Alcohol, smoking, and drug use among Inuit women of childbearing age during pregnancy and the risk to children. Alcohol Clin Exp Res. 2011;35(6):1081–91.

60. Skeen S, Lund C, Kleintjes S, Flisher A, Consortium MhRP. Meeting the millennium development goals in Sub-saharan Africa: what about mental health? Int Rev Psychiatry. 2010;22(6):624–31.

Supporting information

A copy of marked manuscript that shows revision

Formatted: Font: (Default) +Headings (Calibri Light), 18 pt, Font color: Auto

Formatted: Heading 1, Line spacing: single

Thank you so much all of you, editors and the two reviewers, for reviewing the manuscript critically for the sake of publishing quality scientific research.

Response to academic editor's comment and questions

1. Please ensure that your manuscript meets PLOS ONE's style requirements, including those for file naming. The PLOS ONE style templates can be found

   Answer: I made the manuscript in line with the requirements of PlOS ONE
2. For a question -During our internal evaluation we noted that participants provided verbal consent, did the ethics committees/IRBs approve this consent procedure?

   Answer: in the actual procedure of the work, a copy of the master thesis, mother document of this manuscript, it states that both verbal and written informed consent was obtained. Data collectors explained to participants about the objective of the study, procedures, and their right to refuse or discontinue the interview at any time and other important information. After participants heard and understood all the necessary information and consent statement, they have been asked about their willingness to be interviewed, which is verbal consent. Those volunteers were again asked to write their name and put their signature with the date of interview at a prepared blank space found below the information sheet and statement of consent, which is written consent. That means they provide both verbal and written informed consent. So, in the manuscript that states "only verbal consent" was an error and misunderstanding when it was written in research ethics subheading. Now, I accepted your comments and took a correction in this revised manuscript.
3. Contradictory of the message regarding pregnant women under age 18 in the section of exclusion criteria and ethical consideration.

   Answer: Yes, pregnant women start from 15 years old were enrolled in the study. Therefore, I removed the message that excludes women under 18 years old from the section of exclusion criteria.
4. In the abstract it is stated that the study "was conducted from April to June 2017", but in the methods section of the manuscript it is stated that the study "was conducted from May to June 2017".

Answer: the right period of data collection was from May to June 2017. I amended it.

5. Requested to update ORCID ID.

   Answer: I did it.

Response for Reviewers

Reviewer 1

1. Are there national survey data (prevalence rates) for alcohol/drug use in the general population that can be included in the Background section, to provide some context for why this is a public health concern in Ethiopia?

   Answer: There are only local single articles in a different part of the country, and all reports that it the public health problems like the other countries across the world. But, there is no national survey study.
2. Strong comments in method section regarding how data collected, how women approached, how the research described, and about the destination of pregnant women who used alcohol in a risky way

   Answer: I accept all the comments and incorporate the raised concerns. Data collectors explained the objective and procedures of the study, and their right to refuse or discontinue the interview at any time to participants. Participants were aware of as the study has not any risk or direct benefit like incentives for them; it was only conducted for the achievement of objectives, and ultimately for improving health services of pregnant women and the community as a whole. After that, data collectors interviewed the selected participant in the face to face manner by using a structured questionnaire at the waiting room of the maternal care clinic that took around 15 minutes. Those who found risky of alcohol consumption has been linked to substance abuse clinic, and responsible leaders in selected health centers and maternal care clinics were recommended and noticed to initiate and strengthen prevention of alcohol consumption among pregnant women.
3. A question regarding missing data?

   Answer: From the beginning, we have been adding 10% of possible non-response participants on the calculated samples. The non-responses in a manuscript were

refusals to participate but not missing data's/values. Fortunately, the actual non-response rate was below the prior plan or expected non-response rate that couldn't affect the analysis and conclusion.

4. A comment of adding stigma or any other thing that could hinder the disclosure of alcohol use.
   Answer: I understand your concern, and I try to add some possible perceived stigma. But, as I mentioned in the background of the study, there is a cultural acceptance of alcohol use in all demographic population. Since alcohol is historical and frequently used in our culture, which may be associated with Orthodox Christianity religion, stigma and punishment are not found.

Reviewer 2

Answer: I saw all questions, comment and suggestion on each page of the document or manuscript that you gave me. I thank you for all you did on a manuscript. And I found it is essential for me and scientific research as well. I accepted all the comments and suggested solutions that you wrote and marked, and I corrected it in a document accordingly. I also received a note that you told me to incorporate the independent sub-heading variable (social support) with the next subheading in table four (associated factors- subheadings). But, I didn't do for the variable of awareness about the harmful effect of alcohol consumption because this has a figure that describes and presents a descriptive message different from table four (associated factors). And I minimized the method sub-headings and merged it.
