# Supplementary material for: Consumption of alcohol and binge drinking among pregnant women in Addis Ababa, Ethiopia: Prevalence and determinant factors
Source: PLoS One. 2020 Dec 22;15(12):e0243784. doi: 10.1371/journal.pone.0243784 (PMC7755214; doi:10.1371/journal.pone.0243784)
Supplement: Attachment — Submitted filename: PONE-D-20-19931_R2_reviewer PPW 20201105.pdf [file pone.0243784.s005.pdf]

PLOS ONE

Consumption of alcohol and binge drinking among pregnant women in Addis Ababa, Ethiopia: prevalence and determinant factors

--Manuscript Draft--

**Manuscript Number:**	PONE-D-20-19931R2
**Article Type:**	Research Article
**Full Title:**	Consumption of alcohol and binge drinking among pregnant women in Addis Ababa, Ethiopia: prevalence and determinant factors
**Short Title:**	Consumption of alcohol and binge drinking among pregnant women in Addis Ababa, Ethiopia
**Corresponding Author:**	Mezinew Sintayehu Bitew<br>Debre Markos University<br>Debre Markos, ETHIOPIA
**Keywords:**	Alcohol, Consumption, Binge, Drinking, Determinants, Pregnancy, Ethiopia
**Abstract:**	Introduction: People in Ethiopia, including pregnant women, highly consume both home-made and manufactured alcohol beverages due to lack of awareness about the harmful effect of risky alcohol use, and cultural acceptance of alcohol consumption. Alcohol consumption and other hazardous patterns of use like binge drinking have tremendous adverse effects on fetus and mothers. Therefore, this study aimed to assess the magnitude of alcohol consumption, binge drinking and its determinants among pregnant women residing in Kolfe sub-city, Addis Ababa, Ethiopia.<br><br>Methods: Institutional based cross-sectional study was conducted among a total of 367 pregnant women. The participants were selected using a systematic random sampling method. Data were collected through a structured questionnaire. A binary logistic regression was conducted using SPSS version 20 software to identify determinants of alcohol consumption and binge drinking. A p-value < 0.05 was used to declare a statistical significance in multiple logistic regression. The results were described using adjusted odds ratio with a 95% confidence interval.<br><br>Results: This study revealed that the prevalence of alcohol consumption, binge drinking, and weekly alcohol consumption of four or more units among pregnant women was 39.78%, 3.54% and 4.9%, respectively. Not having formal education [AOR 95% CI=8.47 (2.42, 29.62), having primary education [AOR 95% CI=4.26 (1.23, 14.74), being a housewife [AOR 95% CI= 4.18 (2.13, 8.22), having an unplanned pregnancy [AOR 95% CI=2.47(1.33, 4.60), having a history of abortion [AOR 95% CI=3.33 (1.33, 6.05)], not having awareness about the harmful effect of alcohol consumption [AOR 95% CI=4.66 (2.53, 8.61)], and not having family social support [AOR 95% CI= 2(1.14,3.53) were determinants of alcohol consumption among pregnant women.<br><br>Conclusions: This study found a high level of alcohol consumption among pregnant women. Interventions to create awareness on the harmful effects of alcohol are needed. Moreover, strengthening social support during pregnancy and family planning services to reduce unplanned pregnancy and abortion should be considered.
**Order of Authors:**	Mezinew Sintayehu Bitew
	Maereg Fekade Zewde
	Muluken Wubetu
	Addisu Alehegn Alemu
**Response to Reviewers:**	Response to reviewers<br>First, we thank you for the editors and reviewers!<br>For both reviewer<br>1.We modified all the texts of a document and tried to present intelligently and write in a standard English<br>For reviewer 2<br>We corrected all the comments and suggestions line by line you provide

Powered by Editorial Manager® and ProduXion Manager® from Aries Systems Corporation

	1.Line 30-typing error<br>2.Line 52 and 53- grammar error<br>3.Line 64-verb problem<br>4.Line 69-symbol problem<br>5.Line 87 to 93- comments to remove and correct<br>6.102 to104- ambiguous sentence<br>7.Line 107 and 108-<br>8.Line 113<br>9.Line 222<br>10.Line 228 and 229- it has not a problem; it is the title of figure 1 which should be placed below the paragraph of that message according to the guideline of PLoS one<br>11.Line 272 and 273- commented to remove<br><br>Thank you again!
Additional Information:	
Question	Response
**Financial Disclosure**<br><br>Enter a financial disclosure statement that describes the sources of funding for the work included in this submission. Review the submission guidelines for detailed requirements. View published research articles from *PLOS ONE* for specific examples.<br><br>This statement is required for submission and **will appear in the published article** if the submission is accepted. Please make sure it is accurate.<br><br>**Unfunded studies**<br>Enter: *The author(s) received no specific funding for this work.*<br><br>**Funded studies**<br>Enter a statement with the following details:<br>• Initials of the authors who received each award<br>• Grant numbers awarded to each author<br>• The full name of each funder<br>• URL of each funder website<br>• Did the sponsors or funders play any role in the study design, data collection and analysis, decision to publish, or preparation of the manuscript?<br>• **NO** - Include this sentence at the end of your statement: *The funders had no role in study design, data collection and analysis, decision to publish, or preparation of the manuscript.*<br>• **YES** - Specify the role(s) played.	The authors received no specific funding for this work.

* typeset	
**Competing Interests**<br><br>Use the instructions below to enter a competing interest statement for this submission. On behalf of all authors, disclose any competing interests that could be perceived to bias this work—acknowledging all financial support and any other relevant financial or non-financial competing interests.<br><br>This statement **will appear in the published article** if the submission is accepted. Please make sure it is accurate. View published research articles from *PLOS ONE* for specific examples.<br><br>**NO authors have competing interests**<br><br>Enter: *The authors have declared that no competing interests exist.*<br><br>**Authors with competing interests**<br><br>Enter competing interest details beginning with this statement:<br><br>*I have read the journal's policy and the authors of this manuscript have the following competing interests: [insert competing interests here]*<br><br>* typeset	The authors have declared that no competing interests exist.
**Ethics Statement**<br><br>Enter an ethics statement for this submission. This statement is required if the study involved:<br><br>• Human participants<br>• Human specimens or tissue<br>• Vertebrate animals or cephalopods<br>• Vertebrate embryos or tissues<br>• Field research<br><br>Write "N/A" if the submission does not require an ethics statement.	Ethical clearance was obtained from Addis Ababa University, public health school ethical review committee. Both Verbal and written informed consent were also obtained from participants. They were informed that participation is based on a voluntary basis, and had the full right to withdraw at any time during the interview process. For participants under 18 years old, informed consent was also obtained from their parents.

Powered by Editorial Manager® and ProduXion Manager® from Aries Systems Corporation

General guidance is provided below. Consult the submission guidelines for detailed instructions. **Make sure that all information entered here is included in the Methods section of the manuscript.**<br><br>**Format for specific study types**<br><br>**Human Subject Research (involving human participants and/or tissue)**<br>• Give the name of the institutional review board or ethics committee that approved the study<br>• Include the approval number and/or a statement indicating approval of this research<br>• Indicate the form of consent obtained (written/oral) or the reason that consent was not obtained (e.g. the data were analyzed anonymously)<br><br>**Animal Research (involving vertebrate animals, embryos or tissues)**<br>• Provide the name of the Institutional Animal Care and Use Committee (IACUC) or other relevant ethics board that reviewed the study protocol, and indicate whether they approved this research or granted a formal waiver of ethical approval<br>• Include an approval number if one was obtained<br>• If the study involved *non-human primates*, add *additional details* about animal welfare and steps taken to ameliorate suffering<br>• If anesthesia, euthanasia, or any kind of animal sacrifice is part of the study, include briefly which substances and/or methods were applied<br><br>**Field Research**<br><br>Include the following details if this study involves the collection of plant, animal, or other materials from a natural setting:<br>• Field permit number<br>• Name of the institution or relevant body that granted permission	
**Data Availability**<br><br>Authors are required to make all data underlying the findings described fully	Yes - all data are fully available without restriction

available, without restriction, and from the time of publication. PLOS allows rare exceptions to address legal and ethical concerns. See the PLOS Data Policy and FAQ for detailed information.<br><br>A Data Availability Statement describing where the data can be found is required at submission. Your answers to this question constitute the Data Availability Statement and **will be published in the article**, if accepted.<br><br>**Important:** Stating 'data available on request from the author' is not sufficient. If your data are only available upon request, select 'No' for the first question and explain your exceptional situation in the text box.<br><br>Do the authors confirm that all data underlying the findings described in their manuscript are fully available without restriction?	
**Describe where the data may be found in full sentences. If you are copying our sample text, replace any instances of XXX with the appropriate details.**<br><br>• If the data are **held or will be held in a public repository**, include URLs, accession numbers or DOIs. If this information will only be available after acceptance, indicate this by ticking the box below. For example: *All XXX files are available from the XXX database (accession number(s) XXX, XXX.*).<br>• If the data are all contained **within the manuscript and/or Supporting Information files**, enter the following: *All relevant data are within the manuscript and its Supporting Information files.*<br>• If neither of these applies but you are able to provide **details of access elsewhere**, with or without limitations, please do so. For example:<br><br>*Data cannot be shared publicly because of [XXX]. Data are available from the XXX Institutional Data Access / Ethics*	All relevant data are within the manuscript and its Supporting Information files.

Committee (contact via XXX) for researchers who meet the criteria for access to confidential data.<br><br>*The data underlying the results presented in the study are available from (include the name of the third party and contact information or URL).*<br>• This text is appropriate if the data are owned by a third party and authors do not have permission to share the data.<br><br>* typeset	
Additional data availability information:	

Powered by Editorial Manager® and ProduXion Manager® from Aries Systems Corporation

Manuscript Click here to access/download;Manuscript;Manuscript.docx

Consumption of alcohol and binge drinking among pregnant women in Addis Ababa, Ethiopia: prevalence and determinant factors

Mezinew Sintayehu Bitew[1*], Maereg Fekade Zewde[2], Muluken Wubetu[3], Addisu Alehegn Alemu[4]

[1]Department of mental health, Debre Markos University, Debre Markos, Amhara, Ethiopia

[2] Department of Maternal and Child Health, Addis Ababa City Administration Health Bureau, Addis Ababa, Ethiopia

[3]Department of Pharmacy, Debre Markos University, Debre Markos, Amhara, Ethiopia.

[4]Department of midwifery, Debre Markos University, Debre Markos, Amhara, Ethiopia.

* Corresponding Author

E-mail: sintayehume@gmail.com (MS)

Abstract

Introduction: People in Ethiopia, including pregnant women, highly consume both home-made and manufactured alcohol beverages due to lack of awareness about the harmful effect of risky alcohol use, and cultural acceptance of alcohol consumption. Alcohol consumption and other hazardous patterns of use like binge drinking have tremendous adverse effects on fetus and mothers. Therefore, this study aimed to assess the magnitude of alcohol consumption, binge drinking and its determinants among pregnant women residing in Kolfe sub-city, Addis Ababa, Ethiopia.

Methods: Institutional based cross-sectional study was conducted among a total of 367 pregnant women. The participants were selected using a systematic random sampling method. Data were collected through a structured questionnaire. A binary logistic regression was conducted using SPSS version 20 software to identify determinants of alcohol consumption and binge drinking. A p-value < 0.05 was used to declare a statistical significance in multiple logistic regression. The results were described using adjusted odds ratio with a 95% confidence interval.

Results: This study revealed that the prevalence of alcohol consumption, binge drinking, and weekly alcohol consumption of four or more units among pregnant women was 39.78%, 3.54% and 4.9%, respectively. Not having formal education [AOR 95% CI=8.47 (2.42, 29.62), having primary education [AOR 95% CI=4.26 (1.23, 14.74), being a housewife [AOR 95% CI= 4.18 (2.13, 8.22), having an unplanned pregnancy [AOR 95% CI=2.47(1.33, 4.60), having a history of abortion [AOR 95% CI=3.33 (1.33, 6.05)], not having awareness about the harmful effect of alcohol consumption [AOR 95% CI=4.66 (2.53, 8.61)], and not having family social support [AOR 95% CI= 2(1.14,3.53) were determinants of alcohol consumption among pregnant women.

Conclusions: This study found a high level of alcohol consumption among pregnant women. Interventions to create awareness on the harmful effects of alcohol are needed. Moreover, strengthening social support during pregnancy and family planning services to reduce unplanned pregnancy and abortion should be considered.

Keywords: Alcohol, Consumption, Binge, Drinking, Determinants, Pregnancy, Ethiopia

Introduction

Globally, use of substances such as alcohol, tobacco and other illicit substances, in a way that could harm the users' health, have escalated through time in all segments of the population and has become one of the growing public health and socioeconomic concerns. Alcohol shares the highest-burden that accounts for 3.8% of all global deaths and 4.6% of the global burden of diseases and neuropsychiatric disorders [1–3]. Risky use of alcohol causes significant morbidity and mortality (particularly from injuries) and societal harm such as social disruption from crime, unemployment and marital disharmony [2, 3].

The negative health consequences of alcohol consumption are more profound for pregnant women and their fetuses. Alcohol consumption during pregnancy has also shown to be one of the leading causes of preventable birth defects and developmental problems comprising language and motor delays and deprived academic achievement [4–6].

New studies indicate that even low levels of prenatal alcohol exposure could adversely affect the developing fetus. Cognitive and socio-emotional deficits, among children exposed to even small amounts of alcohol, are found [7]. Consumption of 2 or more standard drink of alcohol/day in early pregnancy is associated with significant problems on the fetus. It can cause miscarriage, preterm birth and low birth weight compared to children born from non-drinkers. In addition, other studies

reported that women who consumed more than three drinks per week during the first trimester had a significant harmful health effect on their pregnancy. The consequence would be five folds more if they took five or more units of alcohol per week [8–12]. Furthermore, binge- drinking (commonly defined as consuming 4 or more units of alcohol at one occasion for women) during pregnancy is more harmful because of its serious toxic effects on fetal neurodevelopment crossing the placenta [13, 14].

The adverse effects of prenatal alcohol exposure can range from subtle developmental problems, or fetal alcohol effects, to full-blown fetal alcohol spectrum syndrome (FASS). However, it is not limited to infancy and childhood; prenatal exposure to alcohol, particularly in early pregnancy, has also been found to increase the likelihood of developing an alcohol disorder in adulthood [15, 16]. Given these consequences and the belief that there is no safe time or amount to consume alcohol in pregnancy, the CDC recommends complete abstinence [17, 18].

Different studies have reported alcohol consumption among pregnant women ranges from 19.5% in South Africa and 59 % in Nigeria [19-26].

Similarly, studies conducted on the prevalence of binge drinking among pregnant women showed between 3% in Canada and 25% in Congo [3, 27-31]. A study in New Zealand also revealed 10% of pregnant women drank seven and more units of alcohol per week which is the other clinically significant patterns of alcohol use [30].

According to studies from different countries showed that socio-demographic factors (age, marital status, educational status, and occupation), obstetric history (gestational age, unplanned pregnancy, gravidity, and history of abortion), social support and behavioral issues like use of Khat and cigarette smoking were determinants of alcohol consumption during pregnancy [32–37].

Additionally, studies reported that awareness about the harmful effect of alcohol on fetus, attitude and culture of the population were determinants of alcohol consumption [36, 38–40].

Alcohol consumption and its associated terri[illegible]problems among pregnant women increased globally [41]. In low-income countries including Ethiopia, the rapid increase of the consumption of alcohol has also been indicated for decades, especially reproductive-age women are affected [1, 42]. Both traditional and commercially produced alcohol beverages are widely spread more than ever which are also an important contributing factors for increased alcohol consumption in Ethiopia [1, 38].

People in Ethiopia that include pregnant women highly consume alcohol beverages, especially the traditionally prepared alcohol beverages like Areki, Teje, Tella that have higher percentages of alcohol amount [43–45]. Thus, locally made alcohols are being used excessively without control in different holidays, religious festivals, parties, and almost in daily family meals and day to day life [43–45].

Despite the adverse health effects of alcohol consumption is profound, its assessment with determinants among pregnant women is limited. In particular, there is a scarcity of information on binge drinking of alcohol by measuring or calculating the amount consumed. Addis Ababa, the capital city of Ethiopia where the current study was conducted, is heavily affected with consumption of alcohol [1].Therefore, this study was intended to assess the magnitude of alcohol consumption and binge drinking among pregnant women, and its determinants in Kolfe sub city, Addis Ababa, Ethiopia. The finding of this study will have significant contributions for health program managers, policymakers, clinicians and researchers.

Materials and Methods

Study design, sample size and sampling technique

An institutional-based quantitative cross-sectional study was conducted based on the guidelines of strengthening the Report of Observational Studies in Epidemiology (STROBE) for observational research [46]. It was conducted at Kolfe Keranio sub-city, Addis Ababa, Ethiopia. The sub-city covers an area of 61.25 sq.km and has a total population of 546, 219 (235, 360 females). Around 2.33 % (9,531) of females were pregnant. The sub-city has 15 districts and 11 Health Centers (HCs) [47].

A total of 397 pregnant women were included in this study. Single population proportion formula was considered to calculate the sample size. Taking 34% prevalence (p) of alcohol consumption among pregnant women [48], 5% margin of error (d), 95% confidence interval (CI) and 15% non-response rate. Systematic random sampling was carried out after the sample was proportionally allocated to each of the 11 health centers. All pregnant women, regardless of the gestational age, attended the health centers during the study period were included. However, pregnant women who were seriously ill and unable to respond were excluded. The study was conducted from May to June 2017.

Data collection instrument and procedure

Data regarding on participants' socio-demographic, obstetric characteristics, alcohol consumption, awareness about the harmful effect of alcohol and the use of other substances (i.e. Khat chewing and cigarette smoking) were collected using a structured questionnaire developed from previous literature.

However, family social support was assessed through the Multidimensional Scale of Perceived Social Support (MSPSSf). MSPSS has three subscales (family, friends and significant others), with a total of 12 items each scaled from 1 (very strongly disagree) to 7 (very strongly agree) [49, 50]. Respondents scored higher than 14 from a multidimensional scale of perceived family social support (MSPSSF) 7 Likert scale were recognized as having family social support, those who scored less than or equal to 14 were not having social support.

Likewise, the amount and frequency of alcohol consumption per day, week and month during the time of pregnancy were adapted from AUDIT and other articles [13, 21, and 51]. In Ethiopian, both traditional (e.g. Tella, Araki, Teje) and manufactured (Beer, Wine) alcohol beverages are widely produced and consumed. People used local equipment's called Tella Birecheko, Melekia, and Birelie to drink traditional alcohols that can measure around 340 ml, 40 ml and 270 ml, respectively. Pregnant women were asked to estimate the average number of drinks they consumed. Finally, the data were standardized using the percentage and volume of alcohol in each ml of beverages. And drinking four more units of alcohol on one occasion was considered binge drinking.

Six questions were developed to assess the awareness of the harmful effect of alcohol on fetus, and those who answered the six questions correctly identified as having awareness about the consequences of alcohol. Furthermore, any amount of khat use and cigarette smoking even at once during the current pregnancy is known as pregnant women who chew khat and smoke cigarette, respectively. The questionnaire was prepared initially in English and translated to Amharic then back to English to check the consistency of the questions.

.

Eleven BSc nurses as a data collector and two public health officers as supervisor participated during data collection. Ethical clearance was obtained from Addis Ababa University, public health school ethical review committee. The right participants were systematically selected by data collectors when pregnant women came to maternal care. Data collectors explained the objective and procedures of the study, and their right to refuse or discontinue the interview at any time to participants. And, they knew that the questionnaire was anonymous and their privacy kept confidential. Also, Participants were aware of as the study has not any risk or direct benefit like incentives for them; it was only conducted for the achievement of objectives, and ultimately for improving health services of pregnant women and the community as a whole. After participants heard and understood all the necessary information and consent statement, they have been asked about their willingness to be interviewed, which was verbal consent. Those volunteers were again asked to write their name and put their signature with the date of interview in a prepared blank space found below the information sheet and statement of consent, which was written consent. For participants under 18 years old, informed consent was also obtained from their parents.

After that, data collectors interviewed the selected participant in the face to face manner by using a structured questionnaire at the waiting room of the maternal care clinic that took around 15 minutes. Those who found risky of alcohol consumption has been linked to substance abuse clinic, and responsible leaders in selected health centers and maternal care clinics were recommended and noticed to initiate and strengthen prevention of alcohol consumption among pregnant women.

Data quality, processing and analysis

To assure the data quality training was given to the data collectors and supervisors for three days about the data collection tool, way of interview, and the ethical principles of confidentiality before their involvement to the data collection. The collected data were checked for its completeness and

consistency daily. Then, the collected data were checked, cleaned and entered into Epi. DATA 3.1 version and exported to Statistical Package for the Social Sciences (SPSS) version 20 for analysis. Descriptive statistics were summarized using frequencies, percentage, graphs, the mean and standard deviation. Binary logistic regression was employed to assess the association of each independent variables with the outcome variable. All variables with p-value < 0.2 during binary logistic regression were considered in the multiple logistic regression. The findings were presented using Odds ratio (crude and adjusted) with its 95%CIs. Moreover, a p-value < 0.05 was considered to declare the variable was statistically significant during the final multiple logistic regression analysis.

Results and discussion

Results

Description of socio-demographic characteristics

A total of 367 pregnant women were interviewed that made 92.44% response rate. This study revealed almost three fourth (74.7%) of participants were married. Seven out of ten (70.8%) participants were non-educated, whereas women who completed secondary education and above were 26 (7.1%). The age of participants ranged between 15 and 49 with mean age 27.43 (SD ± 4.77) years. Regarding ethnicity, Amhara and Oromo comprise the majority, 114 (31.1%) and 107(29.2%) respectively. Nearly half of the participants (48.2%) were followers of orthodox religion followed by Muslims (35.7%). The majority (42.5%) of pregnant women were housewives. Similarly, one-third of participants' monthly average income was between1001-2000 ETB (**Table 1)**.

Table 1: Socio-demographic characteristics of pregnant women in Kolfe Keranio sub city (n=367), Addis Ababa, Ethiopia

Variables	Frequency	Percent
**Age**		
15-19	8	2.2
20-24	100	27.2
25 -29	117	31.9
30-34	106	28.9
≥35	36	9.8
Mean (SD)	27.43±4.775	
**Marital status**		
Married	247	67.3
Single	81	22.1
Divorced	31	8.4
Widowed	8	2.2
**Ethnicity**		
Amhara	114	31.1
Oromo	107	29.2
Gurage	91	24.8
Tigrie	32	8.7
Others	23	6.3
**Religion**		

Orthodox	177	48.2
Muslim	131	35.7
Protestant	40	10.9
Catholic	10	2.7
Others	9	2.5
**Educational status**		
Not educated	127	34.6
Primary education	123	33.5
Secondary education	91	24.8
Above secondary education	26	7.1
**Occupation**		
Employed	105	28.6
Housewife	156	42.5
Own business	106	28.9
**Average monthly house hold income**		
≤ 1000 Birr	44	12
1001-2000	109	29.7
2001-3000	76	20.7
3001-4000	61	16.6
>4000	77	21

Assessment of obstetric history of respondents

As shown in **Table 2** below, the majority of pregnant women (40.3%) were in the second trimester. Almost three fourth (73%) of pregnant women had a planned pregnancy. Three or more out of ten (31.6%) of pregnant women had a history of abortion**.**

Table 2: Obstetric history of the pregnant women in kolfe Keranio sub city (n=367) Addis Ababa, Ethiopia

Variables	Frequency	Percent
**Trimester of pregnancy**		
First trimester	38	10.4
Second trimester	148	40.3
Third trimester	131	35.7
**Number of pregnancy(gravidity)**		
One	88	24
Two	148	40.3
≥ Three	131	35.7
**Number of alive children**		
0One	112	30.5
Two	134	36.5
> Two	65	17.7
	56	15.3
**Planned pregnancy**		
Yes	268	73
No	99	27

**History of abortion**		
Yes	116	31.6
No	251	68.4

Though most of the participants were in the second and third trimester, the proportion of alcohol consumption value was high among first trimester 21 (55.26%) **(Fig 1)**.

Fig. 1: Alcohol consumption among pregnant women respected to trimesters of pregnancy in Kolfe Keranio sub city (n=367), Addis Ababa, Ethiopia

Alcohol consumption, binge drinking and other substances during pregnancy

As illustrated in **(Table 3)** below, 146(39.8%) of pregnant women drank alcohol. The majority (44.4%) of participants predominately drank Tella. Fifty-four (14.71%) of participants consumed alcohol 2-4 times per month, while 15 (4.09%) of pregnant women drank four and above times per week. On the other hand, 13 (3.54%) of women had the experience of binge drinking during their pregnancy.

Table 3: Alcohol consumption and binge drinking among pregnant women in kolfe sub city (n=367), Addis Ababa, Ethiopia

**Variables**	**Frequency**	**Percent)**
**Alcohol consumption before pregnancy**		
Yes	179	48.8
No	188	51.2
**Alcohol consumption during pregnancy**		
Yes	146	39.8
No	221	60.2

**Binge drinking during pregnancy**		
Yes	13	3.54
No	354	96. 46
**Frequency of alcohol consumption during pregnancy**		
≤ once a month	36	9.81
2-4 times per month	54	14.71
2-3times per week	41	11.17
≥ 4 times per week	15	4.09
**Type of alcohol use**		
Tella	65	44.4
Teje	9	6.3
Areki	14	9.7
Beer	41	28.5
Wine	9	6.3
Others	7	4.9
**Cigarette smoking during pregnancy**		
Yes	10	2.7
No	357	97.3
**Khat use during pregnancy**		
Yes	39	10.6
No	328	89.4

Awareness about the harmful effect of alcohol use

As demonstrated in **Fig 2**, Two hundred forty-four (66.5%) pregnant women have awareness, answered all six questions correctly regarding the harmful effect of alcohol consumption. Among those who have awareness, only 69 (28.28%) consumed alcohol. Whereas, of those who answered the awareness questions incorrectly, 76 (62.6%) drank alcohol.

Fig 2: Magnitude of alcohol consumption among pregnant women respected to awareness about the harmful effect of alcohol on fetus in Kolfe Keranio sub city (n=367), Addis Ababa, Ethiopia

Factors associated with alcohol consumption among pregnant women

As shown in **Table 4**, educational status, occupation, plan of pregnancy, history of abortion, awareness about the harmful effect of alcohol, and family social support of pregnant women were significant predictors of alcohol consumption among pregnant women. The higher odds alcohol consumption was observed among non-educated and attained primary education pregnant women with (AOR 95% CI= 8.47 (2.42, 29.62) and AOR 95% CI= 4.26 (1.23, 14.74), respectively compared to those completed secondary education and above.

Pregnant women who were housewives had a fourfold risk of alcohol consumption (AOR 95% CI=4.18 (2.13, 8.22) compared to employed women. Women whose last pregnancy was unplanned had a two folds risk of alcohol consumption than their counters (AOR 95% CI=2.47(1.33, 4.60). Likewise, pregnant women who had a history of abortion were prone to three-fold risk for alcohol consumption compared to pregnant women who hadn’t [AOR 95% CI=3.33 (1.33, 6.05)]. Women who had awareness about the effect of alcohol on fetus and them found to be a protective factor for alcohol consumption compared to those who had no awareness [AOR 95% CI 4.66 (2.53, 8.61)]. Moreover, in assessment family social support, more than half (66.5%) of them scored

greater than or equal to 14 from the total score of MSPSSf. This study also found pregnant women who hadn't family social support had a two-fold risk for alcohol consumption [AOR 95% CI= 2 (1.14, 3.53) compared to those who had family social support.

Table 4: Determinants of alcohol consumption among pregnant women in Kolfe Keranio sub city Addis Ababa, Ethiopia (n=367)

	Alcohol consumption during pregnancy			
**Variables**	**Yes**	**No**	**COR (95% CI)**	**A OR (95% CI)**
**Religion**				
Orthodox	76	101	1.00	1.00
Islam	43	88	0.65 (0.41, 1.04)	1.25 (042, 2.13)
Protestant	24	16	1.99 (0.99, 4.01)	3.01 (0.96, 5.02)
Catholic	2	8	0.33 (0.69, 1.61)	0.23 (0.11, 1.71)
Others	1	8	0.166 (0.02, 1.36)	7.09 (0.68, 18.4)
**Educational status**				
Not educated	65	62	4.40 (1.56, 12.40)	8.47 (2.42, 29.62)*
Primary education	61	62	4.13 (1.47, 11.66)	4.26 (1.23, 14.74)*
Secondary education	15	76	0.83 (0.27, 2.54)	1.41 (0.38, 5.23)

Above secondary education	5	21	1.00	1.00
**Occupation**				
Employed	34	71	1.00	1.00
housewife	87	69	2.63 (1.57, 4.41)	4.18 (2.13, 8.22)*
own business	25	81	0.65 (0.35, 1.18)	0.76 (0.35, 1.66)
**Plan of pregnancy**				
Yes	85	183	1.00	1.00
No	61	38	3.46 (2.14, 5.58)	2.47 (1.33, 4.60)*
**Trimesters of pregnancy**				
First	21	17	1.41 (0.69, 2.85)	1.01 (0.49, 5.11)
Second	46	114	0.46 (0.29, 0.73)	0.81(0.12, 3.00)
Third	79	90	1.00	1.00
**Number of pregnancies**				
One	32	56	1.00	1.00
Two	56	92	1.06 (0.61, 1.84)	1.77 (0.51, 7.42)

≥ Three	58	73	1.39 (0.79, 2.42)	3.53 (0.85, 14.43)
**History of abortion**				
Yes	66	50	2.82 (1.79, 4.44)	3.33 (1.33, 6.05)*
No	80	171	1.00	1.00
**Awareness about effect of alcohol**				
Yes	69	175	1.00	1.00
No	77	46	4.25 (2.68, 6.72)	4.66 (2.53, 8.61)*
**Family social support**				
Have no family social Support	65	58	2.25 (1.45, 3.51)	2 (1.14, 3.53)*
Have family social support	81	163	1.00	1.00

Note. * refers significant association

Discussion

The overall prevalence of alcohol consumption among pregnant women was 39.78% (34.74, 44.99). The finding from this study is lower than Ethiopia Demographic and Health Survey (45%) [52]. However, the prevalence of alcohol consumption reported in this study was higher than

research conducted in Jimma hospital and Bahir Dar dwellers which are 31.3% and 34%, respectively [48,53]. The possible reason could be the time of the study; population difference, and study design. A study conducted in Bahir Dar was community-based, unlike the current study which was institution-based.

This study's alcohol consumption prevalence was also higher than the findings from some of the African countries such as 16% in Uganda [54], 19.6% in South Africa [21], 25% in Uganda [22], 32.5% in Congo [23], 20.4% in Ghana [55] and 22.6% in South Nigeria [38].

Similarly, the magnitude from this study was higher compared to some studies in western countries like the United Kingdom (28.5%), Russia (26.5%) and Switzerland (20.9%) [19]. However, it is in line with a study conducted in Geneva that showed around 36.3% of the women drank at least one glass of alcohol during pregnancy [20]. In contrast to the above, alcohol consumption in this study was far lower compared to the results in Nigeria (59.3%) [26], Ghana (48%) [24], and Australia (56%) [25].The observed discrepancy might be due to the type of alcohol consumed, study period, awareness and perception about the effect of alcohol on the fetus, and socio-cultural difference among the participants.

The magnitude of binge drinking, which is known for its hazardous effect on the fetus, was 3.54% (1. 89, 5.98). It was similar to the findings to the studies in America (3.1%) [28], Canada (3%) [27], and South Africa (4.6%) [29]. However, it was much lower than the findings from studies in New Zealand (9%) [30], Uganda (10%), Congo (20.16%) [31], and Congo (25.42%) [23].

Another pattern of alcohol consumption, hazardous drinking which is found as a risk factor for abortion and miscarriage, was also determined in this study. It has still an immense negative health effect on the fetus. Of those pregnant women who consumed alcohol, 4.9% (2.03, 6.65) of them drank four or more unit of alcohol per week. Similarly, 11.7% of pregnant women in this study

reported as they drank 2 to 3 unit of alcohol per week. Around 14% of participants were drinking 2 to 3 units of alcohol per month. A similar study conducted in New Zealand revealed 10% of pregnant women drank 7 units or more of alcohol within a week which was higher than the current study [30]. The discrepancy of the magnitude of binge drinking and amount of alcohol taken per week observed might be due to socio-demographic difference, study design, year of study, and awareness and perception about the effect of alcohol on the fetus.

This study also showed a significant association between not having formal education and alcohol consumption. It is in line with findings from previous studies [38, 53, and 56]. It could be due to non-educated women might not have awareness on the harmful effect of alcohol consumption. However, another study showed that highly educated pregnant women were more likely to consume alcohol [32].

The occupation was also significantly associated with alcohol consumption. Being a housewife was shown as risky for alcohol consumption compared to being employed and working their businesses. This finding is supported by other studies [48, 57]. It could be explained by housewives dependent behavior on their spouse that could create distress and their usual engagement in a routine home activity that includes preparing traditional alcohol beverages. Whereas, studies in European countries revealed that employed pregnant women are more likely prone to alcohol consumption [32]. Again, pregnant women lacking awareness about the harmful effect of alcohol on the fetus were more likely to consume higher levels of alcohol than pregnant women who have the awareness. This finding is supported by other similar studies [36, 38, 55]. The other important factor significantly associated with alcohol consumption is family social support. According to the current study, pregnant women who had not family social support had higher odds of alcohol

consumption which is consistent with other studies [33, 34]. But, a study conducted in Boston, Massachusetts revealed that social support is not a factor for alcohol consumption [58].

Moreover, a woman whose pregnancy was unplanned and had abortion history had a higher chance of consuming alcohol. This finding is also in line with the result of other studies [35–37, 48]. The possible reason could be that grief due to the previous abortion, psychological and physiological effect of abortion that might eventually cause distress and alcohol consumption.

Unlike some previous studies [35,38,59,60], the current study didn't show a significant association between women age, gestational age, gravidity, ethnicity, number of alive children, religion, average monthly income, previous alcohol use, chat use, and cigarette smoking with alcohol consumption of pregnant women. Even though alcohol consumption is culturally accepted, the amount and frequency of alcohol drinking may be under-reported by pregnant women because of the possible perceived stigma and recall bias during the interview. Likewise, estimating the amount of alcohol drank by participants and changing the different type of alcohol into the standard unit was challenging and may not be accurate. It is also a real challenge and considered as a limitation for alcohol and other substances study across the world that includes the current research.

Conclusions

This study revealed a higher magnitude of alcohol consumption among pregnant women. The prevalence of binge drinking and the amount and frequency of drinking per week among pregnant women was also higher. Factors such as not being educated, being housewife, having unplanned current pregnancy, having a history of abortion, not having awareness about the effect of alcohol on the fetus and not having family social support were significantly associated with alcohol

consumption. Interventions focused on creating awareness about the harmful effects of alcohol on pregnancy; strengthening social support, and family planning services have to be developed and implemented. Moreover, we recommend to future researchers to conduct longitudinal study considering devices that help to measure the amount of alcohol in the blood.

List of abbreviations

ANC: Ante Natal Care; AOD: Alcohol and Other Drugs; AOR: Adjusted Odds Ratio; COR: Crude Odds Ratio; CDC: Center for Disease Control; CI: Confidence Interval; EDHS: Ethiopian; Demographic and Health Survey; ETB: Ethiopian Birr; FASS: Fetal Alcohol Spectrum Syndrome; HC: Health Center; MSPSSf: Multidimensional Scale of Perceived family Social Support; WHO: World Health Organization

Acknowledgements

We would like to acknowledge Addis Ababa University, School of public health for their courage to begin this program and keep it to be continued.
We also thank the staffs, and plan and program officers of Kolfe Keranio sub-city health centers for providing the necessary information, and their permission and support to conduct this research.

References

1. Odejide AO. Status of drug use/abuse in Africa: A review. Int J Ment Health Addict. 2006;4(2):87–102.
2. Organization WH. Atlas on substance use (2010): resources for the prevention and treatment of substance use disorders. Geneva: World Health Organization; 2010.

3. Poznyak V. The impact of alcohol consumption on global health. Tackling Harmful Alcohol Use. 2015;81.

4. McGee CL, Bjorkquist OA, Riley EP, Mattson SN. Impaired language performance in young children with heavy prenatal alcohol exposure. Neurotoxicol Teratol. 2009;31(2):71–5.

5. Kalberg WO, Provost B, Tollison SJ, Tabachnick BG, Robinson LK, Eugene Hoyme H, et al. Comparison of motor delays in young children with fetal alcohol syndrome to those with prenatal alcohol exposure and with no prenatal alcohol exposure. Alcohol Clin Exp Res. 2006;30(12):2037–45.

6. Goldschmidt L, Richardson GA, Cornelius MD, Day NL. Prenatal marijuana and alcohol exposure and academic achievement at age 10. Neurotoxicol Teratol. 2004;26(4):521–32.

7. Linell A, Richardson MX, Wamala S. The Swedish national public health policy report 2010. Scand J Public Health. 2013;41(10_suppl):3–56.

8. Kesmodel U, Wisborg K, Olsen SF, Brink Henriksen T, Jørgen Secher N. Moderate alcohol intake in pregnancy and the risk of spontaneous abortion. Alcohol Alcohol. 2002;37(1):87–92.

9. Harlap S, Shiono PH. Alcohol, smoking, and incidence of spontaneous abortions in the first and second trimester. Lancet. 1980;316(8187):173–6.

10. Rasch V. Cigarette, alcohol, and caffeine consumption: risk factors for spontaneous abortion. Acta Obstet Gynecol Scand. 2003;82(2):182–8.

11. Kesmodel U, Wisborg K, Olsen SF, Henriksen TB, Secher NJ. Moderate alcohol intake during pregnancy and the risk of stillbirth and death in the first year of life. Am J Epidemiol. 2002;155(4):305–12.

12. Nykjaer C, Alwan NA, Greenwood DC, Simpson NAB, Hay AWM, White KLM, et al. Maternal alcohol intake prior to and during pregnancy and risk of adverse birth outcomes: evidence from a British cohort. J Epidemiol Community Heal. 2014;68(6):542–9.
13. Kesmodel U. Binge drinking in pregnancy—frequency and methodology. Am J Epidemiol. 2001;154(8):777–82.
14. Bailey BN, Delaney-Black V, Covington CY, Ager J, Janisse J, Hannigan JH, et al. Prenatal exposure to binge drinking and cognitive and behavioral outcomes at age 7 years. Am J Obstet Gynecol. 2004;191(3):1037–43.
15. Alati R, Al Mamun A, Williams GM, O'Callaghan M, Najman JM, Bor W. In utero alcohol exposure and prediction of alcohol disorders in early adulthood: a birth cohort study. Arch Gen Psychiatry. 2006;63(9):1009–16.
16. Manning MA, Hoyme HE. Fetal alcohol spectrum disorders: a practical clinical approach to diagnosis. Neurosci Biobehav Rev. 2007;31(2):230–8.
17. Seiler NK. Alcohol and pregnancy: CDC's health advice and the legal rights of pregnant women. Public Health Rep. 2016;131(4):623–7.
18. Butt P, Beirness D, Gliksman L, Paradis C, Stockwell T. Alcohol and health in Canada: a summary of evidence and guidelines for low-risk drinking. Ottawa, Can Cent Subst Abus. 2011;
19. Mårdby A-C, Lupattelli A, Hensing G, Nordeng H. Consumption of alcohol during pregnancy—A multinational European study. Women and Birth. 2017;30(4):e207–13.
20. Dupraz J, Graff V, Barasche J, Etter J-F, Boulvain M. Tobacco and alcohol during pregnancy: prevalence and determinants in Geneva in 2008. Swiss Med Wkly. 2013;143(2122).

21. Petersen Williams P, Jordaan E, Mathews C, Lombard C, Parry CDH. Alcohol and other drug use during pregnancy among women attending midwife obstetric units in the Cape Metropole, South Africa. Adv Prev Med. 2014;2014.

22. Namagembe I, Jackson LW, Zullo MD, Frank SH, Byamugisha JK, Sethi AK. Consumption of alcoholic beverages among pregnant urban Ugandan women. Matern Child Health J. 2010;14(4):492–500.

23. Barthélémy T, Andy M, Roger M. Effect of maternal alcohol consumption on gestational diabetes detection and mother-infant's outcomes in Kinshasa, DR Congo. Open J Obs Gynecol. 2011;1(4):208–12.

24. Lekettey JDP, Dako-Gyeke P, Agyemang SA, Aikins M. Alcohol consumption among pregnant women in James Town Community, Accra, Ghana. Reprod Health. 2017;14(1):120.

25. Alcohol | Australian Government Department of Health [Internet]. [cited 2020 Jun 17]. Available from: https://www.health.gov.au/resources/pregnancy-care-guidelines/part-c-lifestyle-considerations/alcohol

26. Ordinioha B, Brisibe S. Alcohol consumption among pregnant women attending the ante. natal clinic of a tertiary hospital in South. South Nigeria. Niger J Clin Pract. 2015;18(1):13–7.

27. Popova S, Lange S, Probst C, Parunashvili N, Rehm J. Prevalence of alcohol consumption during pregnancy and Fetal Alcohol Spectrum Disorders among the general and Aboriginal populations in Canada and the United States. Eur J Med Genet. 2017;60(1):32–48.

28. Tan CH, Denny CH, Cheal NE, Sniezek JE, Kanny D. Alcohol use and binge drinking among women of childbearing age—United States, 2011–2013. Morb Mortal Wkly Rep.

2015;64(37):1042–6.

29. Popova S, Lange S, Probst C, Shield K, Kraicer- Melamed H, Ferreira- Borges C, et al. Actual and predicted prevalence of alcohol consumption during pregnancy in the WHO African Region. Trop Med Int Heal. 2016;21(10):1209–39.

30. Ho R, Jacquemard R. Maternal alcohol use before and during pregnancy among women in Taranaki, New Zealand. New Zeal Med J. 2009;122(1306).

31. Williams AD, Nkombo Y, Nkodia G, Leonardson G, Burd L. Prenatal alcohol exposure in the Republic of the Congo: prevalence and screening strategies. Birth Defects Res Part A Clin Mol Teratol. 2013;97(7):489–96.

32. Alcohol and Pregnancy: Attitudes Around the Globe [Internet]. [cited 2020 Jun 17]. Available from: https://womensmentalhealth.org/posts/alcohol-pregnancy-attitudes-around-globe/

33. Haynes G, Dunnagan T, Christopher S. Determinants of alcohol use in pregnant women at risk for alcohol consumption. Neurotoxicol Teratol. 2003;25(6):659–66.

34. Barnet B, Duggan AK, Wilson MD, Joffe A. Association between postpartum substance use and depressive symptoms, stress, and social support in adolescent mothers. Pediatrics. 1995;96(4):659–66.

35. Isaksen AB, Østbye T, Mmbaga BT, Daltveit AK. Alcohol consumption among pregnant women in Northern Tanzania 2000–2010: a registry-based study. BMC Pregnancy Childbirth. 2015;15(1):205.

36. Sedgh G, Singh S, Hussain R. Intended and unintended pregnancies worldwide in 2012 and recent trends. Stud Fam Plann. 2014;45(3):301–14.

37. Coleman PK, Reardon DC, Cougle JR. Substance use among pregnant women in the context

of previous reproductive loss and desire for current pregnancy. Br J Health Psychol. 2005;10(2):255–68.

38. Onwuka CI, Ugwu EO, Dim CC, Menuba IE, Iloghalu EI, Onwuka CI. Prevalence and predictors of alcohol consumption during pregnancy in South-Eastern Nigeria. J Clin diagnostic Res JCDR. 2016;10(9):QC10.

39. Demaio AR, Dugee O, De Courten M, Bygbjerg IC, Enkhtuya P, Meyrowitsch DW. Exploring knowledge, attitudes, and practices related to alcohol in Mongolia: a national population-based survey. BMC Public Health. 2013;13(1):1–7.

40. Sudhinaraset M, Wigglesworth C, Takeuchi DT. Social and cultural contexts of alcohol use: Influences in a social–ecological framework. Alcohol Res Curr Rev. 2016;

41. Coles CD, Black MM. Introduction to the special issue: impact of prenatal substance exposure on children's health, development, school performance, and risk behavior. J Pediatr Psychol. 2006;31(1):1–4.

42. Seyoum G, Gebre A. Rapid assessment of the situation of drug and substance abuse in selected urban areas in Ethiopia. Addis Ababa, AAU. 1995;9–55.

43. Wedajo Lemi B. Microbiology of Ethiopian traditionally fermented beverages and condiments. Int J Microbiol. 2020;2020.

44. Narcotic Manual final – EFDA [Internet]. [cited 2020 Jun 18]. Available from: http://www.fmhaca.gov.et/publication/narcotic-manual-final/

45. Abebe H. Isolation and Characterization of the Dominant Yeast in the Traditional beverages of Ethiopia; Tella and Tej. Addis Ababa Universty; 2011.

46. Von Elm E, Altman DG, Egger M, Pocock SJ, Gøtzsche PC, Vandenbroucke JP. The Strengthening the Reporting of Observational Studies in Epidemiology (STROBE)

statement: guidelines for reporting observational studies. Ann Intern Med. 2007;147(8):573–7.
47. Kolfe Keranio Sub City - aaca [Internet]. [cited 2020 Jun 15]. Available from: http://www.addisababa.gov.et/de/web/guest/kolfe-keranio-sub-city
48. Anteab K, Demtsu B, Megra M. Assessment of Prevalence and Associated Factors of Alcohol Use during Pregnancy among the dwellers of Bahir-Dar City, Northwest Ethiopia, 2014. 2014;
49. Zimet GD, Dahlem NW, Zimet SG, Farley GK. The multidimensional scale of perceived social support. J Pers Assess. 1988;52(1):30–41.
50. Nakigudde J, Musisi S, Ehnvall A, Airaksinen E, Agren H. Adaptation of the multidimensional scale of perceived social support in a Ugandan setting. Afr Health Sci. 2009;9(2).
51. Saunders JB, Aasland OG, Babor TF, De la Fuente JR, Grant M. Development of the alcohol use disorders identification test (AUDIT): WHO collaborative project on early detection of persons with harmful alcohol consumption- II. Addiction. 1993;88(6):791–804.
52. Statistical Agency C, International I. Ethiopia 2011 Demographic and Health Survey.
53. Mohammed MA, Ahmed JH, Bushra AW, Aljadhey HS. Medications use among pregnant women in Ethiopia: a cross sectional study. J Appl Pharm Sci. 2013;3(4):116.
54. English L, Mugyenyi GR, Ngonzi J, Kiwanuka G, Nightingale I, Koren G, et al. Prevalence of Ethanol Use Among Pregnant Women in Southwestern Uganda. J Obstet Gynaecol Canada JOGC= J d'obstetrique Gynecol du Canada JOGC. 2015;37(10):901–2.
55. Adusi-Poku Y, Edusei AK, Bonney AA, Tagbor H, Nakua E, Otupiri E. Pregnant women

and alcohol use in the Bosomtwe district of the Ashanti region-Ghana. Afr J Reprod Health. 2012;16(1).

56. Demographic N. Health Survey 2011. Addis Ababa, Ethiop. 2012;

57. Onah MN, Field S, van Heyningen T, Honikman S. Predictors of alcohol and other drug use among pregnant women in a peri-urban South African setting. Int J Ment Health Syst. 2016;10(1):38.

58. McNAMARA TAYK, Orav EJ, Wilkins-Haug L, Chang G. Social support and prenatal alcohol use. J Women's Heal. 2006;15(1):70–6.

59. Muckle G, Laflamme D, Gagnon J, Boucher O, Jacobson JL, Jacobson SW. Alcohol, smoking, and drug use among Inuit women of childbearing age during pregnancy and the risk to children. Alcohol Clin Exp Res. 2011;35(6):1081–91.

60. Skeen S, Lund C, Kleintjes S, Flisher A, Consortium MhRP. Meeting the millennium development goals in Sub-saharan Africa: what about mental health? Int Rev Psychiatry. 2010;22(6):624–31.

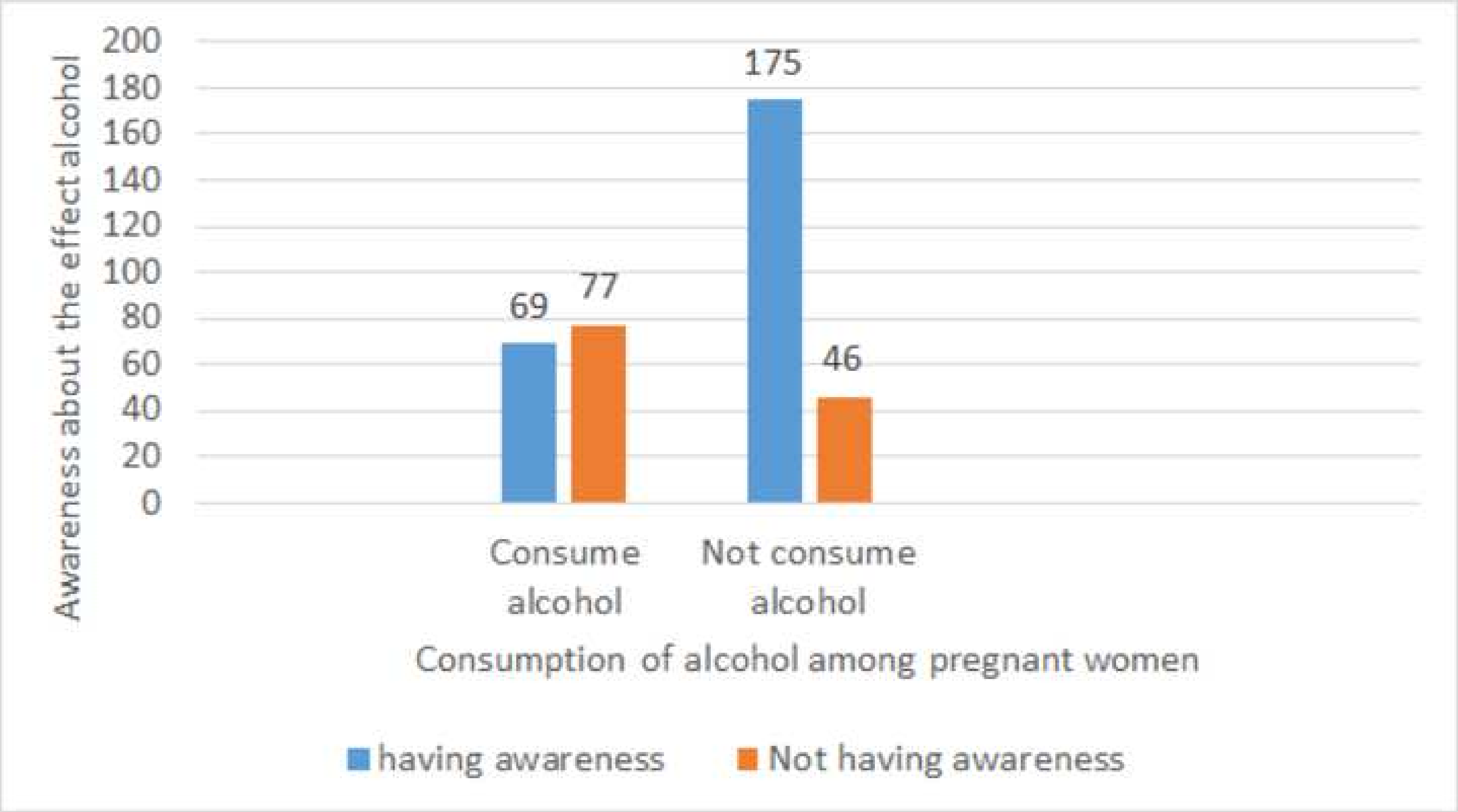
Awareness about the effect alcohol
200
180
160
140
120
100
80
60
40
20
0
69
77
175
46
Consume alcohol
Not consume alcohol
Consumption of alcohol among pregnant women
having awareness
Not having awareness

Figure

Click here to access/download;Figure;Fig 1..tif

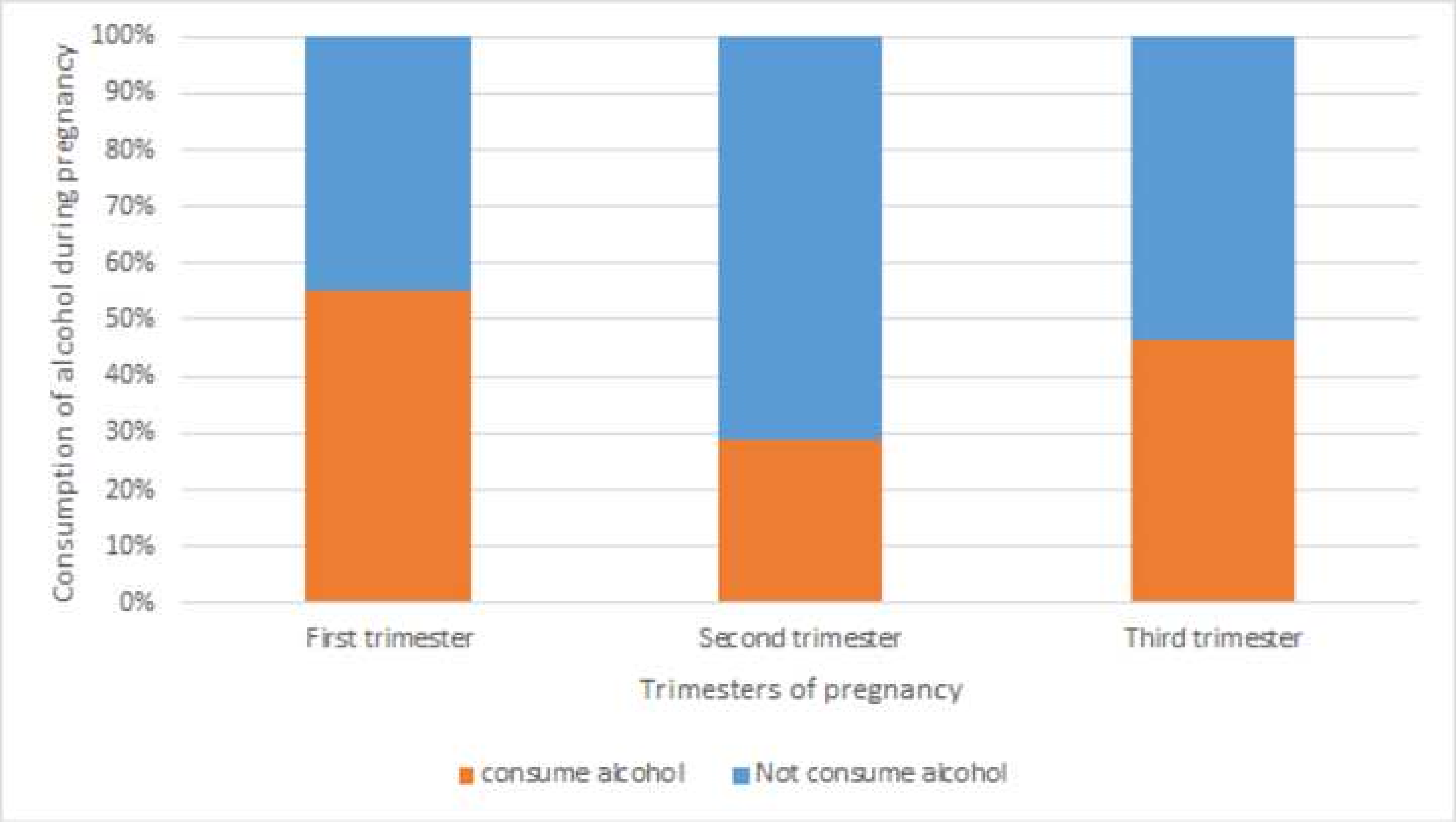

Click here to access/download
Supporting Information
Consumption and binge drinking of alcohol.docx

Consumption of alcohol and binge drinking among pregnant women in ~~health institutions,~~ Addis Ababa, Ethiopia: prevalence and determinant factors

Mezinew Sintayehu Bitew[1*], Maereg Fekade Zewude[2], Muluken Wubetu[3], Addisu Alehegn Alemu[4]

[1]Department of mental health, Debre Markos University, Debre Markos, Amhara, Ethiopia

[2] Department of Maternal and Child Health, Addis Ababa City Administration Health Bureau, Addis Ababa, Ethiopia

[3]Department of Pharmacy, Debre Markos University, Debre Markos, Amhara, Ethiopia.

[4]Department of midwifery, Debre Markos University, Debre Markos, Amhara, Ethiopia.

* Corresponding Author

E-mail: sintayehume@gmail.com (MS)

Abstract

Introduction: People in Ethiopia, including pregnant women, highly consume both home-made and manufactured alcohol beverages due to of lack of awareness about the harmful effect of risky alcohol use, and cultural acceptance of alcohol consumption. Alcohol consumption and other hazardous patterns of use like binge drinking have tremendous adverse effects on both the fetus and mothers. Therefore, this study aimed to assess the magnitude of alcohol consumption, binge drinking and its determinants factors among pregnant women resideingresiding in Kolfe sub-city, Addis Ababa, Ethiopia.

Methods: Institutional based cross-sectional study was conducted on among a total of 367 pregnant women. once they had beenThe participants were selected by using a systematic random sampling method. Data was were collected using through a structured questionnaire. A binary logistic regression model, usingwas conducted using SPSS version 20 software, was used to identify associated factorsdeterminants of alcohol consumption and binge drinking. A p-value < 0.05 was used to declare a statistical significance in multiple logistic regression. The results were described using adjusted odds ratio with a 95% confidence interval., and statistical significance was considered at p-value < 0.05.

Results: This study revealed that the prevalence of alcohol consumption, binge drinking, and weekly alcohol consumption of four or more units among pregnant women was 39.78%, 3.54% and 4.9%, respectively. Factors such as Nnot having formal education [AOR 95% CI=8.47 (2.42, 29.62), having primary education [AOR 95% CI=4.26 (1.23, 14.74), being a housewife [AOR 95% CI= 4.18 (2.13, 8.22), having an unplanned pregnancy [AOR 95% CI=2.47(1.33, 4.60), having a history of abortion [AOR 95% CI=3.33 (1.33, 6.05)], not having awareness about the harmful effect of alcohol consumption [AOR 95% CI=4.66 (2.53, 8.61)], and not having family social

support [AOR 95% CI= 2(1.14,3.53) were determinants of ~~found to be significantly associated with~~ alcohol consumption among pregnant women.

Conclusions: This study found ~~that there is~~ a high level of alcohol consumption among pregnant women. ~~Prevention and i~~Interventions ~~that focuses on~~to ~~creating~~ create awareness ~~about~~ on the harmful effects of alcohol are needed.~~,~~ Moreover, strengthening social support during pregnancy~~,~~ and family planning ~~policy and~~ services ~~that can~~to reduce unplanned pregnancy and abortion should be considered.~~have developed and implemented.~~

Keywords: Alcohol, Consumption, Binge, Drinking, ~~Predictors~~Determinants, Pregnancy, Ethiopia

Introduction

Globally, use of substances such as alcohol, tobacco and other illicit substances, in a way that could harm the users' health, have escalated through time in all segments of the population and has become one of the growing public health and socioeconomic concerns. ~~From these substances, alcohol~~ Alcohol shares the ~~biggest~~ highest burden that accounts for 3.8% of all global deaths and 4.6% of the global burden of diseases and neuropsychiatric disorders [1–3]. Risky use of alcohol causes significant morbidity and mortality (particularly from injuries) and societal harm such as social disruption from crime, unemployment and marital disharmony [2, 3].

~~In pregnant women, t~~The negative health consequences of alcohol consumption are more profound for ~~themselves~~ pregnant women ~~as well as for the~~ and their fetuses. Alcohol consumption during pregnancy has ~~is~~ also shown~~ed~~ to be one of the leading causes of preventable birth defects and developmental problems ~~including~~comprising language and motor delays and deprived ~~poor~~ academic achievement [4–6].

New studies indicate that even low levels of prenatal alcohol exposure could adversely affect the developing fetus. Cognitive and socio-emotional deficits, among children exposed to even small amounts of alcohol, are found [7]. Consumption of ~~<~~_ 2 or more standard drink of alcohol/day in early pregnancy is associated with significant problems on the fetus. ~~that~~ It can cause miscarriage , preterm birth~~,~~ and low birth weight ~~and low birth centile~~ compared to children born from non-drinkers. In addition, other studies reported that women who consumed more than three drinks per week during the first trimester had a significant ~~negative~~harmful health effect on their pregnancy~~,~~ .~~and~~ T~~t~~he ~~effect~~ consequence would be five folds more if they took five or more units of alcohol per week [8–12]. Furthermore, binge- drinking (commonly defined as consuming 4 or more units of alcohol at one occasion for women) during pregnancy is more harmful because of ~~the~~ its serious toxic effects on fetal neurodevelopment ~~after it crosses~~ crossing the placenta [13, 14].

The ~~Adverse~~ adverse effects of prenatal alcohol exposure can range from subtle developmental problems, or fetal alcohol effects, to full-blown fetal alcohol spectrum syndrome (FASS). However, ~~the effects of alcohol exposure are~~it is not limited to infancy and childhood; prenatal exposure to alcohol, particularly in early pregnancy, has also been found to increase the likelihood of developing an alcohol disorder in adulthood [15, 16].

Given these consequences and the belief that there is no safe time or amount to consume alcohol in pregnancy, the CDC recommends complete abstinence [17, 18].

~~Despite this,~~Different studies ~~from various high income and low-income countries~~ have reported ~~a high rate of~~ alcohol consumption ~~by~~ among pregnant women ranges -from 19.5% in South Africa ~~to~~ and ~~36.3~~59 % in ~~Geneva~~ Nigeria [19-26]~~. The prevalence showed that 26.5%, 28.5%, 36.3%, 19.6%, 25%, and 32.5% in Russia, United Kingdom, South Africa Uganda and Congo respectively~~

[19-23]. The Higher prevalence is also reported from countries like Ghana, Australia, and Nigeria (48%, 56% and 59% respectively) [24–26].

Similarly, studies conducted on the prevalence of binge drinking among pregnant women showed between 3% in Canada and 25% in Congo Studies on the prevalence of binge drinking among pregnant women also reported that ranges from 3% in Canada [27] to 25% far high in Congo [3, 27-31]. The amount of drinking per week, which is the other clinically significant patterns of alcohol use, revealed in literature likeA study in New Zealand study also which states thatrevealed 10% of pregnant women drank seven and more units of alcohol per week which is the other clinically significant patterns of alcohol use as well [30].

According to Studies studies from different countries showed that socio-demographic factors (age, marital status, educational status, and occupation), obstetric history (gestational age, unplanned pregnancy, gravidity, and history of abortion), social support and behavioural issues like use of Khat and cigarette smoking were predictors determinants for theof alcohol consumption of alcohol during pregnancy [32–37]. In addition Additionally, studies reported that awareness about the harmful effect of alcohol on fetus, attitude and culture of the population were the determinants factors for alcoholof alcohol consumption [36, 38–40].

Because of the rapid increase of AAlcohol consumption and its terrific associated terrific problems among pregnant women across the world, studies of high-income countries on alcohol and its effect are increased, and the interventions for alcohol problems are also developingincreased, globally [41].

In low-income countries including Ethiopia, the rapid increase of the consumption of alcohol has also been indicated around twofor decades ago, and it is still increasing alarmingly and, especially reproductive-age women of fertile age have are affected by alcohol [1, 42]. Both traditional and

commercially produced alcohol beverages are widely spread more than ever which are also an important contributing factors for increased alcohol consumption in Ethiopia~~with the face of lack of awareness about the harmful effect of alcohol, and other predictors of alcohol consumption~~ [1, 38].

People in Ethiopia that include pregnant women highly consume alcohol beverages, especially the ~~culturally accepted~~ traditionally prepared alcohol beverages like Areki, Teje, Tella that ~~–~~ have ~~a~~ higher percentages of alcohol amount [43–45]. Thus, locally made alcohols are ~~produced~~ being used excessively without control ~~and are usually used~~ in different ~~events like celebrities, ceremonies~~of ~~,~~ holidays, religious festivals, parties, and almost in ~~-~~daily family meals and day to day life [43–45].

Despite the adverse health effects of alcohol consumption is profound~~the above mentioned problems~~, its assessment ~~of alcohol consumption~~ with ~~its~~ determinants ~~factors upon~~ among pregnant women is limited. In particular, t~~T~~here is~~–~~ a scarcity of ~~study~~ information on binge drinking of alcohol by measuring or calculating the amount ~~of alcohol~~ consumed. Addis Ababa, the capital city of Ethiopia where the current study ~~is~~ was conducted, is heavily affected ~~by hazardous and harmful~~with consumption of alcohol ~~due to both modernization and the deeply rooted cultural acceptance of alcohol consumption~~ [1].~~-~~Therefore, ~~given the above background,~~ this study was intended to assess the magnitude of alcohol consumption and binge drinking among pregnant women, and its determinants ~~factors~~ in Kolfe sub city, Addis Ababa, Ethiopia. The finding of this study will have significant contributions for health program managers, policymakers, clinicians and researchers.

Materials and Methods

Study design, -sample size and sampling technique

An iInstitutional-based quantitative cross-sectional study was conducted based on the guidelines of Strengthening the Report of Observational Studies in Epidemiology (STROBE) for observational research [46]. It was conducted at Kolfe Keranio sub-city, Addis Ababa, Ethiopia. Kolfe KeranioThe sub-city, with an covers area of 61.25 sq.km and, has a total population of 546, 219 (235, 360 were females) in 2017. Among the female population, around Around 2.33 % (9,531) of females were pregnant. The sub-city has 15 districts and 11 Health Centers (HCs) [47]. A total of 397 pregnant mothers women were included in a this study. after calculating by using single Single population proportion formula was considered to calculate the sample size. $(n = Z_{a/2}^2 * p * (1-p)/d2)$ by Ttaking 34% proportion prevalence (p) of alcohol use consumption of among pregnant women (34%) [48], 5% margin of error (d), with 95% confidence interval (CI), and by adding 15%, the assumption of non-response rate. Systematic random sampling with 'k' value 20 was carried out after the samples were was proportionally allocated to each of the following 11 health centers: Woreda 1 HC (600*), Woreda 3 HC (1002*), Alem Bank HC (960*), Kolfe HC (846*), Woreda 5 HC (716*), Woreda 6 HC (426*), Keranio HC (798*), Woreda 11 HC (832*), Lomi Meda HC(540*), Woreda 9 HC (470*), and Mikililand HC (880*). All pPregnant women, who regardless of the gestational age, were in all trimesters, and attended the above mentioned health centers during the study period were included in the study. However, pPregnant women who were seriously ill and unable to respond were excluded. The study was conducted from May to June 2017.

Data collection ~~questionnaire/~~instrument~~s~~ and procedure

~~Questions~~Data regarding on participants' socio-demographic, obstetric characteristics ~~variables as well as those on~~, alcohol consumption, awareness about the harmful effect of alcohol and the use of other substances ~~like~~ (i.e. Khat chewing and cigarette smoking) were ~~gathered, adapted and~~collected using structured questionnaire developed from ~~various~~ previous literatures. However, ~~Family~~ family social support was assessed ~~by~~ through the Multidimensional Scale of Perceived Social Support (MSPSSf). MSPSS has three subscales (family, friends and significant others), with a total of 12 items each scaled from 1 (very strongly disagree) to 7 (very strongly agree) [49, 50]. Respondents scored higher than 14 from a multidimensional scale of perceived family social support (MSPSSF) 7 Likert scale were recognized as having family social support, those who scored less than or equal to 14 were not having social support.

~~Questionnaire regarding the~~Likewise, the amount and frequency of alcohol consumption per day, week, and month~~, and generally in the~~ during the time of pregnancy was adapted from AUDIT and other articles [13, 21, and 51]. In Ethiopian, both traditional (e.g. Tella, Araki, Teje) and manufactured (Beer, Wine) alcohol beverages are widely produced and consumed. People used local equipments called Tella Birecheko, Melekia, and Birelie ~~to drink traditional alcohols~~ that can measure around 340 ml, 40 ml and 270 ml ~~volume~~, respectively to drink traditional ~~,~~alcohols. Pregnant women were ~~also~~ asked to estimate the average number of drinks they consumed. Finally, the data were standardized using the percentage and volume of alcohol in each ml of beverages.~~, and the data was standardized into a standard unit of alcohol, by using the percentage of alcohol and volume in ml~~. ~~In this study, alcohol consumption was defined as a pregnant woman who drank any amount of alcohol during the period of pregnancy.~~ Whereas, drinking four more units of alcohol on one occasion was considered binge drinking.

Six questions were developed to assess the awareness of the harmful effect of alcohol on fetus, and those who answered the six questions correctly identified as having awareness about the consequences of ~~alcohol.~~

~~Furthermore~~alcohol. Furthermore, any amount of khat use and cigarette smoking even at once during the current pregnancy is known as pregnant women who chew khat and smoke cigarette, respectively~~.~~ The questionnaire was prepared initially in English and translated to Amharic then back to English to check the consistency of the questions.

~~The questionnaire was prepared initially in English and translated to Amharic then back to English to check the consistency of the questions~~. ~~The training was given to the data collectors and supervisors for three days about the data collection tool, way of interview, the ethical principles of confidentiality and data management before their involvement with data collection.~~

Eleven BSc nurses ~~with BSc degree qualification~~ as data collector~~s to collect data~~ and two public health ~~professionals~~ officers as supervisor~~s to assist and monitor the data collection process~~ were ~~employed~~participated during data collection. Ethical clearance was obtained from Addis Ababa University, public health school ethical review committee. The right participants were systematically selected by data collectors when pregnant women came to maternal care. Data collectors explained the objective and procedures of the study, and their right to refuse or discontinue the interview at any time to participants. And, they knew that the questionnaire was anonymous and their privacy kept confidential. Also, Participants were aware of as the study has not any risk or direct benefit like incentives for them; it was only conducted for the achievement of objectives, and ultimately for improving health services of pregnant women and the community as a whole. After participants heard and understood all the necessary information and consent statement, they have been asked about their willingness to be interviewed, which was verbal

consent. Those volunteers were again asked to write their name and put their signature with the date of interview in a prepared blank space found below the information sheet and statement of consent, which was written consent. For participants under 18 years old, informed consent was also obtained from their parents.

After that, data collectors interviewed the selected participant in the face to face manner by using a structured questionnaire at the waiting room of the maternal care clinic that took around 15 minutes. Those who found risky of alcohol consumption has been linked to substance abuse clinic, and responsible leaders in selected health centers and maternal care clinics were recommended and noticed to initiate and strengthen prevention of alcohol consumption among pregnant women. ~~To control the quality of data, on top of providing training for data collectors and supervisors, supervision was held regularly during the data collection period, and the collected data were checked daily for completeness and consistency. And, Manual and computerized data cleaning were done before data analysis.~~

Data quality, ~~-~~processing and analysis

To assure the data quality training was given to the data collectors and supervisors for three days about the data collection tool, way of interview, the ethical principles of confidentiality before their involvement to the data collection. The collected data were checked for its completeness and consistency daily. Then, the collected d~~D~~ata were checked, cleaned and entered into Epi.DATA 3.1 version~~s~~ and ~~then~~ exported ~~into~~ to Statistical Package for the Social Sciences (SPSS) ~~window~~ version 20 for analysis. Descriptive statistics were summarizedy~~y~~ using frequencies, percentage, graphs, the mean and standard deviation. ~~were used to present study results.~~ Binary logistic regression was employed ~~to calculate the adjusted odds ratio (AOR)~~ to ~~identify~~ assess the association of each independent ~~determinant factors~~variables with ~~of~~ the outcome variable. All

~~Variables~~ variables ~~which~~ with ~~met~~ p-value < 0.2 during ~~bivariate~~ binary logistic regression ~~analysis~~ were ~~exported to~~considered in the ~~multivariate~~ multiple logistic regression. ~~analysis model.~~ The findings were ~~described~~ presented ~~with~~ using Odds ratio (crude and adjusted) with its ~~corresponding~~ 95% ~~Confidence interval~~CIs. Moreover, a p~~P~~-value ~~of~~ < 0.05 was considered ~~as~~ to declare the variable was statistically significant during the final ~~multivariable binary~~multiple logistic regression ~~model~~analysis.

Results and discussion

Results

Description of socio-demographic characteristics

A total of 367 pregnant women were interviewed making 92.44% ~~The~~ response rate. ~~was 92.44%.~~ ~~Out of 367 pregnant women~~This study revealed almost three fourth~~,~~ (74.7%) ~~(274)~~of participants were married. Seven out of ten ~~Two hundred sixty~~ (70.8%) participants were non-educated, ~~and~~ whereas women who completed secondary education and above were 26 (7.1%). The age of participants ranged between 15 and 49 with ~~The~~ mean age ~~of respondents was~~ 27.43 (SD ±~~±~~ ~~SD~~4.77)~~5 years (ranging from 15-49).~~

~~In terms of~~years. Regarding to ethnicity, Amhara and Oromo comprise the majority, 114 (31.1%) and 107(29.2%) respectively. Nearly ~~to~~ half of the participants , ~~117~~(48.2%) were followers of orthodox ~~Christian~~ religion followed by Muslims (35.7%). Majority (42.5%) of pregnant women ~~(42.5%)~~ were housewives. Similarly, ~~while about~~ one-third of ~~them~~ participants' ~~had a~~ monthly average income was between 1001-2000 ETB **(Table 1)**.

Table 1: Socio-demographic characteristics of pregnant women in Kolfe Keranio sub city (n=367), Addis Ababa, Ethiopia

Variables	Frequency	Percent (%)
**Age**		
15-19	8	2.2
20-24	100	27.2
25 -29	117	31.9
3029-34	106	28.9
≥>35	36	9.8
Mean (SD)	27.43±4.775	
**Marital status**		
Married	247	67.3
Ssingle	81	22.1
Divorced	31	8.4
Widowed	8	2.2
**Ethnicity**		
Amhara	114	31.1
Oromo	107	29.2
Gurage	91	24.8
Tigrie	32	8.7
Others	23	6.3
**Religion**		
Orthodox	177	48.2
IslamMuslim	131	35.7
Protestant	40	10.9

Formatted Table

Catholic	10	2.7
Others	9	2.5
**Educational status**		
Not educated	127	34.6
Primary ~~educated~~education	123	33.5
Secondary ~~educated~~education	91	24.8
~~>~~Above ~~Secondary~~ secondary ~~educated~~education	26	7.1
**Occupation**		
Employed	105	28.6
Housewife	156	42.5
Own business	106	28.9
**Average monthly house hold income ~~of the house hold~~**		
≤ 1000 Birr	44	12
1001-2000	109	29.7
2001-3000	76	20.7
3001-4000	61	16.6
>4000	77	21

Assessment of obstetric history of respondents

As shown in **Table 2** below, the majority of the pregnant women ~~148~~(40.3%) were in the second trimester. Almost three fourth~~quarter~~ (73%) of the pregnant women ~~have~~ had a planned pregnancy.

~~Two hundred fifty-one~~Three or more out of ten (~~68.4~~31.6%) of pregnant women had ~~no~~ history of abortion.

Table 2: Obstetric history of the ~~respondents among~~ pregnant women in kolfe Keranio sub city (n=367) Addis Ababa, Ethiopia

Variables	Frequency	Percent ~~(%)~~
**Trimester of pregnancy**		
First ~~timester~~trimester	38	10.4
Second trimester ~~Third trimester~~	148	40.3
Third trimester	131	35.7
**Number of pregnancy(gravidity)**		
~~First time~~One	88	24
~~Second time~~Two	148	40.3
≥~~>~~ T~~t~~hree ~~times~~	131	35.7
**Number of alive children**		
0~~No child~~	112	30.5
One	134	36.5
Two	65	17.7
> T~~t~~wo	56	15.3
**Planned pregnancy**		
Yes	268	73
No	99	27
**History of abortion**		
Yes	116	31.6

Formatted Table

No	251	68.4

Though most of the participants were in the second and third trimester, the proportion of alcohol consumption value was high among first trimester 21 (55.26%) **(Fig 1)**.

Fig. 1: Alcohol consumption among pregnant women respected to trimesters of pregnancy in Kolfe Keranio sub city (n=367), Addis Ababa, Ethiopia

Alcohol consumption, binge drinking and other substances during pregnancy

As illustrated in **(Table 3)** below, 146(39.8%) of pregnant women drank alcohol. The majority (44.4%) of participants predominately drank Tella. Fifty-four (14.71%) of participants consumed alcohol 2-4 times per month while 15 (4.09%) of pregnant women drank four and above times per week. On the other hand, 13 (3.54%) of ~~them~~ women had the experience of binge drinking during their pregnancy.

Table 3: Alcohol consumption and binge drinking among pregnant women in kolfe sub city (n=367), Addis Ababa, Ethiopia

Variables	Frequency	Percent ~~(%~~)
**Alcohol consumption before pregnancy**		
Yes	179	48.8
No	188	51.2
**Alcohol consumption during pregnancy**		
Yes	146	39.8
No	221	60.2
**Binge drinking during pregnancy**		
Yes	13	3.54

Formatted: Font: Not Bold

Formatted Table

No	354	96. 46
**Frequency of alcohol consumption during pregnancy**		
≤< once a month	36	9.81
2-4 times per month	54	14.71
2-3times per week	41	11.17
≥ 4 4 and above pertimes per week	15	4.09
**Type of alcohol use**		
Tella	65	44.4
Teje	9	6.3
Areki	14	9.7
Beer	41	28.5
Wine	9	6.3
Others	7	4.9
**Cigarette smoking during pregnancy**		
Yes	10	2.7
No	357	97.3
**Khat use during pregnancy**		
Yes	39	10.6
No	328	89.4

Awareness about the harmful effect of alcohol use

As demonstrated in **Fig 2**, Two hundred forty-four (66.5%) pregnant women have awareness, answered all six questions correctly regarding the harmful effect of alcohol consumption. Among

those who have awareness, only 69 (28.28%) consumed alcohol. Whereas, of those who answered the awareness questions incorrectly, 76 (62.6%) drank alcohol.

Fig 2: Magnitude of alcohol consumption among pregnant women respected to awareness about the harmful effect of alcohol on fetus in Kolfe Keranio sub city (n=367), Addis Ababa, Ethiopia

Factors associated with alcohol consumption among pregnant women

As shown in **Table 4**, educational status, occupation, plan of pregnancy, history of abortion, awareness about the harmful effect of alcohol, and family social support of pregnant women were significant predictors of alcohol consumption among pregnant women. ~~with p-value<0.05. Statistically, significant~~The higher odds alcohol consumption ~~rate~~ was observed among non-educated and attained primary education pregnant women ~~and those who found in primary education compared to pregnant women who completed secondary education and above~~ with (AOR 95% CI= 8.47 (2.42, 29.62) and AOR 95% CI= 4.26 (1.23, 14.74), respectively compared to those completed secondary education and above .

Pregnant women who were ~~in some form of occupation appeared to have a decreased risk of alcohol consumption compared to those who were~~housewives had a fourfold risk of alcohol consumption ~~with~~ (AOR 95% CI=4.18 (2.13, 8.22) compared to employed women. ~~Respondents~~ Women ~~who have had unplanned~~whose ~~current~~ last pregnancy was unplanned had a two folds ~~prone to~~risk of alcohol consumption than ~~who had planned pregnancy~~their counters (AOR 95% CI=2.47(1.33, 4.60). Likewise,

~~Those~~ pregnant women ~~that~~ who had a history of abortion ~~experienced~~ were prone to three-fold risk for alcohol consumption compared to pregnant women who hadn't ~~a history of abortion with~~ [AOR 95% CI=3.33 (1.33, 6.05)]. Women who ~~answered correctly~~had awareness about the effect

of alcohol on fetus and them also found to be a protective factor for alcohol consumption compared to those who answered incorrectly which become a risk factorhad no awareness [AOR 95% CI 4.66 (2.53, 8.61)]. Moreover, in assessment family social support, more than a half (66.5%) of them scored greater than or equal to 14 from the total score of MSPSSf. And theThis study also found a relationship between family social support and alcohol consumption; pregnant women who hadn't family social support were had two-folds riskss for alcohol consumption [AOR 95% CI= 2 (1.14, 3.53) compared to those who had family social support.

Table 4: Factors associated Determinants of alcohol use ofconsumption among pregnant women in Kolfe Keranio sub city (n=367), Addis Ababa, Ethiopia (n=367)

Formatted: Font: Not Bold

Formatted Table

	Alcohol consumption during pregnancy			
**Variables**	**Yes**	**No**	**Crude OR (95% CI)**	**Adjusted OR (95% CI)**
**Religion**				
Orthodox	76	101	1.00	1.00
Islam	43	88	0.65 (0.41, 1.04)	1.25 (042, 2.13)
Protestant	24	16	1.99 (0.99, 4.01)	3.01 (0.96, 5.02)
Catholic	2	8	0.33 (0.69, 1.61)	0.23 (0.11, 1.71)
Others	1	8	0.166 (0.02, 1.36)0	7.09 (-0.68, 18.4)
**Educational status**				
Not educated	65	62	4.40 (1.56, 12.40)	8.47 (2.42, 29.62)*
Primary education	61	62	4.13 (1.47, 11.66)	4.26 (1.23, 14.74)*
Secondary education	15	76	0.83 (0.27, 2.54)	1.41 (0.38, 5.23)
	5	21	1.00	1.00

~~≥~~Above secondary education				
**Occupation**				
Employed	34	71	1.00	1.00
housewife	87	69	2.63 (1.57, 4.41)	4.18 (2.13, 8.22)*
own business	25	81	0.65 (0.35, 1.18)	0.76 (0.35, 1.66)
**Plan of pregnancy**				
Yes	85	183	1.00	1.00
No	61	38	3.46 (2.14, 5.58)	2.47 (1.33, 4.60)*
**Trimesters of pregnancy**				
First	21	17	1.41 (0.69, 2.85)	1.01 (0.49, 5.11)
Second	46	114	0.46 (0.29, 0.73)	0.81(0.12, 3.00)
Third	79	90	1.00	1.00
**Number of pregnancies**				
~~First time~~One	32	56	1.00	1.00
~~Second time~~Two	56	92	1.06 (0.61, 1.84)	1.77 (0.51, 7.42)
≥~~>~~ T~~t~~hree ~~times~~	58	73	1.39 (0.79, 2.42)	3.53 (0.85, 14.43)
**History of abortion**				
Yes	66	50	2.82 (1.79, 4.44)	3.33 (1.33, 6.05)*
No	80	171	1.00	1.00

**Awareness about effect of alcohol**				
~~Having awareness~~ Yes	69	175	~~----------~~1.00	~~--------------------~~1.00
~~Not having awareness~~No	77	46	4.25 (2.68, 6.72)	4.66 (2.53, 8.61)*
**Family social support**				
Have no family social Support	65	58	2.25 (1.45, 3.51)	2 (1.14, 3.53)*
Have family social support	81	163	~~------------------~~1.00	~~--------------~~1.00

Note. * refers significant association ~~OR=Odds ratio CI=Confidence Interval~~

Discussion

The overall prevalence of alcohol consumption among pregnant women was 39.78% ~~with 95% CI~~ (34.74, 44.99)~~, which met the criteria of consuming any amount of alcohol during the current pregnancy period~~. The finding ~~of~~ from this study is lower than Ethiopia Demographic and Health Survey ~~((EDHS, 2011),~~ 45%) [52]. However, the prevalence of alcohol consumption reported in this study was higher than research conducted in Jimma hospital and Bahir ~~dar~~ Dar dwellers ~~that~~ which are 31.3% and 34%~~.~~, respectively [48,53]. The possible reason could be the time of the study; population ~~variation~~difference, and study design. A study conducted in ~~particular for~~ Bahir D~~d~~ar ~~study that~~ was community-~~based~~ based unlike the current study which was institution based~~study~~.

This study's ~~prevalence of~~ alcohol consumption prevalence was also higher than ~~the magnitude of~~ the findings from ~~-~~some of the African countries such as 16% in Uganda [54], 19.6% in South Africa [21], 25% in Uganda [22], 32.5% in Congo [23], 20.4% in Ghana [55] and 22.6% in South Nigeria [38]. Similarly, the magnitude ~~of~~ from this study was higher compared to some studies in western countries like the United Kingdom (28.5%), Russia (26.5%) and Switzerland (20.9%) [19]. However, it is in line with a study conducted in Geneva that showed around 36.3% of the women drank at least one glass of alcohol during pregnancy [20]. In contrast to the above, the ~~prevalence of~~ alcohol consumption of this study ~~is~~ was far lower compared to the results in Nigeria (59.3%) [26], Ghana (48%) [24], and Australia (56%) [25]. ~~-~~The observed discrepancy ~~observed~~ might be ~~because of~~due to the type of alcohol consumed, ~~year of the~~ study period, awareness and perception about the effect of alcohol on the fetus, and socio-cultural difference among the participants.

The magnitude of binge drinking ~~(drinking four units and more at one occasion),~~, which is known ~~by~~ for ~~the~~ its hazardous effect on the fetus, ~~, was still significant in this study which is~~ was 3.54% ~~with 95% CI~~ (1. 89, 5.98). It was similar with the findings to ~~However, it did not differ from~~ the studies in America (3.1%) [28], Canada (3%) [27], and South Africa (4.6%) [29]. ~~Whereas,~~However, ~~binge drinking of this study (3.54%) is~~it was much lower than the findings from studies ~~shown~~ in New Zealand (9%) [30], Uganda (10%), Congo ~~study~~ (20.16%) ~~in 2013~~ [31], and Congo (25.42%) [23].

Another ~~hazardous~~ pattern of alcohol consumption, hazardous drinking~~,~~ which is found as a risk factor for abortion and miscarriage ~~according to previous studies~~, was also determined in this study. It has still an immense negative health effect on the fetus. ~~-~~Of those pregnant women who consumed alcohol, 4.9% ~~with 95% CI~~ (2.03, 6.65) of them drank four or more unit of alcohol per

week. ~~And actually~~Similarly, 11.7% of pregnant women in this study reported as they drank 2 to 3 unit of alcohol per week. ~~which has still an immense negative health effect on fetus evidenced from the previous study. Majority of~~Around 14% ~~participants~~of participants ~~14%~~ were drinking 2 to 3 units of alcohol per month. ~~But, a~~ A similar study conducted in New Zealand ~~study~~ revealed ~~that~~ 10% of pregnant women drank 7 units or more of ~~alcohol and more within~~alcohol within ~~one~~ a week which ~~is~~ was higher than ~~our~~ the current study [30]. The discrepancy of the magnitude of binge drinking and amount of alcohol taken per week observed might be due to sociodemographic difference ~~in the culture of society~~, study design ~~and method of data collection~~, year of study, and awareness and perception about the effect of alcohol on the fetus. ~~, and other socio-demographic characteristics amongst studies.~~

This study also showed ~~that there was a statistically~~a significant association between not having formal education and alcohol consumption. ~~which~~ It ~~was~~ is in line with findings ~~with~~ from previous studies [38, 53, 56]. It could be due to n~~N~~on-educated women might not have ~~no experience in reading and be un~~awareness ~~of~~ on the harmful effect of alcohol consumption. ~~But~~However, ~~the opposite finding was observed in another study; it~~another study ~~stated that~~showed that highly educated pregnant women ~~are~~ were more likely to consume ~~risky for~~ alcohol ~~consumption~~ [32].

The occupation was also a significant~~ly~~ ~~associated with~~factor for alcohol consumption. Being a housewife was shown as risky ~~for alcohol~~for alcohol consumption compared to being employed women and~~/or~~ working their own businesses. This finding is supported by other studies [48, 57]. It could be explained by housewives dependent behaviour on their spouse that could create distress ~~in routine home activity~~, and their usual engagement in routine home activity ~~in~~ including preparing traditional alcohol beverages ~~and during ceremonies~~. ~~Whereas~~However, a stud~~ies~~y ~~in~~

~~European countries~~ revealed~~, that~~ employed pregnant women are more likely ~~prone~~ to consume alcohol ~~consumption~~ [32].

~~The finding in this study showed that~~Again, pregnant women lacking awareness about the harmful effect of alcohol on the fetus ~~experienced~~ were more likely to consume higher levels of alcohol ~~consumption~~ than pregnant women who ~~having~~ have the awareness~~,~~ T~~t~~his finding is supported by other ~~previous~~ similar studies [36, 38, 55]. The other important ~~variable significantly~~factor significantly associated with alcohol consumption is family social support. According to the current study, ~~Those~~ pregnant women who had~~ve~~ not family social support had higher odds of ~~is predictive of~~ alcohol consumption which is consistent with other studies [33, 34]. But, a study conducted in Boston, Massachusetts revealed that social support is not a factor for alcohol consumption [58].

Moreover, a~~A~~ woman ~~having~~ whose ~~unplanned~~ pregnancy was unplanned and had abortion history ~~of abortion~~ had a higher chance of consuming alcohol. ~~was also reported to practice more alcohol consumption~~ This finding ~~which~~ is also in line with the finding ~~of~~ from other studies [35–37, 48]. The possible reason could be ~~related to~~that grief ~~about~~ due to the previous ~~abortion and psychological~~abortion, psychological and physiological effect of abortion which might eventually ~~may~~ cause distress and alcohol consumption.

Unlike some previous studies [35,38,59,60], the current ~~This~~ study didn't show ~~the~~ a significant association between women age, gestational age~~ of participant~~, ~~gravidity, and ethnicity~~gravidity, ethnicity, number of alive children, religion, average monthly income, previous alcohol use~~ of pregnant women~~, chat use, and cigarette smoking with alcohol consumption of pregnant women.~~,~~ ~~unlike some previous studies where those variable differences were observed [35,38,59,60].~~

Even though alcohol consumption is culturally accepted~~ for all socio-demographic~~, the amount and frequency of alcohol drinking may be under-reported by pregnant women because of the possible perceived stigma and possible recall bias ~~forgetfulness ~~during the interview~~ing~~. Likewise, ~~Also, to ~~estimatinge the~~ average~~ amount of alcohol drank ~~for ~~by ~~-~~participants and ~~interviewer during the process of ~~changing the different type of alcohol into the standard unit was ~~difficult to~~challenging and may not be accurate. ~~which ~~This ~~also~~is also a real challenge and considered as a limitation for~~of~~ alcohol and other substances stud~~yies~~ across the world that includes~~ing~~ the current study~~ across the world~~.

Conclusions

This ~~finding ~~study ~~observed ~~revealed a higher ~~prevalence ~~magnitude of alcohol consumption among pregnant women.~~,~~ T~~t~~he~~ significant~~ magnitude of binge drinking~~,~~ and ~~a serious~~the amount and frequency of drinking per week among pregnant women was also high.~~seen among pregnant women in Kolfe Keranio sub-city that really requires an effort of prevention and intervention~~. Factors such as not being educated, being housewife, having unplanned current pregnancy, having a history of abortion, not having awareness about the effect of alcohol on the fetus and not having family social support were significantly associated with alcohol consumption ~~of pregnant women~~. ~~Guidelines and screening tools have to be developed and integrated into the health care system. Prevention ~~I~~i~~nterventions ~~that ~~focuse~~d~~s on creating awareness about the harmful effects of alcohol on pregnancy; strengthening social support, and family planning ~~policy and ~~services ~~that could reduce unplanned pregnancy and abortion ought to ~~have to be developed and implemented. Moreover, we recommend to future researchers ~~the ~~to conduct l~~L~~ongitudinal study ~~that needs daily~~

~~or weekly follows up with~~ considering ~~a supporting~~ devices that ~~able~~ help to measure the amount of alcohol in the blood. ~~is necessary for the future researcher.~~

List of abbreviations

ANC~~————————~~: Ante Natal Care

AOD~~——————~~: Alcohol and Other Drugs

AOR: Adjusted Odds Ratio

COR: Crude Odds Ratio

CDC~~——————~~: Center for Disease Control

CI: Confidence Interval

~~FASS——Fetal Alcohol Spectrum Syndrome~~

EDHS~~—~~: Ethiopian Demographic and Health Survey

ETB: Ethiopian Birr

FASS: Fetal Alcohol Spectrum Syndrome

HC~~–~~: Health Center

MSPSSf~~–~~: Multidimensional Scale of Perceived family Social Support

WHO~~–~~: World Health Organization

Acknowledgements

We would like to acknowledge Addis Ababa University, School of public health for their courage to begin this program and keep it to be continued.

We also thank ~~also~~ the staffs, and plan and program officers of Kolfe Keranio sub-city health centers for providing the necessary information ~~to determine the sample size of the study~~, and their permission and support~~ing~~ to conduct this research. ~~My appreciation also extends to my family and friends for their remarkable support and help in many aspects including finance to complete the study.~~

References

1. Odejide AO. Status of drug use/abuse in Africa: A review. Int J Ment Health Addict. 2006;4(2):87–102.
2. Organization WH. Atlas on substance use (2010): resources for the prevention and treatment of substance use disorders. Geneva: World Health Organization; 2010.
3. Poznyak V. The impact of alcohol consumption on global health. Tackling Harmful Alcohol Use. 2015;81.
4. McGee CL, Bjorkquist OA, Riley EP, Mattson SN. Impaired language performance in young children with heavy prenatal alcohol exposure. Neurotoxicol Teratol. 2009;31(2):71–5.
5. Kalberg WO, Provost B, Tollison SJ, Tabachnick BG, Robinson LK, Eugene Hoyme H, et al. Comparison of motor delays in young children with fetal alcohol syndrome to those with prenatal alcohol exposure and with no prenatal alcohol exposure. Alcohol Clin Exp Res. 2006;30(12):2037–45.
6. Goldschmidt L, Richardson GA, Cornelius MD, Day NL. Prenatal marijuana and alcohol exposure and academic achievement at age 10. Neurotoxicol Teratol. 2004;26(4):521–32.
7. Linell A, Richardson MX, Wamala S. The Swedish national public health policy report

2010. Scand J Public Health. 2013;41(10_suppl):3–56.

8. Kesmodel U, Wisborg K, Olsen SF, Brink Henriksen T, Jørgen Secher N. Moderate alcohol intake in pregnancy and the risk of spontaneous abortion. Alcohol Alcohol. 2002;37(1):87–92.

9. Harlap S, Shiono PH. Alcohol, smoking, and incidence of spontaneous abortions in the first and second trimester. Lancet. 1980;316(8187):173–6.

10. Rasch V. Cigarette, alcohol, and caffeine consumption: risk factors for spontaneous abortion. Acta Obstet Gynecol Scand. 2003;82(2):182–8.

11. Kesmodel U, Wisborg K, Olsen SF, Henriksen TB, Secher NJ. Moderate alcohol intake during pregnancy and the risk of stillbirth and death in the first year of life. Am J Epidemiol. 2002;155(4):305–12.

12. Nykjaer C, Alwan NA, Greenwood DC, Simpson NAB, Hay AWM, White KLM, et al. Maternal alcohol intake prior to and during pregnancy and risk of adverse birth outcomes: evidence from a British cohort. J Epidemiol Community Heal. 2014;68(6):542–9.

13. Kesmodel U. Binge drinking in pregnancy—frequency and methodology. Am J Epidemiol. 2001;154(8):777–82.

14. Bailey BN, Delaney-Black V, Covington CY, Ager J, Janisse J, Hannigan JH, et al. Prenatal exposure to binge drinking and cognitive and behavioral outcomes at age 7 years. Am J Obstet Gynecol. 2004;191(3):1037–43.

15. Alati R, Al Mamun A, Williams GM, O’Callaghan M, Najman JM, Bor W. In utero alcohol exposure and prediction of alcohol disorders in early adulthood: a birth cohort study. Arch Gen Psychiatry. 2006;63(9):1009–16.

16. Manning MA, Hoyme HE. Fetal alcohol spectrum disorders: a practical clinical approach

to diagnosis. Neurosci Biobehav Rev. 2007;31(2):230–8.

17. Seiler NK. Alcohol and pregnancy: CDC's health advice and the legal rights of pregnant women. Public Health Rep. 2016;131(4):623–7.

18. Butt P, Beirness D, Gliksman L, Paradis C, Stockwell T. Alcohol and health in Canada: a summary of evidence and guidelines for low-risk drinking. Ottawa, Can Cent Subst Abus. 2011;

19. Mårdby A-C, Lupattelli A, Hensing G, Nordeng H. Consumption of alcohol during pregnancy—A multinational European study. Women and Birth. 2017;30(4):e207–13.

20. Dupraz J, Graff V, Barasche J, Etter J-F, Boulvain M. Tobacco and alcohol during pregnancy: prevalence and determinants in Geneva in 2008. Swiss Med Wkly. 2013;143(2122).

21. Petersen Williams P, Jordaan E, Mathews C, Lombard C, Parry CDH. Alcohol and other drug use during pregnancy among women attending midwife obstetric units in the Cape Metropole, South Africa. Adv Prev Med. 2014;2014.

22. Namagembe I, Jackson LW, Zullo MD, Frank SH, Byamugisha JK, Sethi AK. Consumption of alcoholic beverages among pregnant urban Ugandan women. Matern Child Health J. 2010;14(4):492–500.

23. Barthélémy T, Andy M, Roger M. Effect of maternal alcohol consumption on gestational diabetes detection and mother-infant's outcomes in Kinshasa, DR Congo. Open J Obs Gynecol. 2011;1(4):208–12.

24. Lekettey JDP, Dako-Gyeke P, Agyemang SA, Aikins M. Alcohol consumption among pregnant women in James Town Community, Accra, Ghana. Reprod Health. 2017;14(1):120.

25. Alcohol | Australian Government Department of Health [Internet]. [cited 2020 Jun 17]. Available from: https://www.health.gov.au/resources/pregnancy-care-guidelines/part-c-lifestyle-considerations/alcohol

26. Ordinioha B, Brisibe S. Alcohol consumption among pregnant women attending the ante. natal clinic of a tertiary hospital in South. South Nigeria. Niger J Clin Pract. 2015;18(1):13–7.

27. Popova S, Lange S, Probst C, Parunashvili N, Rehm J. Prevalence of alcohol consumption during pregnancy and Fetal Alcohol Spectrum Disorders among the general and Aboriginal populations in Canada and the United States. Eur J Med Genet. 2017;60(1):32–48.

28. Tan CH, Denny CH, Cheal NE, Sniezek JE, Kanny D. Alcohol use and binge drinking among women of childbearing age—United States, 2011–2013. Morb Mortal Wkly Rep. 2015;64(37):1042–6.

29. Popova S, Lange S, Probst C, Shield K, Kraicer- Melamed H, Ferreira- Borges C, et al. Actual and predicted prevalence of alcohol consumption during pregnancy in the WHO African Region. Trop Med Int Heal. 2016;21(10):1209–39.

30. Ho R, Jacquemard R. Maternal alcohol use before and during pregnancy among women in Taranaki, New Zealand. New Zeal Med J. 2009;122(1306).

31. Williams AD, Nkombo Y, Nkodia G, Leonardson G, Burd L. Prenatal alcohol exposure in the Republic of the Congo: prevalence and screening strategies. Birth Defects Res Part A Clin Mol Teratol. 2013;97(7):489–96.

32. Alcohol and Pregnancy: Attitudes Around the Globe [Internet]. [cited 2020 Jun 17]. Available from: https://womensmentalhealth.org/posts/alcohol-pregnancy-attitudes-around-globe/

33. Haynes G, Dunnagan T, Christopher S. Determinants of alcohol use in pregnant women at risk for alcohol consumption. Neurotoxicol Teratol. 2003;25(6):659–66.

34. Barnet B, Duggan AK, Wilson MD, Joffe A. Association between postpartum substance use and depressive symptoms, stress, and social support in adolescent mothers. Pediatrics. 1995;96(4):659–66.

35. Isaksen AB, Østbye T, Mmbaga BT, Daltveit AK. Alcohol consumption among pregnant women in Northern Tanzania 2000–2010: a registry-based study. BMC Pregnancy Childbirth. 2015;15(1):205.

36. Sedgh G, Singh S, Hussain R. Intended and unintended pregnancies worldwide in 2012 and recent trends. Stud Fam Plann. 2014;45(3):301–14.

37. Coleman PK, Reardon DC, Cougle JR. Substance use among pregnant women in the context of previous reproductive loss and desire for current pregnancy. Br J Health Psychol. 2005;10(2):255–68.

38. Onwuka CI, Ugwu EO, Dim CC, Menuba IE, Iloghalu EI, Onwuka CI. Prevalence and predictors of alcohol consumption during pregnancy in South-Eastern Nigeria. J Clin diagnostic Res JCDR. 2016;10(9):QC10.

39. Demaio AR, Dugee O, De Courten M, Bygbjerg IC, Enkhtuya P, Meyrowitsch DW. Exploring knowledge, attitudes, and practices related to alcohol in Mongolia: a national population-based survey. BMC Public Health. 2013;13(1):1–7.

40. Sudhinaraset M, Wigglesworth C, Takeuchi DT. Social and cultural contexts of alcohol use: Influences in a social–ecological framework. Alcohol Res Curr Rev. 2016;

41. Coles CD, Black MM. Introduction to the special issue: impact of prenatal substance exposure on children's health, development, school performance, and risk behavior. J

Pediatr Psychol. 2006;31(1):1–4.

42. Seyoum G, Gebre A. Rapid assessment of the situation of drug and substance abuse in selected urban areas in Ethiopia. Addis Ababa, AAU. 1995;9–55.

43. Wedajo Lemi B. Microbiology of Ethiopian traditionally fermented beverages and condiments. Int J Microbiol. 2020;2020.

44. Narcotic Manual final – EFDA [Internet]. [cited 2020 Jun 18]. Available from: http://www.fmhaca.gov.et/publication/narcotic-manual-final/

45. Abebe H. Isolation and Characterization of the Dominant Yeast in the Traditional beverages of Ethiopia; Tella and Tej. Addis Ababa Universty; 2011.

46. Von Elm E, Altman DG, Egger M, Pocock SJ, Gøtzsche PC, Vandenbroucke JP. The Strengthening the Reporting of Observational Studies in Epidemiology (STROBE) statement: guidelines for reporting observational studies. Ann Intern Med. 2007;147(8):573–7.

47. Kolfe Keranio Sub City - aaca [Internet]. [cited 2020 Jun 15]. Available from: http://www.addisababa.gov.et/de/web/guest/kolfe-keranio-sub-city

48. Anteab K, Demtsu B, Megra M. Assessment of Prevalence and Associated Factors of Alcohol Use during Pregnancy among the dwellers of Bahir-Dar City, Northwest Ethiopia, 2014. 2014;

49. Zimet GD, Dahlem NW, Zimet SG, Farley GK. The multidimensional scale of perceived social support. J Pers Assess. 1988;52(1):30–41.

50. Nakigudde J, Musisi S, Ehnvall A, Airaksinen E, Agren H. Adaptation of the multidimensional scale of perceived social support in a Ugandan setting. Afr Health Sci. 2009;9(2).

51. Saunders JB, Aasland OG, Babor TF, De la Fuente JR, Grant M. Development of the alcohol use disorders identification test (AUDIT): WHO collaborative project on early detection of persons with harmful alcohol consumption- II. Addiction. 1993;88(6):791–804.

52. Statistical Agency C, International I. Ethiopia 2011 Demographic and Health Survey.

53. Mohammed MA, Ahmed JH, Bushra AW, Aljadhey HS. Medications use among pregnant women in Ethiopia: a cross sectional study. J Appl Pharm Sci. 2013;3(4):116.

54. English L, Mugyenyi GR, Ngonzi J, Kiwanuka G, Nightingale I, Koren G, et al. Prevalence of Ethanol Use Among Pregnant Women in Southwestern Uganda. J Obstet Gynaecol Canada JOGC= J d'obstetrique Gynecol du Canada JOGC. 2015;37(10):901–2.

55. Adusi-Poku Y, Edusei AK, Bonney AA, Tagbor H, Nakua E, Otupiri E. Pregnant women and alcohol use in the Bosomtwe district of the Ashanti region-Ghana. Afr J Reprod Health. 2012;16(1).

56. Demographic N. Health Survey 2011. Addis Ababa, Ethiop. 2012;

57. Onah MN, Field S, van Heyningen T, Honikman S. Predictors of alcohol and other drug use among pregnant women in a peri-urban South African setting. Int J Ment Health Syst. 2016;10(1):38.

58. McNAMARA TAYK, Orav EJ, Wilkins-Haug L, Chang G. Social support and prenatal alcohol use. J Women's Heal. 2006;15(1):70–6.

59. Muckle G, Laflamme D, Gagnon J, Boucher O, Jacobson JL, Jacobson SW. Alcohol, smoking, and drug use among Inuit women of childbearing age during pregnancy and the risk to children. Alcohol Clin Exp Res. 2011;35(6):1081–91.

60. Skeen S, Lund C, Kleintjes S, Flisher A, Consortium MhRP. Meeting the millennium

development goals in Sub-saharan Africa: what about mental health? Int Rev Psychiatry. 2010;22(6):624–31.

~~Supporting information~~

Response to reviewers

First, we thank you for the editors and reviewers!
For both reviewer

1. We modified all the texts of a document and tried to present intelligently and write in a standard English

For reviewer 2
We corrected all the comments and suggestions line by line you provide

1. Line 30-typing error
2. Line 52 and 53- grammar error
3. Line 64-verb problem
4. Line 69-symbol problem
5. Line 87 to 93- comments to remove and correct
6. 102 to104- ambiguous sentence
7. Line 107 and 108-
8. Line 113
9. Line 222
10. Line 228 and 229- it has not a problem; it is the title of figure 1 which should be placed below the paragraph of that message according to the guideline of PLoS one
11. Line 272 and 273- commented to remove

Thank you again!
