# Supplementary material for: Consumption of alcohol and binge drinking among pregnant women in Addis Ababa, Ethiopia: Prevalence and determinant factors
Source: PLoS One. 2020 Dec 22;15(12):e0243784. doi: 10.1371/journal.pone.0243784 (PMC7755214; doi:10.1371/journal.pone.0243784)
Supplement: Attachment — Submitted filename: Response to Reviewers.docx [file pone.0243784.s002.docx]

**Thank you so much all of you, editors and the two reviewers, for reviewing the manuscript critically for the sake of publishing quality scientific research.**

**Response to academic editor’s comment and questions**

1. Please ensure that your manuscript meets PLOS ONE's style requirements, including those for file naming. The PLOS ONE style templates can be found

**Answer: I made the manuscript in line with the requirements of PlOS ONE**

2. For a question -During our internal evaluation we noted that participants provided verbal consent, did the ethics committees/IRBs approve this consent procedure?

**Answer: in the actual procedure of the work, a copy of the master thesis, mother document of this manuscript, it states that both verbal and written informed consent was obtained. Data collectors explained to participants about the objective of the study, procedures, and their right to refuse or discontinue the interview at any time and other important information. After participants heard and understood all the necessary information and consent statement, they have been asked about their willingness to be interviewed, which is verbal consent. Those volunteers were again asked to write their name and put their signature with the date of interview at a prepared blank space found below the information sheet and statement of consent, which is written consent. That means they provide both verbal and written informed consent. So, in the manuscript that states “only verbal consent” was an error and misunderstanding when it was written in research ethics subheading. Now, I accepted your comments and took a correction in this revised manuscript.**

3. Contradictory of the message regarding pregnant women under age 18 in the section of exclusion criteria and ethical consideration.

**Answer: Yes, pregnant women start from 15 years old were enrolled in the study. Therefore, I removed the message that excludes women under 18 years old from the section of exclusion criteria.**

4. In the abstract it is stated that the study “was conducted from April to June 2017”, but in the methods section of the manuscript it is stated that the study “was conducted from May to June 2017”.

**Answer: the right period of data collection was from May to June 2017. I amended it.**

5. Requested to update ORCID ID.

**Answer: I did it.**

**Response for Reviewers**

**Reviewer 1**

1. Are there national survey data (prevalence rates) for alcohol/drug use in the general population that can be included in the Background section, to provide some context for why this is a public health concern in Ethiopia?

**Answer: There are only local single articles in a different part of the country, and all reports that it the public health problems like the other countries across the world. But, there is no national survey study.**

2. Strong comments in method section regarding how data collected, how women approached, how the research described, and about the destination of pregnant women who used alcohol in a risky way

**Answer: I accept all the comments and incorporate the raised concerns.** Data collectors explained the objective and procedures of the study, and their right to refuse or discontinue the interview at any time to participants. Participants were aware of as the study has not any risk or direct benefit like incentives for them; it was only conducted for the achievement of objectives, and ultimately for improving health services of pregnant women and the community as a whole. After that, data collectors interviewed the selected participant in the face to face manner by using a structured questionnaire at the waiting room of the maternal care clinic that took around 15 minutes. Those who found risky of alcohol consumption has been linked to substance abuse clinic, and responsible leaders in selected health centers and maternal care clinics were recommended and noticed to initiate and strengthen prevention of alcohol consumption among pregnant women.

3. A question regarding missing data?

**Answer: From the beginning, we have been adding 10% of possible non-response participants on the calculated samples. The non-responses in a manuscript were refusals to participate but not missing data’s/values. Fortunately, the actual non-response rate was below the prior plan or expected non-response rate that couldn’t affect the analysis and conclusion.**

4. A comment of adding stigma or any other thing that could hinder the disclosure of alcohol use.

Answer: I understand your concern, and I try to add some possible perceived stigma. But, as I mentioned in the background of the study, there is a cultural acceptance of alcohol use in all demographic population. Since alcohol is historical and frequently used in our culture, which may be associated with Orthodox Christianity religion, stigma and punishment are not found.

**Reviewer 2**

**Answer: I saw all questions, comment and suggestion on each page of the document or manuscript that you gave me. I thank you for all you did on a manuscript. And I found it is essential for me and scientific research as well. I accepted all the comments and suggested solutions that you wrote and marked, and I corrected it in a document accordingly. I also received a note that you told me to incorporate the independent sub-heading variable (social support) with the next subheading in table four (associated factors- subheadings). But, I didn’t do for the variable of awareness about the harmful effect of alcohol consumption because this has a figure that describes and presents a descriptive message different from table four (associated factors). And I minimized the method sub-headings and merged it.**
