# Supplementary material for: Consumption of alcohol and binge drinking among pregnant women in Addis Ababa, Ethiopia: Prevalence and determinant factors
Source: PLoS One. 2020 Dec 22;15(12):e0243784. doi: 10.1371/journal.pone.0243784 (PMC7755214; doi:10.1371/journal.pone.0243784)
Supplement: Attachment — Submitted filename: Response to reviewers.docx [file pone.0243784.s004.docx]

**Response to reviewers**

**First, we thank you for the editors and reviewers!**

**For both reviewer**

1. We modified all the texts of a document and tried to present intelligently and write in a standard English

**For reviewer 2**

We corrected all the comments and suggestions line by line you provide

1. Line 30-typing error
2. Line 52 and 53- grammar error
3. Line 64-verb problem
4. Line 69-symbol problem
5. Line 87 to 93- comments to remove and correct
6. 102 to104- ambiguous sentence
7. Line 107 and 108-
8. Line 113
9. Line 222
10. Line 228 and 229- it has not a problem; it is the title of figure 1 which should be placed below the paragraph of that message according to the guideline of PLoS one
11. Line 272 and 273- commented to remove

Thank you again!
