# Supplementary material for: Consumption of alcohol and binge drinking among pregnant women in Addis Ababa, Ethiopia: Prevalence and determinant factors
Source: PLoS One. 2020 Dec 22;15(12):e0243784. doi: 10.1371/journal.pone.0243784 (PMC7755214; doi:10.1371/journal.pone.0243784)
Supplement: Attachment — Submitted filename: Response to reviewers.docx [file pone.0243784.s006.docx]

**Response to reviewers**

**First, we thank you for the editors and reviewers!**

**For both reviewer**

1. We modified all the texts of a document, presented it intelligently and wrote in standard English.
2. We performed statistical analysis appropriately and rigorously.

**For reviewer 2**

- We modified all the texts of a document, presented it intelligently and wrote in standard English depending on your consecutive and valuable strong comments and suggestions you, the reviewers, provide.
- We corrected all the comments and suggestion you provide line by line in the following way.

1. Line 84- we corrected a sentence as you suggest
2. Line 96- we remove an ambiguous and less important adjective word “Terrific”.
3. Line 206-we modified the phrase three-fourth as you comment.

We hope this the time to accept the paper for publication!

Thank you all again!
